# Accurate phenotypic classification and exome sequencing allow identification of novel genes and variants associated with adult-onset hearing loss

**DOI:** 10.1371/journal.pgen.1011058

**Published:** 2023-11-27

**Authors:** Morag A. Lewis, Jennifer Schulte, Lois Matthews, Kenneth I. Vaden, Claire J. Steves, Frances M. K. Williams, Bradley A. Schulte, Judy R. Dubno, Karen P. Steel

**Affiliations:** 1 Wolfson Centre for Age-Related Diseases, King’s College London, United Kingdom; 2 The Medical University of South Carolina, Charleston, South Carolina, United States of America; 3 Department of Twin Research and Genetic Epidemiology, King’s College London, School of Life Course and Population Sciences, London, United Kingdom; National Institute on Deafness and Other Communication Disorders, National Institutes of Health, UNITED STATES

## Abstract

Adult-onset progressive hearing loss is a common, complex disease with a strong genetic component. Although to date over 150 genes have been identified as contributing to human hearing loss, many more remain to be discovered, as does most of the underlying genetic diversity. Many different variants have been found to underlie adult-onset hearing loss, but they tend to be rare variants with a high impact upon the gene product. It is likely that combinations of more common, lower impact variants also play a role in the prevalence of the disease. Here we present our exome study of hearing loss in a cohort of 532 older adult volunteers with extensive phenotypic data, including 99 older adults with normal hearing, an important control set. Firstly, we carried out an outlier analysis to identify genes with a high variant load in older adults with hearing loss compared to those with normal hearing. Secondly, we used audiometric threshold data to identify individual variants which appear to contribute to different threshold values. We followed up these analyses in a second cohort. Using these approaches, we identified genes and variants linked to better hearing as well as those linked to worse hearing. These analyses identified some known deafness genes, demonstrating proof of principle of our approach. However, most of the candidate genes are novel associations with hearing loss. While the results support the suggestion that genes responsible for severe deafness may also be involved in milder hearing loss, they also suggest that there are many more genes involved in hearing which remain to be identified. Our candidate gene lists may provide useful starting points for improved diagnosis and drug development.

## Author summary

Age-related hearing loss is a common disease which is highly heritable, and more than 150 genes are known to underlie human hearing impairment. However, most of these genes are linked to early-onset, severe forms of deafness, and involve variants which are very rare. Investigating age-related hearing loss is challenging because it is so common; there are many genes which may be involved, with multiple contributing variants which may be rare or common. Here, we have obtained audiometric and genomic data from a cohort of older volunteers (aged 55 and over), which includes a vital control group of 99 older people with good hearing. We have taken two approaches to detecting genes and variants implicated in age-related hearing loss, and followed up this work with a second, smaller, cohort. Our study provides multiple candidate genes for further investigation.

## Introduction

Hearing loss is a common, complex condition with a strong genetic component. More than 700 genes have been found to underlie Mendelian hearing loss in humans and/or mice (reviewed in [1]), but large-scale mouse studies suggest there may be as many as 1000 genes which alone can result in hearing impairment when mutated [2]. Identifying the genes and specific gene variants involved in age-related hearing loss may suggest genes or pathways that can be targeted therapeutically, as well as being useful for diagnosis.

Identifying genes and variants involved in hearing loss is challenging owing to the heterogeneity of the disease. The inner ear is a complex system with multiple molecular components that need to function and interact correctly to enable normal hearing. Family studies have led to the identification of many variants involved in adult-onset Mendelian hearing loss (for example, *MIR96* [3], *DMXL2* [4], reviewed in [5]), but these tend to be very rare or even private variants, and are unlikely to explain all of the hearing loss seen in humans. Some loci have been identified through genome-wide association studies (GWAS) [6–8], but very large numbers of people are needed and GWAS chips are limited by their use of common, ancient variants. Whole exome and genome sequencing offer greater scope for identifying causative variants whatever their allele frequency and, indeed, recent studies using exome sequencing [1,9] suggest that intermediate frequency variants also play a role in hearing difficulty.

Another challenge in this field is the complexity of auditory phenotypes. Hearing loss does not have a single pathogenic mechanism, but can result from multiple inner ear pathologies. At present, with few exceptions, accurate diagnosis of the underlying hearing problem is not possible. In addition, many large-scale studies make use of self-reported questionnaires to explore hearing impairment. Although self-reported hearing difficulty is fairly well correlated with overall audiometric thresholds [10–12], and hearing aid prescription is a surrogate for abnormal pure tone audiometry at least in the UK, these may be prone to subjective bias and offer no way to distinguish between different auditory phenotypes and underlying pathologies. Large cohorts with good audiometric phenotyping offer more objective classification of participants, which may allow more sensitive detection of causal genes and variants. This has been demonstrated by a recent study on such a cohort, which found an increased burden of predicted deleterious rare variants in known hearing loss genes in people with sensorineural hearing loss compared to controls with good hearing (assessed by pure-tone threshold) or no medical reports of hearing loss [13]. Furthermore, appropriate quality control is vital for genetic studies, and because adult-onset hearing loss is so common, a well-characterised age-matched group with audiometrically determined normal hearing provides a better control than volunteers reporting no hearing difficulty or younger adults with normal hearing.

Here we present our data from a cohort of older adult volunteers having extensive phenotype data, including 99 older adults with good hearing. The cohort was characterised by estimates of Metabolic and Sensory components of age-related hearing loss, calculated from audiograms [14], then participants were grouped according to their estimates. The Metabolic and Sensory components were based on the premise that underlying cochlear pathologies will have differential effects across audiometric frequencies or at higher frequencies, respectively. More specifically, Metabolic (i.e. strial) losses involve reduced cochlear lateral wall function and Sensory losses involve primary lesions within the organ of Corti or neural components of the cochlea. Different genes may contribute to each primary site of lesion. We have carried out both gene-based and variant-based tests to identify candidate genes and variants in each phenotypic group, and prioritised those candidates using a variety of methods, including repeat analyses in a second, smaller cohort.

## Results

### MUSC cohort classification

**Fig 1:**
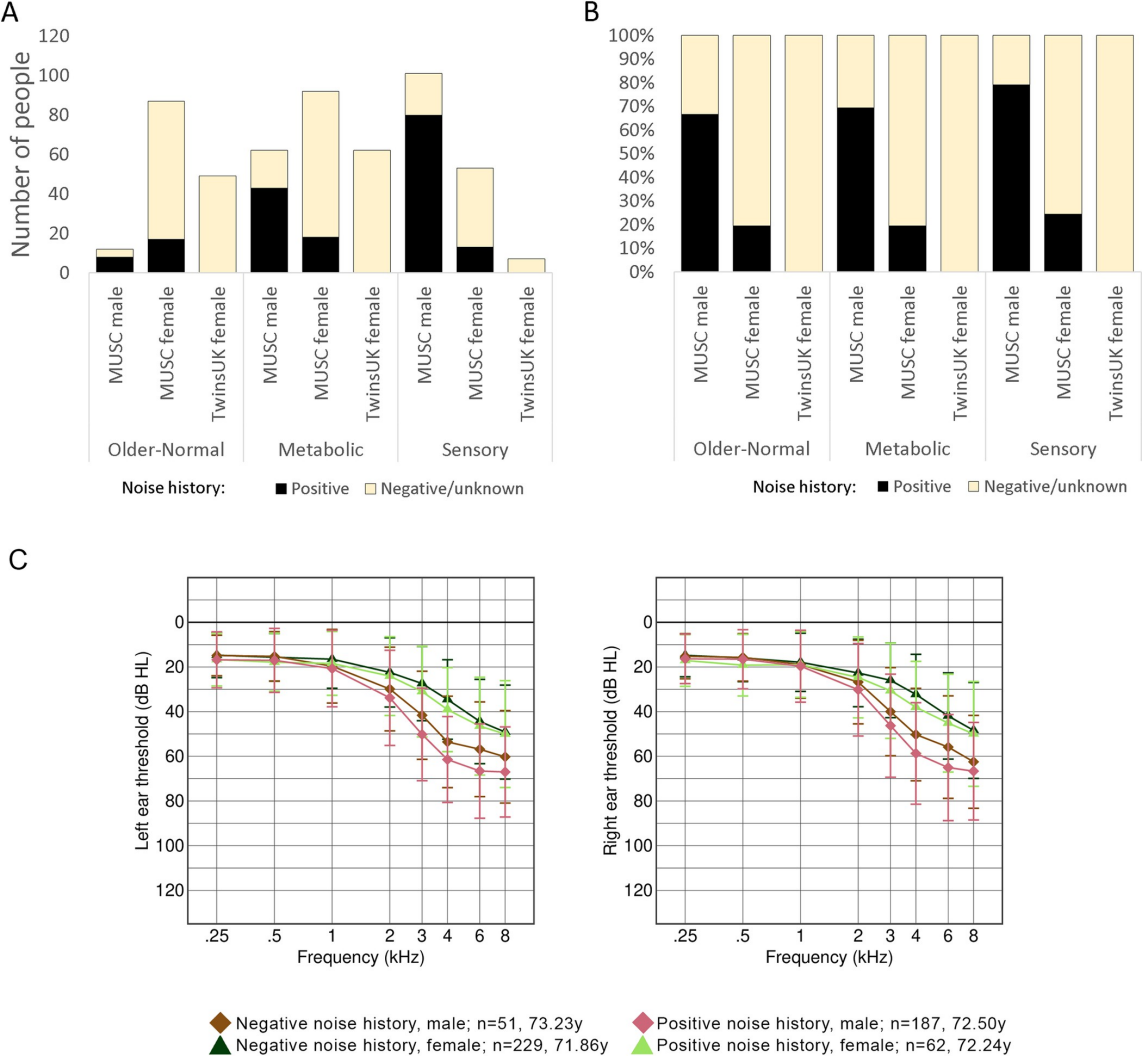
Characteristics of the cohorts in this study. A, B: Bar charts showing the numbers of participants in each classification (not including Unclassified or Unselected cases) in the MUSC and TwinsUK cohorts (with twins removed from the latter). Black sections represent those participants reporting a positive noise history. A shows the numbers, and B shows the percentages reporting a positive noise history within each category. C shows the average audiograms of the MUSC cohort, plotted in groups separated by sex and noise history. The three people with unknown noise history have not been included. The thresholds from the left ear are shown on the left-hand plot, and those from the right ear on the right-hand plot. Numbers and average ages of each group are listed in the key. Error bars are standard deviation.

Our primary cohort consisted of 532 participants; 292 female and 240 male participants, with an overall average age of 72.25 years (71.96 years for women, 72.60 for men). 62 women and 187 men reported a positive noise history (Table 1, Fig 1). The difference in average thresholds between the sexes is greater than that between the groups reporting a positive or negative noise history (Fig 1C). 99 participants were classified into the Older-Normal audiogram category; 87 women and 12 men. In the Metabolic category, there were 92 women and 62 men, while in the Sensory category, there were 53 women and 101 men. PLINK v2 [15] was used to check for relatedness using common variants in linkage disequilibrium; none of the cohort were related. We used the 2504 individuals from the 1000 Genomes study [16] to plot out the ancestry of this cohort and found it to be largely non-Finnish European, most similar to the “British in England and Scotland” and “Utah residents with Northern and Western European ancestry” sub-populations (S1 Fig).

**Table 1:** Details of the cohorts used in this study.

	Entire MUSC cohort		Older-Normal		Metabolic		Sensory		Unclassified		Unselected	
	**Male**	**Female**	**Male**	**Female**	**Male**	**Female**	**Male**	**Female**	**Male**	**Female**	**Male**	**Female**
**Number**	240	292	12	87	62	92	101	53	48	47	17	13
**Average age**	72.6	71.96	66.46	67.71	74.51	74.29	72.32	73.39	72.36	73.57	72.27	71.64
**Positive noise history**	187	62	8	17	43	18	80	13	42	9	14	5
**Unknown noise history**	2	1			1		1	1				
	*Entire TwinsUK cohort*		*Older-Normal*		*Metabolic*		*Sensory*		*Unclassified*		*Unselected*	
	**All**	**Twins removed**	**All**	**Twins removed**	**All**	**Twins removed**	**All**	**Twins removed**	**All**	**Twins removed**	**All**	**Twins removed**
**Number**	159	149	53	49	66	62	7	7	19	17	14	13
**Average age**	64.82	64.69	61.3	61.37	66.48	66.26	68.14	68.14	67.32	66.65	65.29	65.15

### TwinsUK cohort classification

There were 159 female participants from the TwinsUK cohort meeting our requirements, including ten dizygotic twin pairs. There were no monozygotic twin pairs, and no other relatedness was reported. The overall mean age was 64.82 years. There were few positive responses to questions about noise exposure in work or leisure activities, so no participants were classified as having a positive noise history. We carried out the same ancestry analysis on these 159 participants, and found they also had a non-Finnish European ancestry, and like the MUSC cohort, they were most similar to the “British in England and Scotland” and “Utah residents with Northern and Western European ancestry” sub-populations from the 1000 Genomes (S1 Fig).

One twin of each pair was removed from the cohort; where twins were classified into the same category, the removed twin was chosen at random (6 pairs). For 3 pairs, one twin was classified as Older-Normal or Metabolic, with the other either Unclassified or Unselected; in those cases, the Unclassified or Unselected twin was removed. The last pair consisted of one twin classified as Older-Normal and one classified as Metabolic; both were removed for the outlier analysis but for the threshold analysis, one (the Older-Normal-classified twin) was chosen at random for removal. After twin removal, there were 49 in the Older-Normal category (average age 61.37 years), 62 in the Metabolic category (average age 66.26 years) and 7 in the Sensory category (average age 68.14 years) (Table 1, Fig 1).

### Selection and validation of high quality, high impact variants

After quality filtering (S1 Table), 938,008 (MUSC) and 279,434 (TwinsUK) high quality variants were obtained from the nuclear exome, and 1174 (MUSC) and 142 (TwinsUK) mitochondrial variants, most of which were homoplasmic (with a variant allele fraction > 0.95) (S2 Fig). Variants were then filtered for high predicted impact and MAF < 0.1, based on our previous work (S1 Table, [1]), resulting in 29,807 (MUSC) and 21,432 (Twins UK) high quality, high impact variants from the nuclear exome, and 226 (MUSC) and 16 (TwinsUK) high impact mitochondrial variants (S2 Fig). For the two analyses carried out, homoplasmic mitochondrial variants were treated as homozygote calls and heteroplasmic mitochondrial variants as heterozygote calls.

In order to validate the exome sequencing, Sanger sequencing was carried out on 114 variants in multiple samples from the MUSC cohort, and individual call accuracy was 94.7% (360 correct from 380 calls in total). Only two variants were not validated; the remainder of the incorrect calls were errors in zygosity (eg a heterozygote call for an individual homozygous for the alternate allele) (Table 2).

**Table 2:** Validation of variant calls.

		Sanger sequencing		
		Homozygous reference	Heterozygous	Homozygous alternate
Whole-exome calls	Homozygous reference	177	1	0
	Heterozygous	9	141	7
	Homozygous alternate	0	3	38

Number of correct and incorrect variant calls from whole exome sequencing, checked by Sanger sequencing.

### Outlier analysis

**Fig 2:**
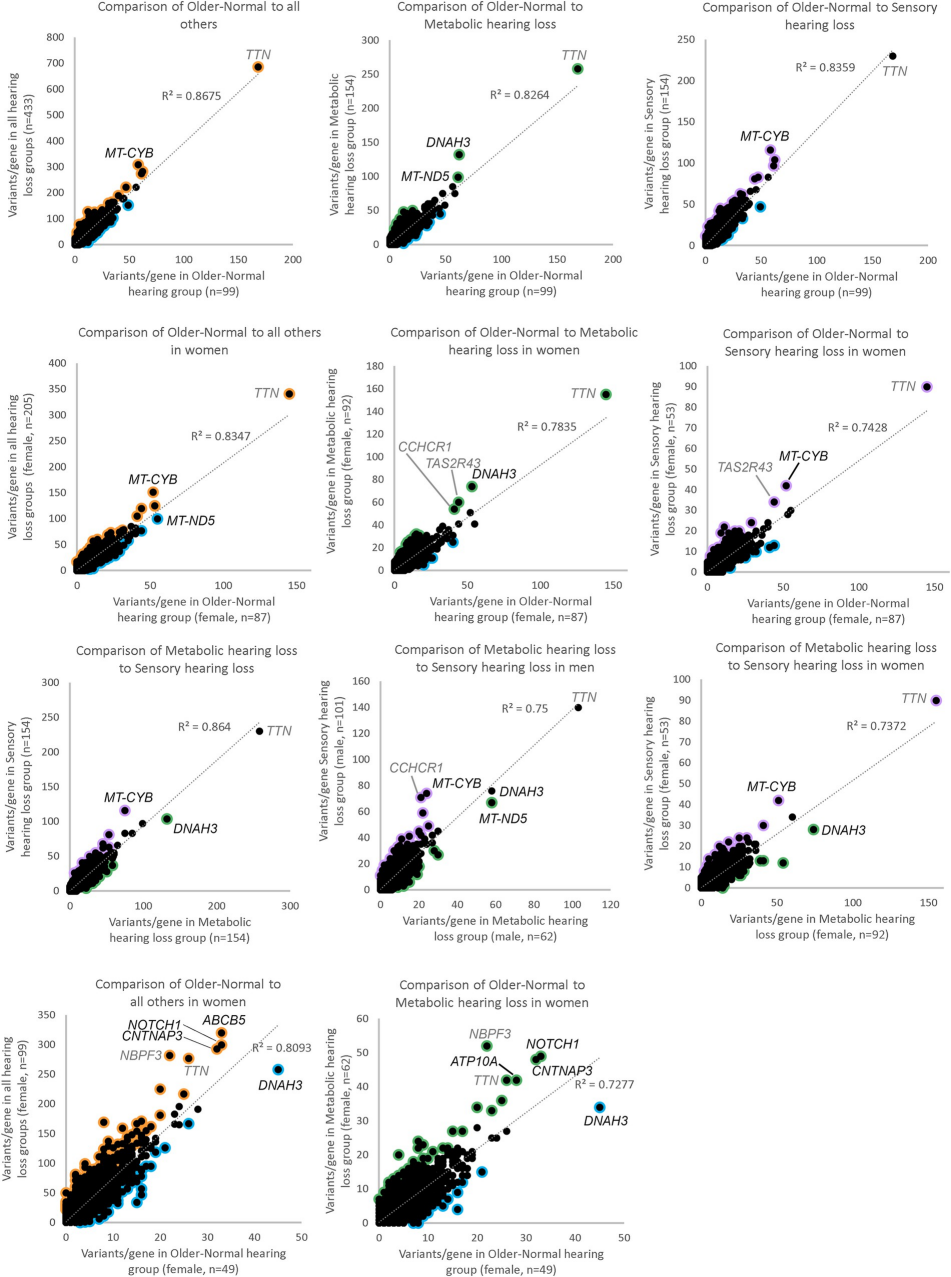
Comparison of variant load per gene between different classifications. Each point represents a gene. Outliers (S2 and S4 Tables) are marked in orange (for higher load in participants not classified as Older-Normal), blue (for higher load in participants classified as having Older-Normal hearing), purple (for higher load in participants classified as having Sensory hearing loss) or green (for higher load in participants classified as having Metabolic hearing loss). A-I show comparisons in the MUSC cohort; A,B,C,G show all participants, D,E,F,I show female participants and H shows male participants. A,D show a comparison of variant load in people in the Older-Normal category to all others in the cohort, B,E show a comparison of variant load in people in the Older-Normal category to people in the Metabolic category, C,F show a comparison of variant load in people in the Older-Normal category to people in the Sensory category, and G,H,I show a comparison of variant load in people in the Metabolic category to people in the Sensory category. J, K show comparison of variant load in the TwinsUK cohort (which is all female); J shows a comparison of variant load in people in the Older-Normal category to all others in the cohort and K shows a comparison of variant load in people in the Older-Normal category to people in the Metabolic category. Genes with a lot of variants in, at the top right of each plot, are labelled; in some cases these are highly variable genes (*TTN*, *CCHCR1*, *NBPF3*, *TAS2R43*, shown in grey). Having more variants overall does not necessarily make them of interest. What matters is whether a gene has many more or many fewer variants than expected, and that can be just as true of a gene with comparatively few variants overall (at the bottom left of the plot) as of one with many, such as *TTN*, which is at the top right of most plots.

To investigate variant load in hearing loss, and in and between the specific phenotypes, the number of variants per gene in participants belonging to one group (eg Older-Normal) were compared to the number of variants in the same gene in participants belonging to another group (eg Metabolic). The participants were also compared segregated by sex, because the genetic contribution to adult-onset hearing loss differs by sex [1]; however, because there were only 12 men classified as having Older-Normal hearing, comparisons which required that group were not carried out, resulting in 9 comparisons from the MUSC cohort (Fig 2A–2I; Table 3). Two lists of outlier genes were obtained from each comparison; one with an exceptionally high variant load in the first group and one with an exceptionally high variant load in the second group (S2 Table; raw variant counts are in S3 Table). The definition of a high variant load in each gene is not based on overall variant count, but on what is expected based on the other comparison group, so a large gene with many variants (such as *TTN*) will not necessarily be an outlier (eg Fig 2C). At the other extreme, a gene which does not have many variants overall may still be an outlier if it has more than expected in one group, and so there are outlier genes in the bottom left quadrant of all the plots in Fig 2 (marked by colour around the plotted point).

Several genes with lots of variants are labelled in the panels of Fig 2, some of which are outliers with exceptionally high variant loads in one of the comparison groups. For example, *DNAH3* is an outlier in metabolic hearing loss in the MUSC cohort (panels B, E, G, I) and was also previously reported as associated with hearing loss [17], but in contrast it is an outlier in normal hearing in the Twins UK cohort (panels J, K). *MT-CYB* is an outlier in the sensory hearing loss groups in the MUSC cohort (panels A, B, C, D, F, G, H, I) but was an outlier in the normal hearing group of the TwinsUK cohort (S4 Table), so the association was not replicated.

**Table 3:** Outlier gene list counts.

Cohort	Participants	Comparison	Variant load in metabolic hearing loss			Variant load in Sensory hearing loss		
			**Genes**	**Deafness genes**	**Variable genes**	**Genes**	**Deafness genes**	**Variable genes**
MUSC	All	Older-Normal vs all others	31	1 (adj.p = 1)	2 (adj.p = 0.40)	96	7 (adj.p = 0.39)	16 (adj.p = 2.95x10^-6^)*
MUSC	All	Older-Normal vs Metabolic	10	0 (adj.p = 1)	0 (adj.p = 1)	40	8 (adj.p = 0.0054)*	6 (adj.p = 0.0068)*
MUSC	All	Older-Normal vs Sensory	7	0 (adj.p = 1)	0 (adj.p = 1)	58	4 (adj.p = 0.57)	10 (adj.p = 0.00018)*
MUSC	Female	Older-Normal vs all others	35	1 (adj.p = 1)	2 (adj.p = 0.44)	107	9 (adj.p = 0.28)	18 (adj.p = 9.32x10^-7^)*
MUSC	Female	Older-Normal vs Metabolic	4	0 (adj.p = 1)	1 (adj.p = 0.23)	16	2 (adj.p = 0.40)	5 (adj.p = 0.00070)*
MUSC	Female	Older-Normal vs Sensory	6	0 (adj.p = 1)	2 (adj.p = 0.036)*	51	5 (adj.p = 0.33)	7 (adj.p = 0.0060)*
TwinsUK	Female	Older-Normal vs all others	147	5 (p = 0.78)	10 (p = 0.046)*	269	18 (p = 0.048)*	32 (p = 4.27x10^-9^)*
TwinsUK	Female	Older-Normal vs Metabolic	23	1 (p = 0.64)	1 (p = 0.58)	55	3 (p = 0.43)	5 (p = 0.052)
			**Variant load in metabolic hearing loss**			**Variant load in Sensory hearing loss**		
			**Genes**	**Deafness genes**	**Variable genes**	**Genes**	**Deafness genes**	**Variable genes**
MUSC	All	Metabolic vs Sensory	13	2 (adj.p = 0.40)	1 (adj.p = 0.43)	34	0 (adj.p = 1)	6 (adj.p = 0.0038)*
MUSC	Male	Metabolic vs Sensory	23	5 (adj.p = 0.026)*	2 (adj.p = 0.28)	89	5 (adj.p = 0.62)	15 (adj.p = 3.96x10^-6^)*
MUSC	Female	Metabolic vs Sensory	10	1 (adj.p = 0.65)	2 (adj.p = 0.088)	40	1 (adj.p = 1)	3 (adj.p = 0.27)

The number of genes, known deafness genes and highly variable genes in the high variant load lists from the outlier regression analyses comparing different phenotypes in the MUSC and TwinsUK cohorts. Asterisks indicate significant enrichment of deafness or highly variable genes in the high variant load list. Genes and variant counts are listed in S2, S3, S4 and S5 Tables.

**Fig 3:**
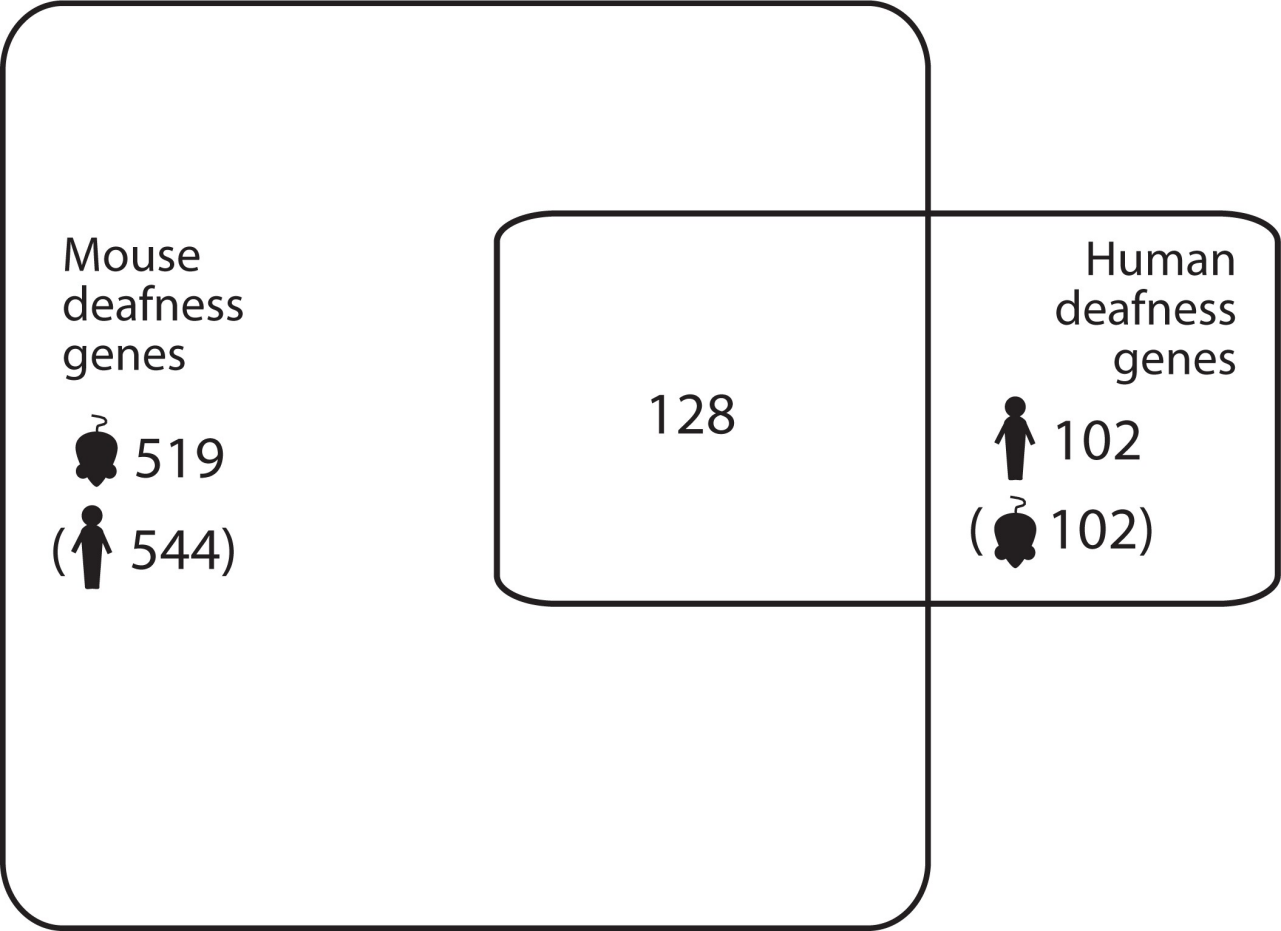
Numbers of known deafness genes in humans and mice. Brackets indicate orthologues (e.g. there are 544 human orthologues of the 519 mouse deafness genes).

To investigate these gene lists, findings were compared to a list of 774 genes which are known to underlie hearing loss in humans and/or mice (S6 Table; this includes 544 human orthologues of the 519 deafness genes known only from mouse studies (Fig 3)). These are good candidates for adult-onset hearing loss, and we suggest that enrichment in these genes supports the relevance to hearing loss. Only two lists showed a significant enrichment for hearing genes; the list of genes with high variant load in Metabolic hearing loss (male and female participants together, comparing Older-Normal to Metabolic hearing loss), and the list of genes with high variant load in Metabolic hearing loss (male participants, comparing Metabolic hearing loss to Sensory hearing loss) (Table 3). The gene lists were also tested for enrichment in 1213 highly variable genes, which are genes frequently reported to carry variants in multiple exome sequencing projects [1]. A significant enrichment of highly variable genes was found in multiple gene lists (Table 3), suggesting that some of the genes included are present for reasons unrelated to hearing. The outlier lists were combined to obtain a final candidate list of 291 genes, 21 of which were known deafness genes and 37 of which were highly variable genes (S2 Table). 107 of these genes were also identified in our previous study of self-reported hearing difficulty in the UK BioBank cohort [1], 12 of which were known deafness genes (*PKHD1L1*, *ELMO3*, *CDH23*, *UBE3B*, *ADGRV1*, *COL9A3*, *NAV2*, *DMD*, *AFAP1L2*, *MPDZ*, *LOXHD1*, and *CELSR1*).

To prioritise the list of candidate genes, a third list of outlier genes was obtained from the TwinsUK cohort. In this case there were not sufficient participants classified as having Sensory hearing loss, and so only two comparisons were carried out (Fig 2J and 2K; Table 3), resulting in a final candidate list of 435 genes, including 25 known deafness genes and 43 highly variable genes (S4 Table, raw variant counts in S5 Table).

**Fig 4:**
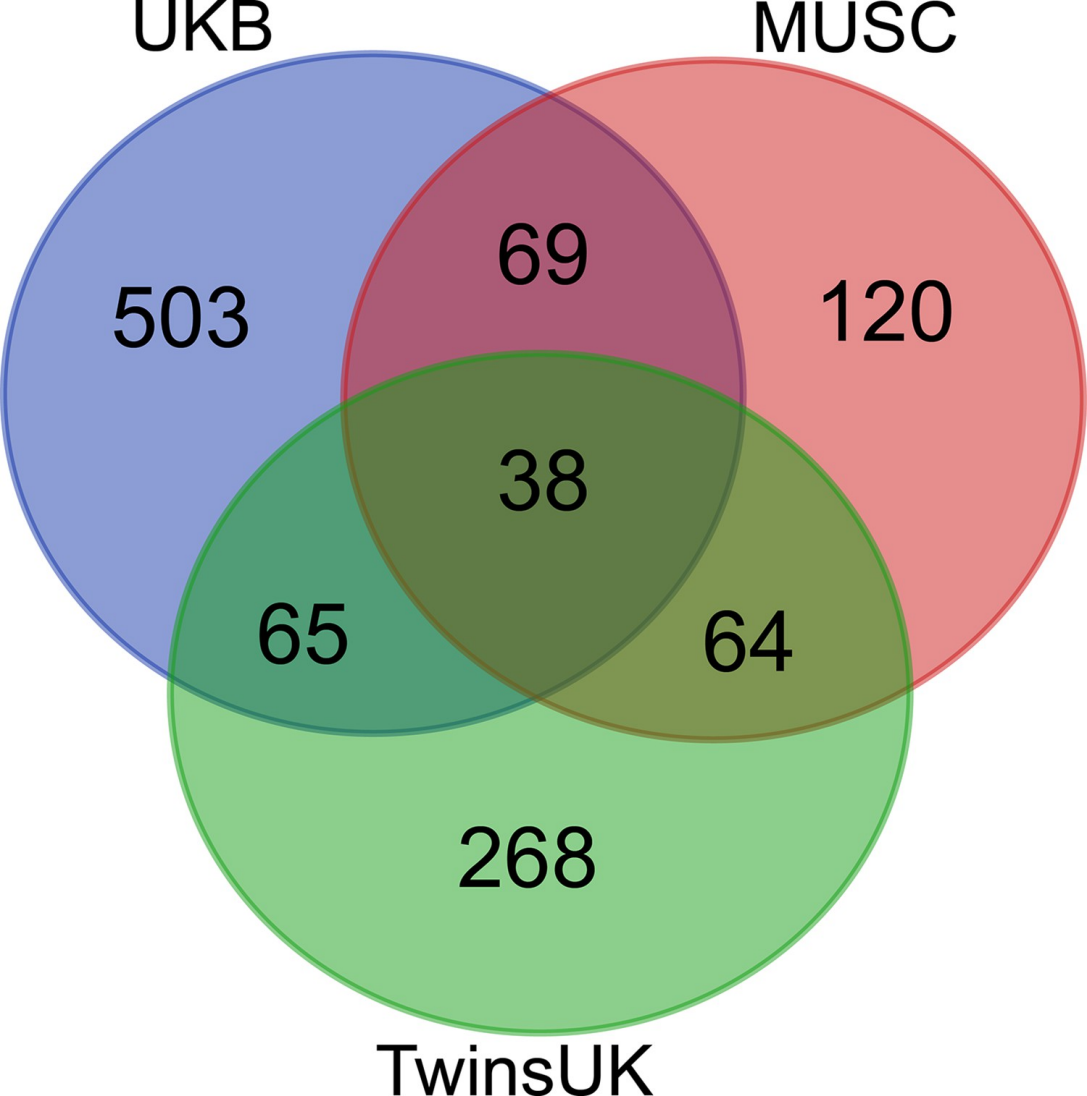
Overlap in gene lists from the two cohorts described in this study and the UK Biobank. Genes are listed in S2 and S4 Tables, and the UK Biobank gene list is from our previous study [1].

Thirty-eight genes were common to all three analyses (Fig 4, Table 4), one of which was a known deafness gene (*PKHD1L1*) (Fig 4). For further prioritisation, we investigated the expression of mouse orthologues in the inner ear using publicly available single cell RNAseq data from the gEAR expression resource [18]. Thirty-two of the 38 genes had good quality mouse orthologues, and of these, eleven genes had no expression reported in the chosen ages and cell types, and a further eleven genes were expressed at low levels (up to and including the expression level of *Hprt1*, to which all expression was normalised). The remaining 10 genes were strongly expressed in at least one cell type and age (S3 Fig). Based on this analysis, among the most interesting novel candidate genes were *FKBP2* and *SYNE2*, which have strong expression in multiple cochlear and lateral wall cell types, and *ABCB8*, which shows similar expression to the hair cell marker *Myo7a* (S3 Fig).

**Table 4:** The 38 genes common to outlier analyses on 3 different cohorts.

Gene ID	Gene name	MUSC outlier list	TwinsUK outlier list	UKB outlier list
ENSG00000197150	*ABCB8*	HL	Metabolic HL	Normal hearing
ENSG00000108846	*ABCC3*	HL	Normal hearing	Normal hearing
ENSG00000060971	*ACAA1*	HL, Sensory HL	Not normal hearing	Hearing difficulty
ENSG00000122359	*ANXA11*	HL, Sensory HL	Not normal hearing	Hearing difficulty
ENSG00000100628	*ASB2*	HL, Sensory HL	Not normal hearing	Normal hearing
ENSG00000064270	*ATP2C2*	HL	Not normal hearing	Hearing difficulty
ENSG00000070748	*CHAT*	Sensory HL	Normal hearing	Normal hearing
ENSG00000070371	*CLTCL1*	Sensory HL	Not normal hearing	Hearing difficulty
ENSG00000198077	*CYP2A7*	Metabolic HL	Not normal hearing	Hearing difficulty
ENSG00000187630	*DHRS4L2*	Normal hearing	Not normal hearing	Normal hearing
ENSG00000114841	*DNAH1*	HL, Metabolic HL, Sensory HL	Not normal hearing	Normal hearing
ENSG00000158486	*DNAH3*	HL, Metabolic HL, Sensory HL	Normal hearing	Normal hearing
ENSG00000163687	*DNASE1L3*	Sensory HL	Normal hearing	Normal hearing
ENSG00000256061	*DYX1C1*	Normal hearing	Not normal hearing, Metabolic HL	Hearing difficulty
ENSG00000049540	*ELN*	Metabolic HL	Normal hearing	Hearing difficulty
ENSG00000173486	*FKBP2*	HL	Not normal hearing	Hearing difficulty
ENSG00000169224	*GCSAML*	Normal hearing, Sensory HL	Not normal hearing	Hearing difficulty, Normal hearing
ENSG00000107521	*HPS1*	Metabolic HL	Normal hearing	Normal hearing
ENSG00000056736	*IL17RB*	HL	Not normal hearing	Hearing difficulty
ENSG00000132849	*INADL*	HL	Not normal hearing	Hearing difficulty, Normal hearing
ENSG00000198399	*ITSN2*	HL, Sensory HL	Not normal hearing, Metabolic HL	Hearing difficulty
ENSG00000131738	*KRT33B*	Sensory HL	Not normal hearing	Hearing difficulty
ENSG00000126790	*L3HYPDH*	Sensory HL	Not normal hearing	Hearing difficulty
ENSG00000286264	*LOC114841035*	HL	Not normal hearing	Hearing difficulty
ENSG00000080823	*MOK*	Sensory HL	Not normal hearing, Metabolic HL	Normal hearing
ENSG00000078814	*MYH7B*	HL	Not normal hearing, Metabolic HL	Hearing difficulty
ENSG00000160194	*NDUFV3*	HL, Sensory HL	Normal hearing	Hearing difficulty
ENSG00000087303	*NID2*	Sensory HL	Not normal hearing, Metabolic HL	Normal hearing
ENSG00000205038	*PKHD1L1*	HL, Metabolic HL	Not normal hearing	Normal hearing
ENSG00000051341	*POLQ*	Normal hearing	Not normal hearing	Hearing difficulty
ENSG00000111344	*RASAL1*	HL	Not normal hearing	Hearing difficulty
ENSG00000243978	*RGAG1*	Sensory HL	Not normal hearing	Normal hearing
ENSG00000198870	*STKLD1*	Sensory HL	Not normal hearing	Hearing difficulty
ENSG00000054654	*SYNE2*	HL, Sensory HL	Not normal hearing, Metabolic HL	Normal hearing
ENSG00000138162	*TACC2*	HL, Sensory HL	Not normal hearing, Metabolic HL	Hearing difficulty, Normal hearing
ENSG00000155657	*TTN*	HL, Metabolic HL, Sensory HL	Not normal hearing, Metabolic HL	Hearing difficulty
ENSG00000129003	*VPS13C*	HL, Sensory HL	Metabolic HL	Hearing difficulty
ENSG00000146530	*VWDE*	HL, Metabolic HL, Sensory HL	Normal hearing	Normal hearing

Genes are listed along with the phenotype(s) for which they were outliers in each of the three cohorts. For a full description of the phenotype and sex groupings, see S3 and S5 Tables and [1]. HL = hearing loss.

In order to investigate genes associated with specific phenotypes, we also plotted the expression of genes identified only in the phenotype-specific analyses. There were 18 genes linked only to Metabolic hearing loss (including four deafness genes: *DMD*, *DUOX2*, *CELSR1* and *ELMO3*) and 54 genes linked only to Sensory hearing loss (including four deafness genes: *ARHGAP21*, *LMO7*, *UBE3B* and *ADGRV1*) (S2 and S4 Tables). After removing genes without a good quality mouse orthologue and with low or no expression in the chosen inner ear datasets, we plotted the expression of 12 Metabolic-linked genes and 17 Sensory-linked genes (S4 Fig). The four Metabolic-linked genes most strongly expressed in the lateral wall are *MT-CO1*, *TLN2*, *DPP4* and *CHMP4C*, and are also expressed in several organ of Corti cell types (S4 Fig). The Sensory-linked genes most strongly expressed in the organ of Corti are *MADD*, *UBE3B* and *LMO7*, but they have low or no expression in the lateral wall (S4 Fig). We tested the protein expression of Madd in the inner ear of wildtype mice at ages from embryonic day (E)14.5 to postnatal day (P)4, and found that there was no protein detected at E14.5, but expression in inner and outer hair cells was visible at E16.5 and increased in intensity up to P4 (S5 Fig), which correlates with the scRNAseq data from the gEAR (S4 Fig).

### Threshold difference detection

**Fig 5:**
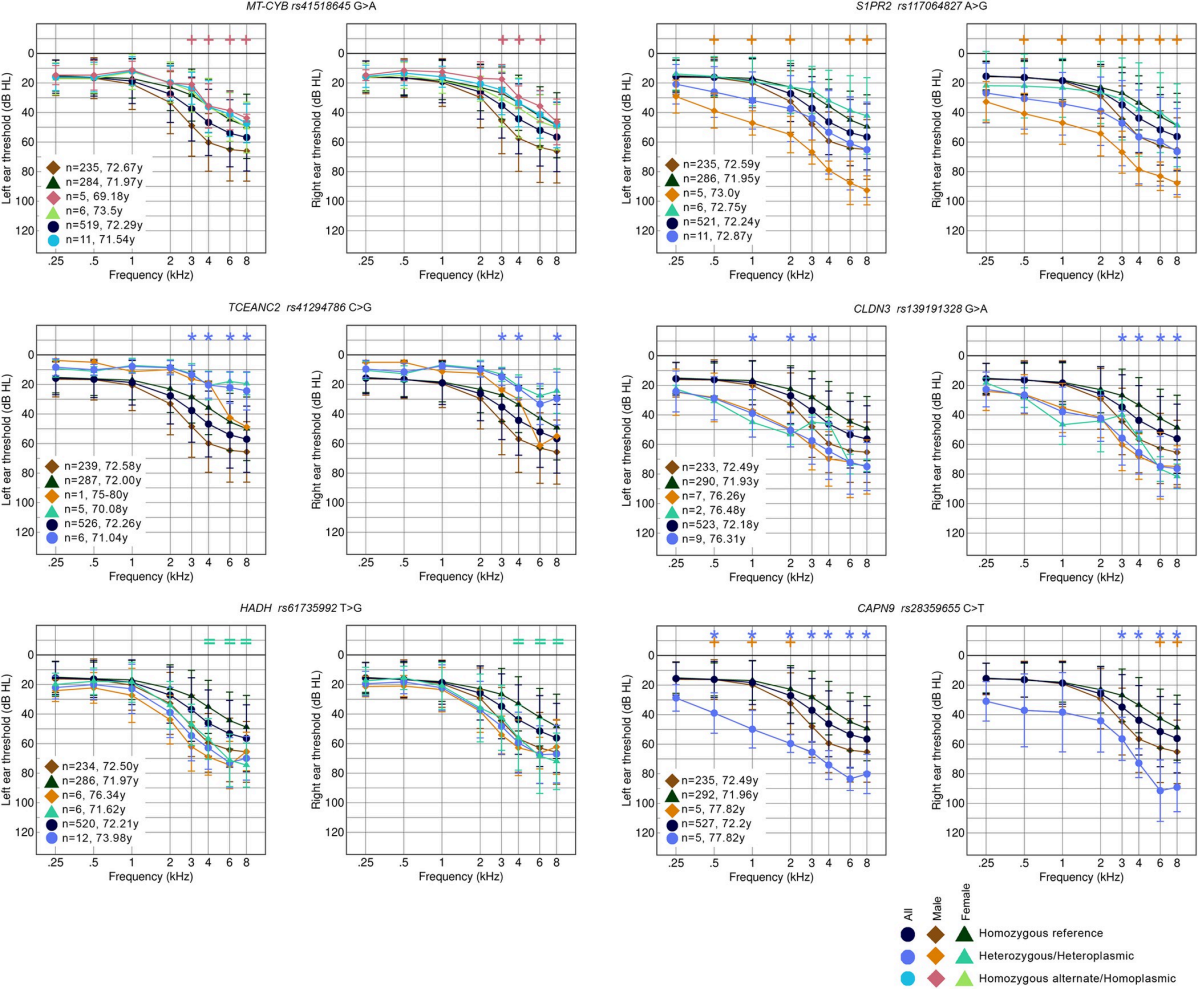
Average audiograms from the MUSC cohort plotted in groups by sex and genotype. Six different variants were chosen as examples from the full list of 41; see S6 Fig. Two audiograms are shown for each variant; the thresholds from the left ear are shown on the left, and those from the right ear on the right. Numbers and average ages of each group are listed on the graph. The symbols at the top of each graph mark which groups passed the criteria for each stimulus frequency compared to the relevant reference group (+ for male, = for female, and * for all participants). Carriers of the *MT-CYB* and *TCEANC2* variants have better thresholds than non-carriers, and carriers of the *MMS19*, *S1PR2*, *CLDN3* and *CAPN9* variants have worse thresholds than non-carriers. The variants in *S1PR2* and *HADH* are linked to worse thresholds only in male and female carriers respectively, and there are no female carriers of the *CAPN9* variant so it is unknown whether they would be similarly affected to male carriers.

We compared the thresholds of carriers of each individual variant to those of non-carriers in order to assess the contribution of each variant to threshold differences. Forty of the 29,807 high impact variants in the nuclear exome passed the filters and permutation testing. In two cases (*KIRREL1* and *CCDC171*), both the non-segregated alternate allele group and one of the sex-segregated groups exhibited a significant difference in thresholds. In the remaining 38 cases, only one group exhibited a significant threshold difference. One mitochondrial variant (rs41518645, in *MT-CYB*) also was found to pass the filter and permutation tests, and was associated with better thresholds in male participants (Fig 5). There were no instances of multiple variants being identified in the same gene, and only two were in known deafness genes, *S1PR2* and *PIEZO1* (Table 5, Figs 5 and S6). Sixteen of the 41 variants were associated with better thresholds than the sex-matched reference group (eg *TCEANC2*, Fig 5), and 25 with worse thresholds than the reference group (eg *CLDN3*, Fig 5). Fifteen variants exhibited a significant difference in thresholds in only one sex (eg *S1PR2*, *HADH*, Fig 5), not including those instances where there were too few carriers of the opposite sex to determine if their thresholds were similarly affected, eg *CAPN9* (Fig 5, Table 5; all audiograms are shown in S6 Fig).

**Table 5:** Variants identified by threshold differences in carriers.

a. Variants identified in MUSC cohort									
**Variant details**	**Protein change and Ensembl transcript ID**	**MAF (gnomAD NFE)**	**Gene**	**Number of variants tested in this gene**	**Effect on thresholds**	**Number of similar permu-tations (out of 20,000)**	**Adj. p-value**	**Male/ female difference?**	**Carriers in the TwinsUK cohort?**
7:73769649 G>A *rs139191328*	Pro134Leu; ENST00000395145	9.67x10^-3^	*CLDN3*	3	Worse	0	0		No
19:10224049 A>G *rs117064827*	Val286Ala; ENST00000646641	8.91x10^-3^	*S1PR2*	2	Worse	0	0	Only seen in men	Not comparable
9:15874587 T>G *rs149814894*	Leu1175Arg; ENST00000380701	7.67x10^-3^	*CCDC171*	7	Worse	1 (all), 3 (male)	5.5x10^-4^ (all), 9.4x10^-4^ (male)	Only seen in men	Not comparable
1:230772053 C>T *rs28359655*	Arg277Trp; ENST00000271971	2.80x10^-3^	*CAPN9*	5	Worse	1 (male), 2 (all)	7.3x10^-4^ (male), 8.8x10^-4^ (all)	No female carriers	Not comparable
11:57301020 G>A *rs139208640*	Arg1665Cys; ENST00000358252	6.27x10^-3^	*TNKS1BP1*	5	Worse	2	7.33x10^-4^		Two carriers, whose thresholds were variable between left and right ears
6:166382716 C>A *rs550593206*	Intronic splice variant	6.67x10^-3^	*MPC1*	1	Worse	4	8.80x10^-4^	Only seen in women	No
17:58195140 C>A *rs35617692*	Cys257*; ENST00000225371	9.01x10^-3^	*EPX*	8	Worse	4	9.78x10^-4^	Only seen in women	Two carriers, whose thresholds were variable between left and right ears
6:77463033 A>C *rs130060*	Phe124Cys; ENST00000369947	1.25x10^-2^	*HTR1B*	1	Worse	5	1.00x10^-3^		Seven carriers, whose thresholds were variable but overall similar to those homozygous for the reference allele
14:54531033 C>T *rs34839928*	Arg185Trp; ENST00000216420	6.88x10^-3^	*CGRRF1*	4	Worse	4	1.10x10^-3^	Only seen in women	Two carriers, whose thresholds were similar to those homozygous for the reference allele
19:13882951 G>A *rs77270337*	Arg620Trp; ENST00000586783	7.02x10^-3^	*C19orf57 (BRME1)*	1	Better	15	2.36x10^-3^	Only seen in men	Not comparable
1:158093761 C>T *rs139995772*	Ser573Leu; ENST00000359209	9.34x10^-4^	*KIRREL1*	2	Better	14 (female), 153 (all)	2.4x10^-3^ (female), 0.011 (all)	One carrier, with worse hearing	1:158093761
4:108014444 T>G *rs61735992*	Phe92Cys; ENST00000309522	1.33x10^-2^	*HADH*	1	Worse	13	2.38x10^-3^	Only seen in women	Five carriers with similarly affected audiograms (significant in TwinsUK)
14:45242137 G>C *rs34168608*	Pro347Arg; ENST00000310806	1.22x10^-2^	*MIS18BP1*	3	Worse	19	2.79x10^-3^	Only seen in men	Not comparable
12:122340851 C>T *rs61954403*	Glu785Lys; ENST00000620786	1.52x10^-2^	*CLIP1*	5	Worse	26	3.36x10^-3^	Only seen in women	Five carriers, whose thresholds were similar to those homozygous for the reference allele
16:70156611 G>A *rs117263218*	Arg791Gln; ENST00000288050	5.48x10^-3^	*PDPR*	13	Worse	25	3.44x10^-3^	Only seen in women	Two carriers, with better hearing
17:40487189 C>T *rs144692706*	Gly379Ser; ENST00000254051	1.03x10^-2^	*TNS4*	7	Worse	29	3.54x10^-3^	Only seen in men	Not comparable
2:128268653 G>T *rs3958533*	Arg249Ser; ENST00000259241	9.61x10^-3^	*HS6ST1*	2	Worse	31	3.59x10^-3^	Only seen in men	No
9:105635214 A>G *rs41313301*	Asn446Asp; ENST00000357998	1.18x10^-2^	*FKTN*	4	Better	33	3.63x10^-3^	Only seen in men	Not comparable
11:40116285 T>G *rs144974170*	Asn3Thr; ENST00000528697	3.53x10^-3^	*LRRC4C*	2	Better	38	3.98x10^-3^		One carrier, with worse hearing
MT:15257 G>A *rs41518645*	Asp171Asn; ENST00000361789	2.41x10^-2^	*MT-CYB*	36	Better	46	4.40x10^-3^	Only seen in men	No
14:21000977 T>A *rs72684072*	Ser110Thr; ENST00000298681	3.27x10^-3^	*SLC39A2*	3	Worse	49	4.49x10^-3^		Four carriers, whose thresholds were similar to those homozygous for the reference allele
18:70197682 T>G *rs12956068*	Asp212Ala; ENST00000640769	5.40x10^-3^	*RTTN*	6	Worse	45	4.50x10^-3^		No
1:54096337 C>G *rs41294786*	Ser164Cys; ENST00000234827	2.87x10^-3^	*TCEANC2*	2	Better	58	5.10x10^-3^		No
9:108862962 G>A *rs41278347*	Gly214Ser; ENST00000333999	8.20x10^-3^	*ACTL7A*	2	Worse	71	6.01x10^-3^		Two carriers, with better hearing
10:97458699 C>T *rs36023427*	Gly1029Asp; ENST00000438925	5.33x10^-3^	*MMS19*	6	Worse	74	6.03x10^-3^	Only seen in men	No
20:54171651 G>A *rs35873579*	Arg157Trp; ENST00000216862	3.27x10^-3^	*CYP24A1*	7	Better	90	7.07x10^-3^		One carrier, with worse hearing
14:60027961 C>T *rs368587449*	Arg1261*; ENST00000570145	4.47x10^-3^	*LRRC9*	11	Better	108	8.19x10^-3^		No
10:112445316 C>T *rs34350728*	Asp41Asn; ENST00000369405	1.40x10^-2^	*ZDHHC6*	2	Better	152	1.11x10^-2^		Three carriers, with similar thresholds
20:3691399 C>T *rs143489222*	Cys1511Tyr; ENST00000344754	5.00x10^-3^	*SIGLEC1*	4	Better	172	1.18x10^-2^		No
16:15036070 G>A *rs148061029*	Ser721Asn; ENST00000396410	3.73x10^-3^	*PDXDC1*	7	Better	200	1.26x10^-2^		No
7:107975743 G>A *rs28750165*	Pro379Ser; ENST00000222399	1.73x10^-3^	*LAMB1*	8	Better	190	1.27x10^-2^		No
9:420579 A>G *rs116920018*	Tyr1340Cys; ENST00000432829	3.33x10^-3^	*DOCK8*	6	Worse	196	1.27x10^-2^		Four carriers, whose thresholds were similar to those homozygous for the reference allele
16:88721827 G>A *rs139051768*	Thr1732Met; ENST00000301015	8.03x10^-3^	*PIEZO1*	16	Worse	264	1.57x10^-2^		One heterozygous and two homozygous carriers, whose thresholds were similar to those homozygous for the reference allele
1:1485777 G>A *rs1622213*	Intronic splice variant	7.11x10^-3^	*ATAD3B*	9	Worse	263	1.61x10^-2^		Two carriers, whose thresholds were similar to those homozygous for the reference allele
3:151190791 G>T *rs34501514*	Asp610Tyr; ENST00000687756	2.13x10^-3^	*MED12L*	3	Better	290	1.68x10^-2^		One carrier, with similar thresholds
3:39107084 T>A *rs575892658*	Glu31Val; ENST00000437458	6.20x10^-3^	*GORASP1*	6	Better	327	1.84x10^-2^		No
10:132885887 T>C *rs150871636*	Asp1126Gly; ENST00000368586	8.49x10^-3^	*CFAP46*	11	Worse	337	1.85x10^-2^		No
7:1547804 G>A *rs61747419*	Ser317Phe; ENST00000297477	1.06x10^-2^	*TMEM184A*	3	Worse	462	2.48x10^-2^		No
16:2181024 T>C *rs183093419*	Lys782Glu; ENST00000343516	5.37x10^-3^	*CASKIN1*	8	Better	634	3.24x10^-2^		No
1:32695311 G>A *rs41265855*	Arg263Cys; ENST00000409190	3.54x10^-3^	*SYNC*	3	Better	634	3.32x10^-2^		No
20:62351710 G>A *rs78026347*	Pro317Leu; ENST00000252999	3.34x10^-3^	*LAMA5*	24	Worse	849	4.25x10^-2^		Two carriers, with better hearing
**b. Variants identified in TwinsUK cohort**									
**Variant details**	**Protein change and Ensembl transcript ID**		**Gene**		**Effect on thresholds**	**Number of similar permutations (out of 20,000)**	**p-value**	**Male/ female difference?**	**Carriers in the MUSC cohort?**
4:108014444 T>G *rs61735992*	Phe92Cys; ENST00000309522	1.33x10^-2^	*HADH*	3	Worse	38	1.90x10^-3^	N/A	Similar thresholds in female carriers (6 male carriers, 6 female carriers in MUSC, significant in female carriers)
2:166409937 T>C *rs62622799*	Splice acceptor variant	1.80x10^-2^	*SCN7A*	3	Worse	40	2.00x10^-3^	N/A	22 carriers with similar thresholds (not significant in MUSC)
6:83398481 T>A *rs151111787*	Glu83Val; ENST00000369705	1.81x10^-2^	*ME1*	4	Better	42	2.05x10^-3^	N/A	Fifteen heterozygous carriers, variable thresholds but average is similar to reference
1:19285998 C>T *rs148340817*	Intronic splice variant	2.77x10^-2^	*AKR7A3*	3	Worse	152	7.60x10^-3^	N/A	One homozygous and 34 heterozygous carriers, variable thresholds but average is similar to reference

Details of variants identified by associated threshold differences in the MUSC (a) and TwinsUK (b) cohorts. Variants are ordered by adjusted p-value. Minor allele frequencies are shown for the Non-Finnish European population, from the gnomAD database v2 [19]. In the case of the mitochondrial variant, the homoplasmic allele frequency is shown, from gnomAD v3.1 [20].

To further investigate the contribution of these 41 variants to threshold differences, carriers of each variant in the TwinsUK cohort were identified, and their audiograms plotted compared to homozygous carriers of the reference allele. Sixteen of the 41 variants were not found in any of the TwinsUK participants (Table 5), and for a further seven variants, the threshold difference was only seen in male MUSC carriers, not in female participants, so a comparison was not possible with the all-female TwinsUK cohort (Table 5). However, five carriers from the TwinsUK cohort were found to have similar audiograms to those in the MUSC cohort for the variant in *HADH*, and this was also found independently when the same filter and permutation testing was carried out on the TwinsUK cohort. Carriers of variants in *MED12L* (n = 2), and *ZDHHC6* (n = 3) also had a similar average threshold shape to that seen in the MUSC cohort carriers (Table 5, S6 Fig), supporting the suggestion of a potential role for these variants in contributing to the hearing loss seen in carriers. We examined the MUSC carriers of the variants in *HADH*, *MED12L* and *ZDHHC6* to check for any variants in 50 known dominant deafness genes (https://hereditaryhearingloss.org, accessed March 2023 [21]) but did not find any dominant gene consistently affected within each group.

From the TwinsUK cohort alone, only four variants passed the filters and permutation testing, one of which was the variant in *HADH*, also identified in the MUSC cohort. The other three genes were *AKR7A3*, *SCN7A* and *ME1* (S6 Fig). There were many carriers of each of these three variants in the MUSC cohort, but for *ME1* and *AKR7A3*, the average thresholds of carriers did not show any obvious difference to non-carriers, suggesting that if these variants do contribute to hearing loss, the impact is not reflected in audiogram shape (S6 Fig). MUSC carriers of the variant in *SCN7A* (n = 22; 9 female, 13 male) had, on average, slightly worse thresholds than homozygous carriers of the reference allele, resembling the thresholds of carriers in the TwinsUK cohort, but the difference was not significant (S6 Fig).

## Discussion

From the outlier analysis, we identified 38 candidate genes that may contribute to overall hearing status (including one deafness gene, *PKHD1L1*) (Table 4), 18 genes linked to Metabolic hearing loss alone (including four deafness genes, *DMD*, *DUOX2*, *CELSR1* and *ELMO3*) (S3 and S5 Tables), and 54 genes linked to Sensory hearing loss alone (including four deafness genes, *ARHGAP21*, *LMO7*, *UBE3B* and *ADGRV1*) (S3 Table). The threshold analysis revealed 41 candidate genes including two known deafness genes (*S1PR2* and *PIEZO1*) (Table 5).

### Known deafness genes from the candidate gene lists

Our candidate gene lists include 11 deafness genes; *S1PR2*, *PIEZO1*, *PKHD1L1*, *DMD*, *DUOX2*, *CELSR1*, *ELMO3*, *ARHGAP21*, *LMO7*, *UBE3B* and *ADGRV1*. Only 3 of these have been identified in humans; *ADGRV1*, which is an Usher syndrome type II gene [22], *DMD*, which has been associated with congenital hearing impairment as well as muscular dystrophy [23], and *S1PR2*, variants in which lead to congenital profound hearing impairment [24], although a point variant in *S1pr2* in mice results in early-onset progressive hearing loss [25]. These phenotypes are more severe than the late-onset progressive hearing loss in our human subject cohorts, which supports the theory that genes responsible for severe deafness may also be involved in milder forms of hearing loss.

*PIEZO1*, *PKHD1L1*, *DUOX2*, *CELSR1*, *ELMO3*, *ARHGAP21*, *LMO7* and *UBE3B* are all defined as deafness genes through work on the mouse orthologues. Of these, hearing loss caused by mutant alleles of *Arhgap21* and *Elmo3* have only been reported by the IMPC large-scale phenotyping screen at a single age of 14 weeks (www.mousephenotype.org [26, 27]); *Elmo3* homozygous mutants have raised thresholds at low frequencies (https://www.mousephenotype.org/data/genes/MGI:2679007) and *Arhgap21* heterozygous mutants exhibit variably raised thresholds across most frequencies tested (https://www.mousephenotype.org/data/genes/MGI:1918685). Mice with a disrupted *Ube3b* gene display mild hearing impairment at all frequencies at 3 months old, and this impairment was more severe when tested at 6 months old [28]. *Pkhd1l1* mutants show early-onset progressive hearing loss [29], while abolishing *Lmo7* expression in mice results in late-onset progressive hearing impairment [30]. All these are comparatively mild effects, but mice carrying a missense variant in *Duox2* have severely raised thresholds [31], and mice carrying variants in the planar cell polarity gene *Celsr1* exhibit vestibular defects and misoriented outer hair cells [32]. Finally, mice carrying a mutation in *Piezo1* have recently been reported to exhibit progressive hearing loss [33].

### Expression analysis of novel candidate genes

Interestingly, most of our newly associated genes have not previously been reported with hearing loss, suggesting that there are many more genes involved in hearing which remain to be identified. Our expression analysis results suggest some promising genes for further investigation, such as *SYNE2*, *FKBP2*, and *ABCB8* from the main analysis (Table 4), and *MADD*, *COG4* and *CHMP4C* from the phenotype-specific analysis (S3 and S5 Tables). SYNE2 forms part of the LINC (Linker of Nucleoskeleton and Cytoskeleton) Complex, which is part of the nuclear envelope and is essential for maintenance of normal hearing [34]. *FKBP2* encodes FKBP13, a luminal endoplasmic reticulum (ER) protein which is upregulated in response to cellular stress such as heat shock, or the accumulation of unfolded protein precursors in the ER [35,36], and *ABCB8* is a mitochondrial ABC transporter which plays a role in cellular viability and is protective against oxidative stress [37]; variants in either may contribute to the vulnerability of inner ear cells to damage and age-related deterioration. *MADD*, which was associated with Sensory hearing loss and has an expression pattern resembling that of *Myo7a* (S4 Fig and S3 Table), is an activator of the Rab3 small GTP-binding protein family, and has been shown to be critical for neurotransmitter release in neuromuscular junctions and in hippocampal neurons [38,39]; it also may play a role in inner ear synapses but that has yet to be determined. *COG4*, which was also associated with Sensory hearing loss (S3 Table), is strongly expressed in hair cells and Deiters cells (S4 Fig), and is known to be important for zebrafish inner ear development [40]. *CHMP4C*, which was associated with Metabolic hearing loss in the TwinsUK cohort (S5 Table), is expressed in the marginal and basal cells of the stria vascularis, as well as several cell types in the organ of Corti (S4 Fig). Previous whole exome sequencing and genome-wide association studies have also linked *CHMP4C* to hearing impairment, suggesting it is a good candidate for further study [1,6–8]. However, it should be noted that gene expression in a particular cell type is not a guarantee of a critical role in that cell type, and the absence of expression in inner ear cells at the times and stages studied does not preclude a gene from having a role in hearing. It may be needed at a later time in development, or elsewhere in the auditory pathway, or may only be needed only at very low quantities, making it difficult to detect by single cell RNAseq. Also, given the limited data available from expression studies, a role for the other candidate genes and variants in age-related hearing loss should not be discounted.

### Novel candidate genes from the threshold analyses

From our threshold analyses on both cohorts, we identified a variant in the gene *HADH* as a candidate associated with worse hearing, and variants in the genes *ZDHHC6* and *MED12L* were associated with better hearing (S6 Fig, Table 5). HADH (hydroxyacyl-Coenzyme A dehydrogenase) localises to the mitochondrial matrix where it plays a role in the beta-oxidation pathway, breaking down fatty acid molecules to generate acetyl-coA. Variants in other genes in the same pathway have been shown to result in mitochondrial dysfunction [41], suggesting a potential mechanism for *HADH* variants to affect hearing. ZDHHC6 is a palmitoyltransferase located in the ER, and defects in palmitoylation have been linked to hearing impairment [42]. MED12L is a subunit of the Mediator protein complex which is part of the basal transcriptional apparatus; post-natal deletion of the Med12 subunit of the same complex in mice results in rapid loss of basal cell organisation and disruption of the stria vascularis leading to hearing loss [43].

### The genetic contribution to hearing differences between sexes

The MUSC cohort has a slight excess of female participants over male, but the difference in classification of their hearing is marked, with too few male participants classified as “Older-Normal” to carry out a robust regression analysis on men alone using that category (Table 1, Fig 1). This difference has been previously described in multiple studies [11,44–49], with hearing in women tending to be better than in men and declining later in life, generally around the onset of menopause [11,50]. However, the average age of the participants in the MUSC cohort is over 60, suggesting that there is also a genetic contribution to the difference in hearing impairment between the sexes, as observed in our previous study [1].

The other clear difference in auditory phenotype between the sexes can be seen in the number of men classified as having Sensory hearing loss (101, 42% of male participants) compared to women (53, 18% of female participants) (Table 1). The proportions in the Metabolic hearing loss group are the inverse, although not so extreme (62 men, 26% of male participants, and 92 women, 32% of female participants). However, in the all-female TwinsUK cohort, there are only 7 participants classified as having Sensory hearing loss (5%, not including the twins who were removed; Table 1). Sensory hearing loss has been attributed to noise exposure, among other factors, and most of the men in the MUSC cohort had a positive noise exposure history (189, 79% of all male participants). However, the proportion of men reporting a history of noise exposure across the three classified groups was broadly similar (Older-Normal: 67%; Sensory: 79%; and Metabolic: 69%) (Table 1, Fig 1). The proportion of women in the MUSC cohort reporting a positive noise history in the different classifications was also very similar (Table 1, Fig 1). Self-reported noise history alone thus does not explain the excess of male participants classified as having Sensory hearing loss in the MUSC cohort. There may be a sex-specific genetic contribution to this observation, but more data are needed for further exploration. A more objective, quantifiable measure of noise exposure would also help in this, since noise history questionnaires can be an unreliable measure of an individual’s noise exposure.

**Fig 6:**
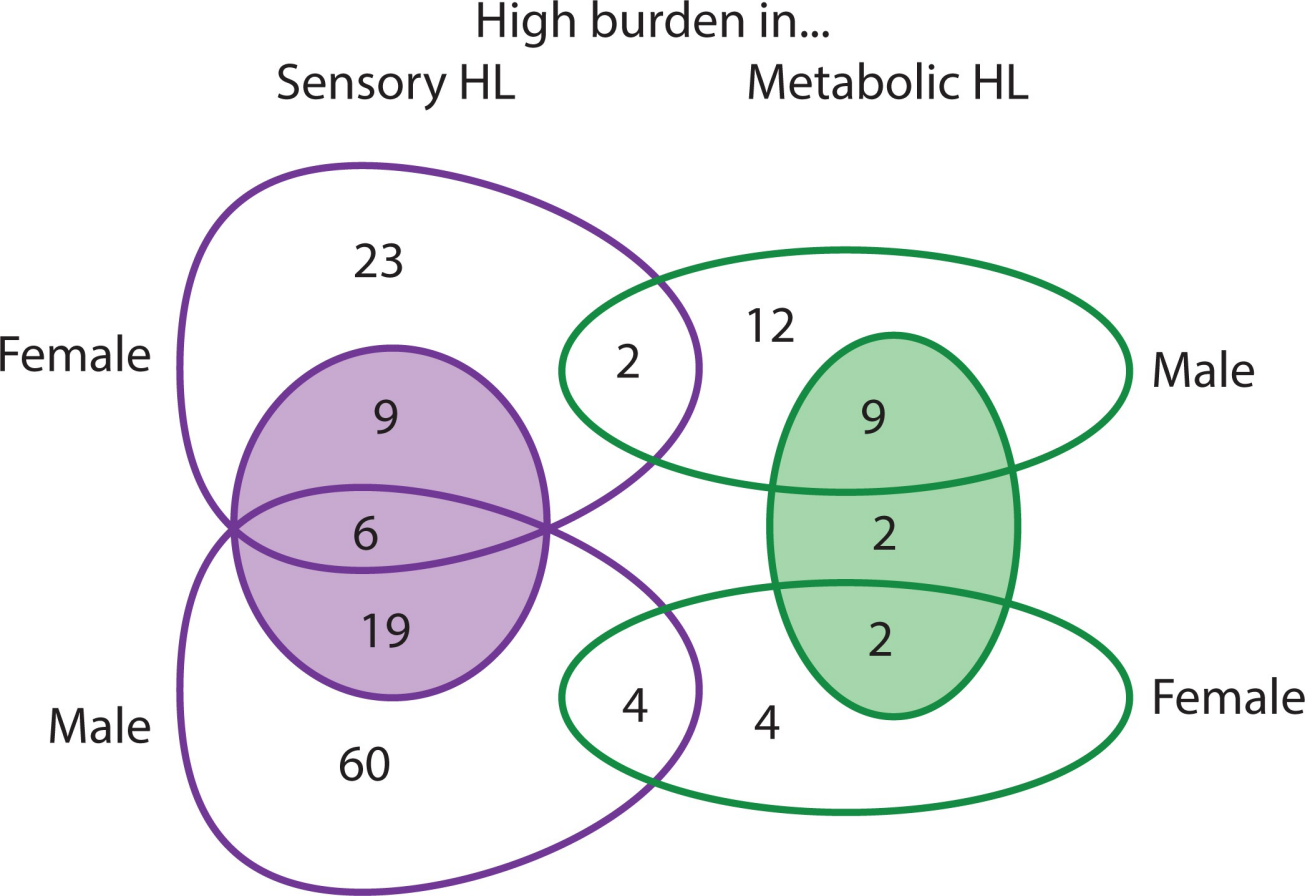
Not many genes are shared between male and female Metabolic and Sensory gene lists. The Venn diagram shows the overlap of genes identified as having a high variant load in Metabolic or Sensory hearing loss in all, male and female participants in the MUSC cohort. The shaded circles show the high variant load gene counts identified in all participants.

The only regression analysis which could be performed using male participants alone from the MUSC cohort was the comparison of variant counts in men classified with Sensory hearing loss versus those classified with Metabolic hearing loss (Fig 2). Some of the genes with a high variant load in Sensory hearing loss are found in both the male and female lists, but none of the genes with a high variant load in Metabolic hearing loss are shared between the sexes (Fig 6). It is possible that there is a higher sex-specific genetic contribution to Metabolic hearing loss, but more data from larger cohorts are needed to explore this further. We also found multiple variants which appear to contribute to differences in thresholds in a sex-specific manner (Table 5), although the lack of audiograms and exome sequencing from male participants in the TwinsUK cohort means that we have not been able to follow up on those variants linked to threshold differences visible only in men from the MUSC cohort. Similarly well-characterised cohorts are necessary for further investigating the differing genetic contribution to hearing loss between the sexes.

### Candidate genes and variants associated with better hearing in older adults

Intriguingly, a subset of candidates from both outlier and threshold analyses are associated with better hearing, suggesting that some variants may lead to protection against age-related hearing loss and/or protection against damage from noise exposure. This is not the first report of protective variants. Examples of other protective variants include the N352S variant in *B4GALT1* which is protective against cardiovascular disease [51] and protein-truncating variants in *GPR75* which reduce the risk of obesity [52], as well as the A88V variant in *Gjb6*, which protects against hearing loss in mice [53]. Identification of genes and variants which protect against hearing loss could be a useful starting point for developing therapeutic treatments to do the same.

## Methods

### Ethics statement

All human subjects research was conducted in accordance with the Declaration of Helsinki. Written informed consent was obtained in this study, which was approved by the Medical University of South Carolina (MUSC) Institutional Review Board (for the MUSC cohort) and Guys & St Thomas’ Trust (GSTT) Ethics Committee (for the TwinsUK cohort).

All mouse experiments were carried out in accordance with UK Home Office regulations and the UK Animals (Scientific Procedures) Act of 1986 (ASPA) under UK Home Office licences, and the study was approved by the Wellcome Sanger Institute Ethical Review Committee.

### Participants and audiometric measurements

The primary cohort consisted of 532 volunteers enrolled in an ongoing longitudinal study of age-related hearing loss at MUSC, dating from 1987, described in detail in Dubno et al, 2013 [54]. Notably, no participants exhibited any sign of conductive hearing loss or active otologic disease. The 532 individuals were aged 55 years or older. Pure tone thresholds (at 0.25, 0.5, 1.0, 2.0, 3.0, 4.0, 6.0 and 8.0 kHz) were obtained for each ear of each person, along with questionnaire responses concerning noise exposure history.

For the follow-up cohort, we selected 159 participants from the TwinsUK study based on age (55 years and older), self-reported ethnicity (“White”), and availability of both exome and pure-tone audiometry data. The pure-tone audiometry data collection has previously been described [55]; briefly, all participants underwent an otologic examination followed by an air-conduction pure-tone audiogram for each ear (0.125, 0.25, 0.5, 1.0, 2.0, 4.0, 6.0 and 8.0 kHz). Participants also answered a detailed questionnaire concerning medical history and environmental exposure to factors relevant to hearing.

### Classification of audiograms

**Fig 7:**
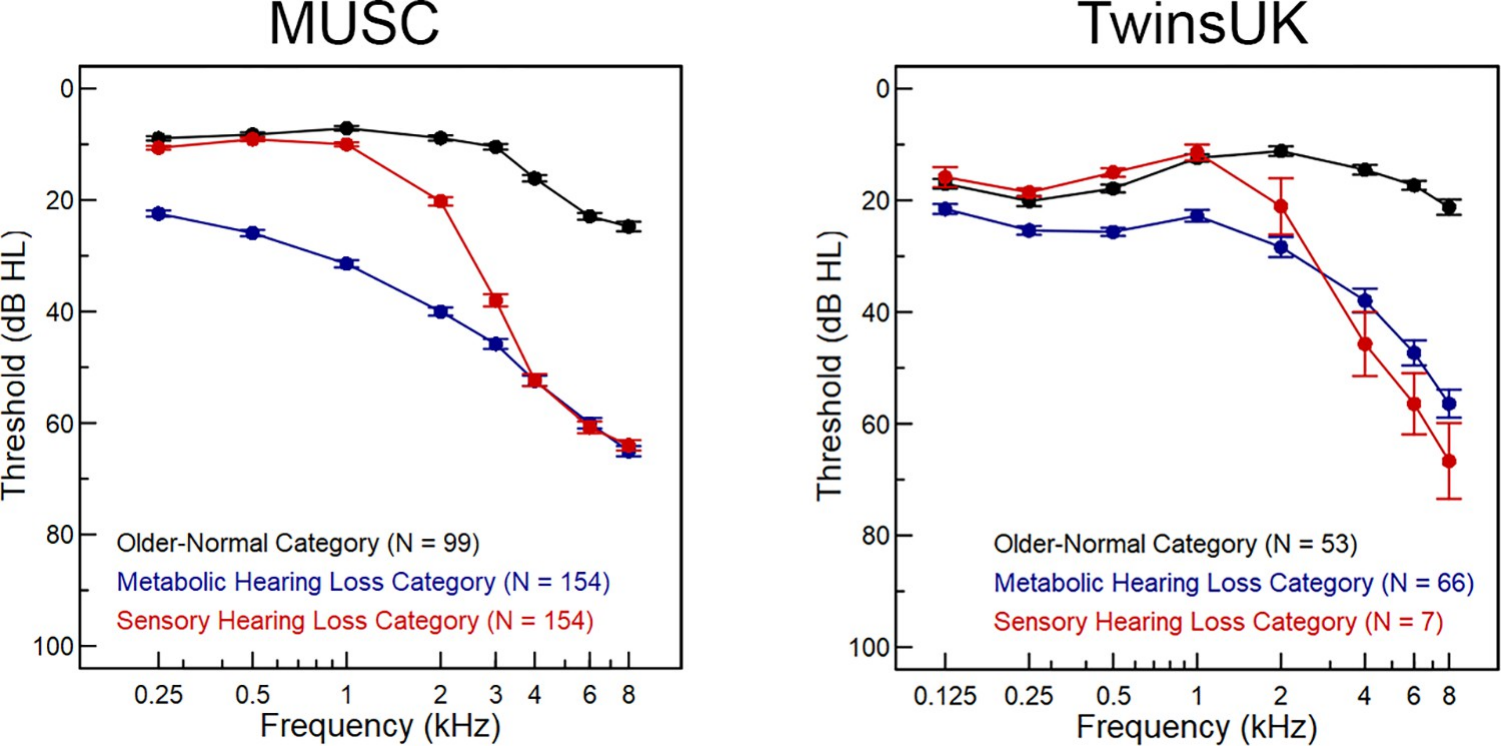
Mean audiograms for the three main classifications. Averages of thresholds for Older-Normal (black), Metabolic hearing loss (blue) and Sensory hearing loss (red) cases in the MUSC cohort (left) and the TwinsUK cohort (right). Error bars are standard error of the mean.

Phenotype cohorts were formed based on selection criteria to define individuals with representative metabolic or sensory hearing losses, as well as normal hearing, to enable comparisons between specific phenotypes. Audiograms were classified into one of three main categories (Older-Normal, Metabolic, and Sensory, Fig 7) based on the estimated metabolic and sensory components of the observed hearing loss [14]. The typical audiogram in metabolic cases shows mildly elevated thresholds at low frequencies sloping gently downwards towards higher frequencies, while the shape of a typical sensory pattern has normal thresholds at low frequencies and steeply downwards-sloping thresholds at high frequencies [54,56,57]. These typical profiles (obtained from 402 older adult audiograms [14]) can be used to approximate the metabolic and sensory components of the hearing loss observed in an individual ear. It is then possible to calculate the contribution of each profile to this approximation, and the quality of the approximation itself is represented by the line-fit error.

To classify these cohorts, first, metabolic and sensory estimates and line-fit error were calculated for each of the right/left pairs of audiograms. Second, poorly fit audiograms were excluded from classification using the criterion of line-fit error ≥15 dB, which identifies audiograms with configurations inconsistent with age-related hearing loss (e.g., corner audiograms). These rejected audiograms are referred to as “Unselected” below. Third, a set of simple rules (below) using the metabolic and sensory estimates were applied to classify cases into the four remaining categories.

Using this approach, the Older-Normal category was defined by cases with summed metabolic + sensory estimates that were <20 dB HL, with <10 dB difference in the estimates between ears. The Metabolic category was selected from the remaining cases (i.e., not Older-Normal) with metabolic estimates ≥20 dB, ear asymmetries in the metabolic estimate ≤15 dB, sensory estimates <20 dB, and metabolic > sensory estimates. The Sensory category was selected from the remaining cases (i.e., not Older-Normal and not Metabolic) with sensory estimates ≥15 dB, ear asymmetries in the sensory estimate ≤20 dB, metabolic estimates <25 dB, and sensory > metabolic estimates. Finally, the remaining cases (i.e., not Older-Normal, not Metabolic, not Sensory) were less clearly representative of metabolic or sensory hearing loss, and were labelled Unclassified. After classifying the audiograms based on these rules, all audiograms in each category were reviewed by eye (KIV, JRD); a few anomalous cases were removed and a few cases were added based on consistency with a category. There were a total of 1–10 manual additions or removals for each category (S7 and S8 Figs).

### Exome sequencing and alignment

Libraries for exome sequencing of the MUSC cohort were prepared using the Agilent SureSelect X2 Target Enrichment System (version 5) and the Agilent SureSelect Human All Exon V5 kit, which included 5’ and 3’ UTRs. DNA was sheared using the Covaris S220 focused ultrasonicator. Libraries were sequenced on the Illumina HiSeq 2500.

The exome sequencing of the Twins UK cohort has been previously described [58]. Briefly, DNA extracted from whole blood was hybridised to NimbleGen human exome arrays and sequenced using Illumina sequencing machines (NimbleGen 2.1M and the Illumina GAIIx for the first batch of sequencing, and NimbleGen EZ v2 and the HiSeq 2000 for the second).

For both cohorts, fastq files were aligned to GRCh38 using Hisat2.0 [59], following quality control steps (S7 Table). Bam files were realigned to sex-corrected genomes using XYalign [60].

### Variant calling, filtering, annotation and confirmation

After read alignment, genomic variants were called using three callers; GATK HaplotypeCaller [61,62], BCFtools [63] and Freebayes [64] (S7 Table). Combining calls from multiple callers has been shown to offer more accurate variant calling [65]. HaplotypeCaller quality scores were recalibrated using the GATK Variant Quality Score Recalibrator (VQSR) tool [66], which annotates variants into tranches which represent subsequent levels of sensitivity versus specificity. Variants in the highest tranche are very high accuracy, very likely to be true but also incomplete (high specificity, lower sensitivity). The second tranche is less specific but more sensitive, and so on (https://gatk.broadinstitute.org/hc/en-us/sections/360007226651-Best-Practices-Workflows). BCFtools calls were filtered using vcfutils [63], and Freebayes calls were filtered using vcftools [67] (S7 Table).

After quality filtering each set of calls in the MUSC cohort, a selection of variants representing a range of quality scores was tested by Sanger sequencing to ascertain the best combination of filters. The resequenced variants were assessed based on whether the variant was correctly identified and also whether the sample genotypes were correctly called. We obtained 184 sequences from 66 variants, and the most accurate variant calls were those which had passed the GATK VQSR filters and had also passed at least one of the BCFtools or Freebayes filters. Those variants from the second GATK VQSR tranche which had passed the BCFtools filter were also found to be accurately called. The variant calls from all three callers were combined according to these requirements, and this combination filter was implemented for both the MUSC and the TwinsUK cohorts (S1 Table). Where there was a genotype disagreement (eg GATK and Freebayes called 0/1 and BCFtools called 1/1), the majority call was accepted. Calls like this, and calls with no disagreement, accounted for 99.8% (MUSC) and 85.5% (TwinsUK) of total calls. Where there were three different calls, one for homozygote alternate, one for homozygote reference and one for heterozygote, a heterozygous genotype was assigned (0.00025% of calls (MUSC); 0.00046% of calls (TwinsUK)). Other call combinations were considered missing (0.19% (MUSC); 14.5% (TwinsUK)). The reason for the TwinsUK sequencing having a higher call missing rate is due to the exome sequencing having been processed in two batches with different exome arrays [58], so some variants have only been called in half the participants.

941,165 (MUSC) and 281,261 (TwinsUK) variants passed these quality filters, and were then tested for excess heterozygosity using the R HardyWeinberg package [68,69] to identify and remove misaligned variants [70]. Also excluded were variants which had a high allele frequency in their cohort (defined as variants with cohort allele frequency above minor allele frequency (MAF)+0.4), which are likely to be aligner miscalls in low-complexity regions [71].

Mitochondrial variants were called using GATK Mutect2 [72] and filtered using GATK FilterMutectCalls. Although none of the kits used (Agilent SureSelect All Exon v5, NimbleGen 2.1M and NimbleGen EZv2) include the mitochondrial chromosome, off-target reads have been found to map correctly [73]. Griffin et al [74] tested this using three different exome kits (including the Agilent SureSelect Human All Exon 50Mb kit and the NimbleGen SeqCap EZ Exome Library v2.0) and conventional mitochondrial DNA sequence, and found that if the coverage was high enough (>30x), heteroplasmy over 40% could be reliably detected. The mitochondrial calls were therefore further filtered by read depth and variant allele fraction (S1 Table). For the two analyses carried out (described below), homoplasmic variants were treated as homozygote calls and heteroplasmic variants as heterozygote calls.

Genomic and mitochondrial variants were annotated using the Ensembl Variant Effect Predictor (VEP) v100 [75]. Annotation sources included 5’UTR variant prediction (Sutr, [76]), splice site variant prediction (SpliceAI, [77]), pathogenicity prediction (CADD, [78]) and minor allele frequency (gnomAD, TOPMED, ESP6500 and 1000Genomes [16,19,79,80]). The final filter, for high impact variants with MAF < 0.1, was based on our previous work (S2 Table).[1]

Chosen variants from the MUSC cohort were resequenced using Sanger sequencing (carried out by Eurofins Genomics LLC, Kentucky, USA). Primers for Sanger sequencing were designed using primer3 [81], and sequence traces were checked using Gap4 [82].

### Regression analysis of number of variants per gene

Four comparisons were carried out: Older-Normal hearing to all hearing loss (including Unselected and Unclassified participants); Older-Normal hearing to Metabolic hearing loss; Older-Normal hearing to Sensory hearing loss; and Metabolic hearing loss to Sensory hearing loss. 12176 genes (including mitochondrial genes) had at least one variant called in one sample, and were assessed in each analysis. For each comparison, a linear regression was carried out on the total number of variants per gene per group. In the first three comparisons, the number of variants in the Older-Normal hearing group was used to predict the expected number of variants in the hearing loss group, and in the fourth comparison, the number of variants in the Metabolic hearing loss group was used to predict the expected number of variants in the Sensory hearing loss group. The residuals (the difference between the observed and predicted variant load for each gene) were used to determine the outlier genes. Briefly, the first (Q1) and third (Q3) quartile and the interquartile distance D (Q3-Q1) were calculated for each regression’s residuals, and outlier genes were defined as those with residuals > Q3 + 6D and those with residuals < Q1 – 6D [83]. Hypergeometric tests for enrichment were carried out using R.

#### Compilation of the list of known deafness genes

The list of deafness genes consists of those genes known to underlie hearing impairment in humans or in mice, and was manually compiled and curated from the literature. It includes all the genes listed in the Hereditary Hearing Loss Homepage (hereditaryhearingloss.org/) and genes which, when mutated, result in altered hearing thresholds in mutant mice, as reported by the International Mouse Phenotyping Consortium (www.mousephenotype.org) (average thresholds were individually checked for shifts > 10dB and low variance between individuals). This list is an update of that reported in our previous study [1]; it consists of 519 genes linked to hearing impairment in mice, 102 genes linked to hearing impairment in humans, and 128 genes linked to hearing impairment in both mice and humans (Fig 3, S6 Table).

### Expression analysis of outlier genes

Gene expression in the mouse inner ear was assessed using single cell RNAseq data obtained from the gEAR portal (https://umgear.org [18]). Datasets were chosen to include multiple ages (embryonic day (E)16, postnatal day (P)1, P7 [84], P15 [85], P20 [86] and P30 [87]), and expression was normalised within each dataset and cell type to *Hprt* expression. Where a dataset had more than one set of measurements for a cell type (eg the E16 dataset has “OHC_1” and “OHC_2”, both representing outer hair cells), expression levels were averaged. The expression of each gene was plotted in 12 different cell types, as defined by the original experiments. Eleven marker genes were plotted for comparison (hair cells: *Myo7a*; inner hair cells: *Fgf8*; outer hair cells: *Slc26a5*; non-sensory cells: *Sox2*; inner pillar cells: *S100b*; Deiters’ cells: *Hes5*; marginal cells: *Kcne1*; intermediate cells: *Met*; basal cells: *Cldn11*; spindle and root cells: *Slc26a4*; fibrocytes: *Gm525*). These are known marker genes for their cell types, with the exception of *Gm525*, which was chosen based on its fibrocyte-specific expression at P30 [87].

### Immunohistochemistry

Wildtype mice from the C57BL/6N strain were used for the Madd expression study. Animals were collected, fixed, embedded in paraffin wax and sectioned as described in [88], with the only difference being the postnatal ages chosen (P0 and P4). Sections were dewaxed and rehydrated, then subjected to heated antigen retrieval in Citric acid after endogenous peroxidase blocking. Sections were then incubated with anti-Madd (1:200, AbCam, cat. no. ab134117) for 16 hours at room temperature. The secondary antibody used was Goat anti-rabbit IgG antibody (H+L) (1:5000, Vector Laboratories, BA-1000). Sections were stained using the Vectastain Elite ABC kit (Vector Laboratories, cat. no. PK-6100), counterstained with haemalum, dehydrated, and mounted in DPX. Images were taken using a Zeiss Axioskop 2 microscope with a Zeiss Axiocam camera, and processed using Adobe Photoshop CS6; minimal adjustments were made, including rotating and resizing.

### Threshold difference detection

To assess each individual variant, audiograms were plotted with participants separated into groups by genotype and sex. Variants with fewer than 5 people/group in all alternate allele groups were excluded. For each group, the average thresholds at each stimulus frequency were compared to the reference group, testing for a difference of 20dB or more. This is a greater average threshold difference than that seen between those reporting a positive noise history and those reporting a negative noise history (Fig 1C). A maximum limit was imposed on standard deviation in the alternate allele group which differed by stimulus frequency (15 dB for 0.125–0.5 kHz, 20 dB for 1–2 kHz, 25 dB for 3–4 kHz, 30 dB for over 4 kHz) to prioritise variants associated with consistent threshold patterns. All variants where at least two stimulus frequencies in each ear passed this filter were put through to permutation testing.

We carried out 20,000 permutations for each variant to assess the likelihood that those stimulus frequencies which passed the filter were observed by chance. For each permutation, individuals from the cohort were assigned randomly to groups of the same number and sex as the original alternate allele and reference groups, and the relevant stimulus frequencies tested using the same settings as before. If more than 1000 random shufflings produced a similar result (i.e. p > 0.05), the variant was rejected. This was carried out automatically, and the scripts can be found at github.com/moraglewis/ThreADD.

The final p-values, derived from the permutation testing, represent the likelihood that the differences between the alternate and reference allele groups arose by chance. For the MUSC cohort, which had 41 variants passing the permutation test, we corrected for multiple testing using the Benjamini-Hochberg correction.

## Supporting information

S1 Table Tools and settings used for filtering variants by quality and impact. (XLSX)

S2 Table Outlier genes from the MUSC cohort analyses. (XLSX)

S3 Table Variant counts per gene from the MUSC cohort. (XLSX)

S4 Table Outlier genes from the TwinsUK cohort analyses. (XLSX)

S5 Table Variant counts per gene from the TwinsUK cohort. (XLSX)

S6 Table Genes known to underlie hearing loss in humans and/or in mice. (XLSX)

S7 Table Details of exome sequence data processing. (XLSX)

S1 Fig Both cohorts are predominantly of Non-Finnish European ancestry. Population principal component analysis for the MUSC (orange, A) and TwinsUK (red, B) cohorts, along with data from participants from different genetic ancestries in the 1000 Genomes project [16]. The global population distribution is shown on the left and the distribution of European subpopulations on the right.
(PDF)

S2 Fig Schematic of the pipeline used for variant calling and filtering. See S1 Table for details of the filter settings.
(PDF)

S3 Fig Expression levels at different developmental stages of the mouse orthologues of ten genes of interest from the outlier analysis (*Syne2*, *Atp2c2*, *Dhrs4* (*DHRS4L2*), *Rasal1*, *Patj* (*INADL)*, *Tacc2*, *Abcb8*, *Itsn2*, *Pkhd1l1*, and *Fkbp2*). Single cell RNAseq data from the gEAR (http://umgear.org) was plotted for each of the ten genes. Expression was normalised to *Hprt* (represented by a horizontal line at y = 1 on each plot). Marker genes included for comparison are *Myo7a* (hair cells), *Fgf8* (inner hair cells), *Slc26a5* (outer hair cells), *Sox2* (non-sensory cells), *S100b* (inner pillar cells), *Hes5* (Deiters’ cells), comparison (*Kcne1* (marginal cells), *Met*(intermediate cells), *Cldn11* (basal cells), *Slc26a4* (spindle and root cells) and *Gm525* (fibrocytes). Two sets of plots are presented; the first set show expression in organ of Corti cell types and the second show expression in lateral wall cell types.
(PDF)

S4 Fig Expression levels at different developmental stages of genes linked to specific subtypes of hearing loss. Single cell RNAseq data from the gEAR (http://umgear.org) was plotted for each of the 29 genes associated with Metabolic (M) or Sensory (S) hearing loss. Expression was normalised to *Hprt* (represented by a horizontal line at y = 1 on each plot). Marker genes included for comparison are *Myo7a* (hair cells), *Fgf8* (inner hair cells), *Slc26a5* (outer hair cells), *Sox2* (non-sensory cells), *S100b* (inner pillar cells), *Hes5* (Deiters’ cells), comparison (*Kcne1* (marginal cells), *Met*(intermediate cells), *Cldn11* (basal cells), *Slc26a4* (spindle and root cells) and *Gm525* (fibrocytes).
(PDF)

S5 Fig Madd is expressed in the hair cells of the mouse inner ear. Images of the cochlear duct (basal turn) at ages from E14.5 (where no staining is visible) to P4. At least three mice were examined at each age. Brown shows where Madd is present (visible from E16.5 and older); hair cells are marked with arrowheads. Scale bar = 20μm.
(PDF)

S6 Fig All audiograms from the MUSC and TwinsUK cohorts plotted in groups by sex and genotype. For the 41 variants identified in the MUSC cohort, audiograms of TwinsUK carriers are shown on the right where available. The variant in *HADH* was identified in both cohorts. For the remaining 3 variants identified in the TwinsUK cohort, the audiograms of MUSC carriers are shown on the left. Two audiograms are shown for each variant in each cohort; the thresholds from the left ear are shown on the left, and those from the right ear on the right. Numbers and average ages of each group are listed on the graph. The symbols at the top of each graph mark which groups passed the criteria for each stimulus frequency compared to the relevant reference group (+ for male, = for female, and * for all participants). Error bars are standard deviation.
(PDF)

S7 Fig MUSC cohort classification. A) Schematic showing the classification process and numbers at each stage. B) Plot of the sensory estimate against the metabolic estimate for each well-fit case, with the blue/red shading indicating the magnitude of each estimate. The small dots are the Unclassified cases. C) shows the mean audiograms for the cases assigned to each category (error bars are standard error of the mean).
(PDF)

S8 Fig TwinsUK cohort classification. A) Schematic showing the classification process and numbers at each stage. B) Plot of the sensory estimate against the metabolic estimate for each well-fit case, with the blue/red shading indicating the magnitude of each estimate. The small dots are the Unclassified cases. C) shows the mean audiograms for the cases assigned to each category (error bars are standard error of the mean).
(PDF)

S1 Data Data for audiograms in Fig 1. (XLSX)

S2 Data Data for audiograms in Figs 7, S7 and S8. (XLSX)

S3 Data Data for Figs 5 and S6. (XLSX)
