# Supplementary material for: Accurate phenotypic classification and exome sequencing allow identification of novel genes and variants associated with adult-onset hearing loss
Source: PLoS Genet. 2023 Nov 27;19(11):e1011058. doi: 10.1371/journal.pgen.1011058 (PMC10718637; doi:10.1371/journal.pgen.1011058)
Supplement: S1 Fig — Population principal component analysis for the MUSC (orange, A) and TwinsUK (red, B) cohorts, along with data from participants from different genetic ancestries in the 1000 Genomes project [16]. The global population distribution is shown on the left and the distribution of European subpopulations on the right. (PDF) [file pgen.1011058.s008.pdf]

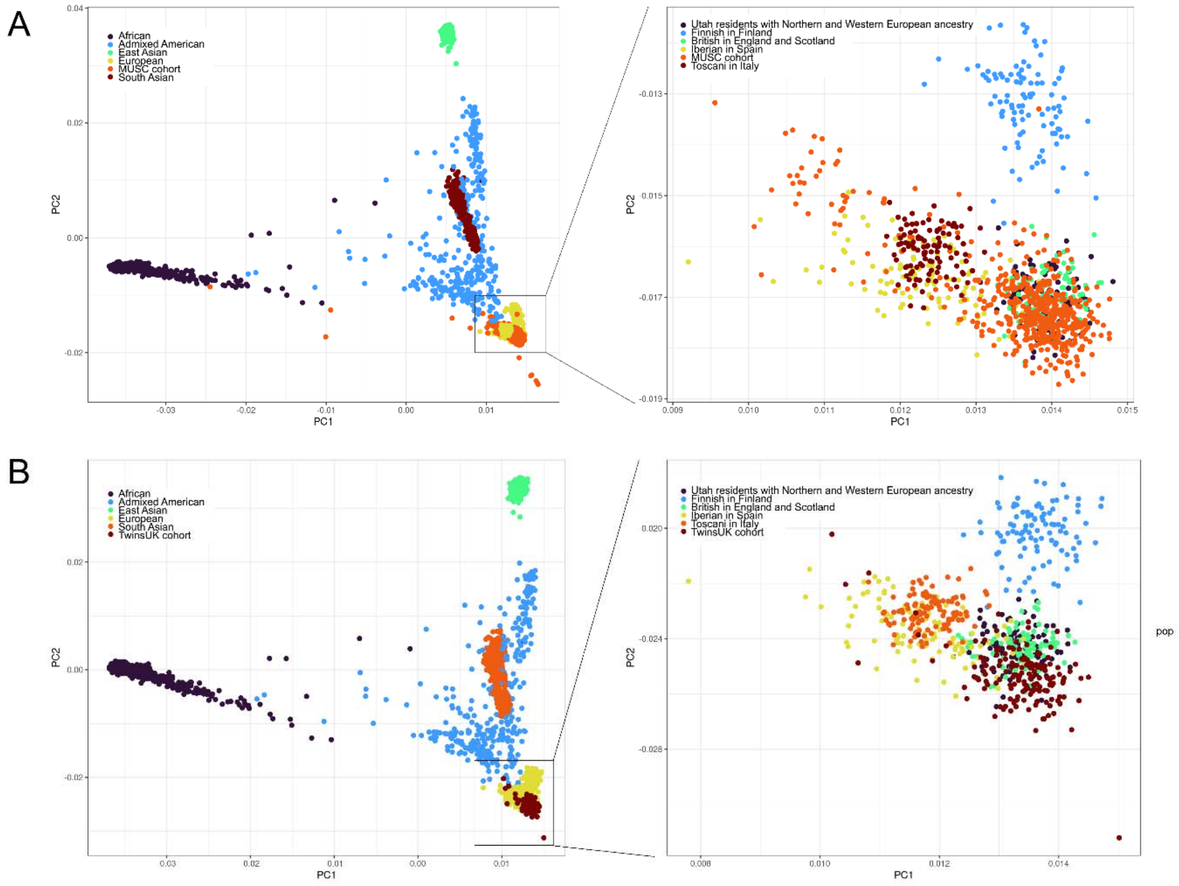

S1 Fig. Both cohorts are predominantly of Non-Finnish European ancestry. Population principal component analysis for the MUSC (orange, A) and TwinsUK (red, B) cohorts, along with data from participants from different genetic ancestries in the 1000 Genomes project [16]. The global population distribution is shown on the left and the distribution of European subpopulations on the right.
