# Supplementary material for: Accurate phenotypic classification and exome sequencing allow identification of novel genes and variants associated with adult-onset hearing loss
Source: PLoS Genet. 2023 Nov 27;19(11):e1011058. doi: 10.1371/journal.pgen.1011058 (PMC10718637; doi:10.1371/journal.pgen.1011058)
Supplement: S2 Fig — See S1 Table for details of the filter settings. (PDF) [file pgen.1011058.s009.pdf]

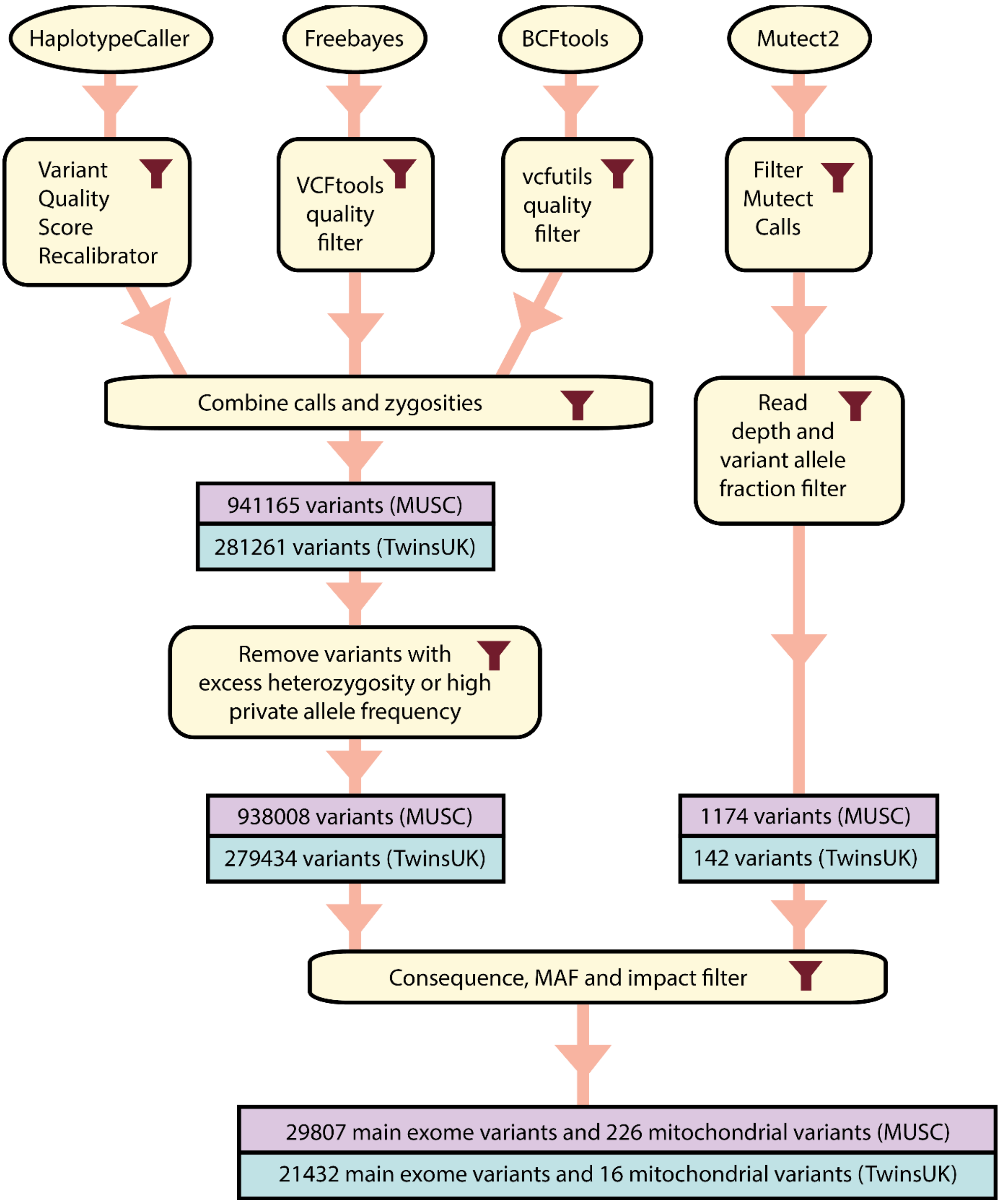

S2 Fig. Schematic of the pipeline used for variant calling and filtering. See S1 Table for details of the filter settings.
