# Supplementary material for: Accurate phenotypic classification and exome sequencing allow identification of novel genes and variants associated with adult-onset hearing loss
Source: PLoS Genet. 2023 Nov 27;19(11):e1011058. doi: 10.1371/journal.pgen.1011058 (PMC10718637; doi:10.1371/journal.pgen.1011058)
Supplement: S3 Fig — Single cell RNAseq data from the gEAR (http://umgear.org) was plotted for each of the ten genes. Expression was normalised to Hprt (represented by a horizontal line at y = 1 on each plot). Marker genes included for comparison are Myo7a (hair cells), Fgf8 (inner hair cells), Slc26a5 (outer hair cells), Sox2 (non-sensory cells), S100b (inner pillar cells), Hes5 (Deiters’ cells), comparison (Kcne1 (marginal cells), Met(intermediate cells), Cldn11 (basal cells), Slc26a4 (spindle and root cells) and Gm525 (fibrocytes). Two sets of plots are presented; the first set show expression in organ of Corti cell types and the second show expression in lateral wall cell types. (PDF) [file pgen.1011058.s010.pdf]

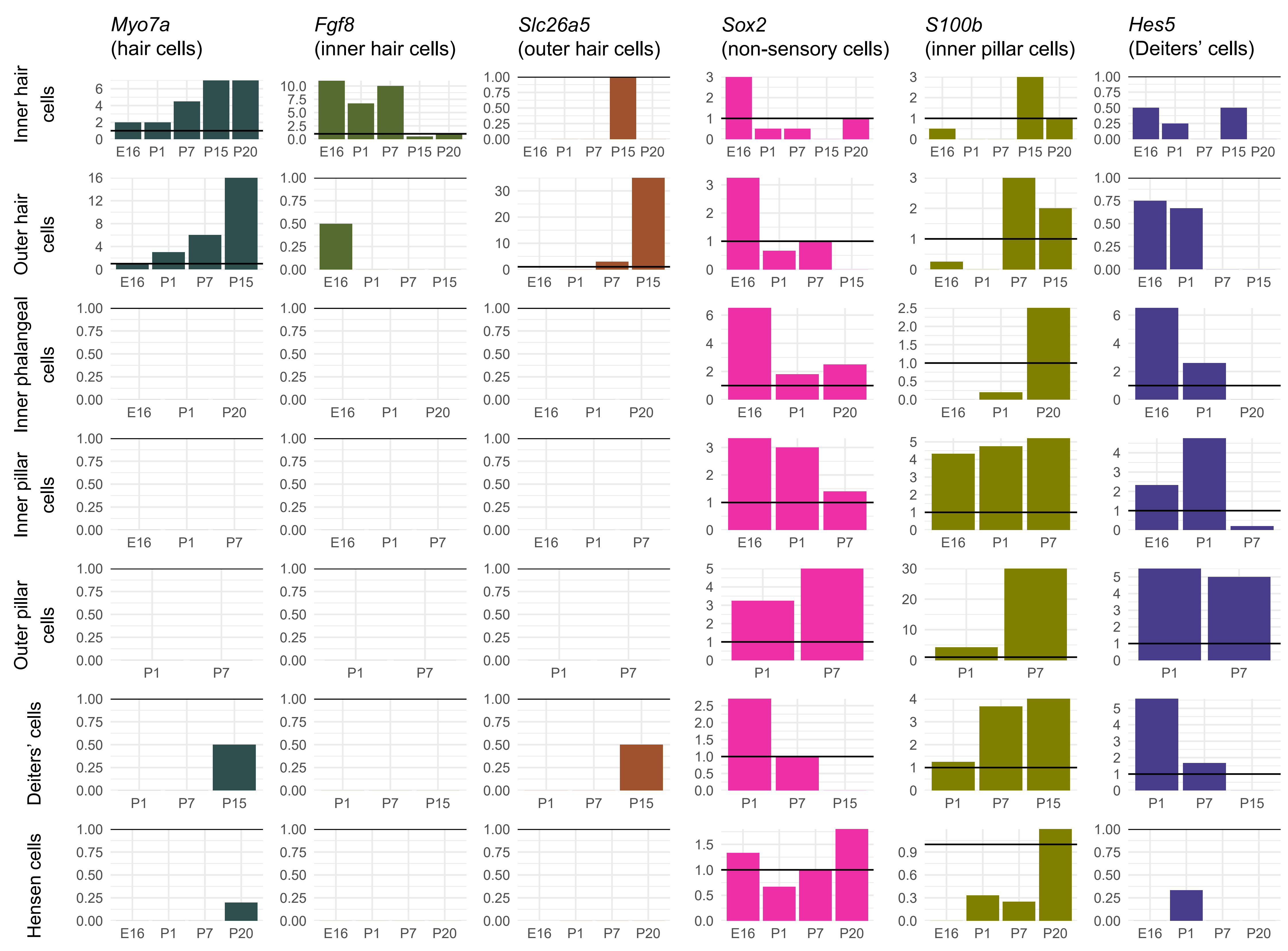

Myo7a (hair cells)
Fgf8 (inner hair cells)
Slc26a5 (outer hair cells)
Sox2 (non-sensory cells)
S100b (inner pillar cells)
Hes5 (Deiters' cells)
Inner hair cells
Outer hair cells
Inner phalangeal cells
Inner pillar cells
Outer pillar cells
Deiters' cells
Hensen cells
E16
P1
P7
P15
P20

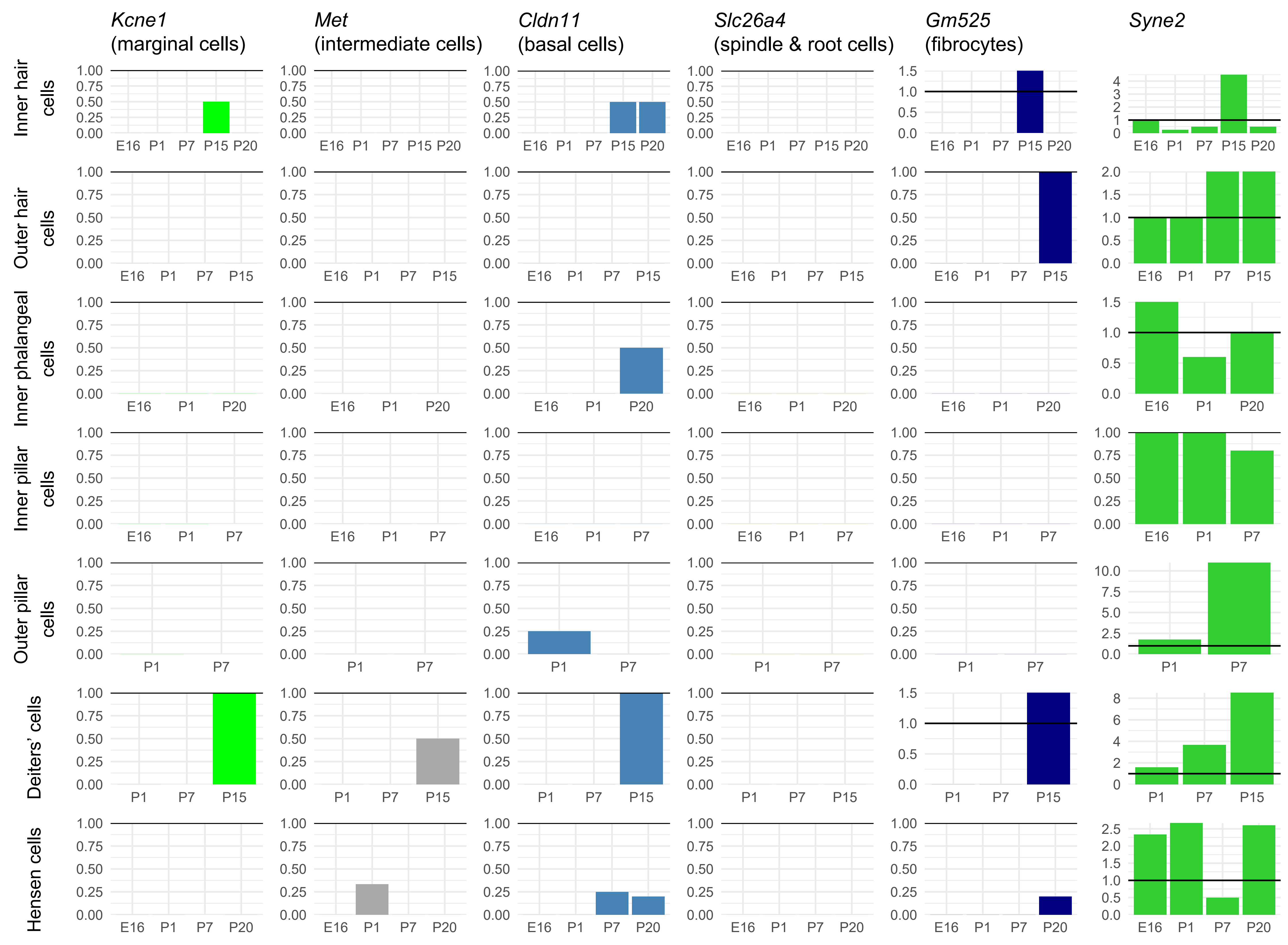
Kcne1
(marginal cells)
Met
(intermediate cells)
Cldn11
(basal cells)
Slc26a4
(spindle & root cells)
Gm525
(fibrocytes)
Syne2
Inner hair cells
Outer hair cells
Inner phalangeal cells
Inner pillar cells
Outer pillar cells
Deiters' cells
Hensen cells
E16
P1
P7
P15
P20

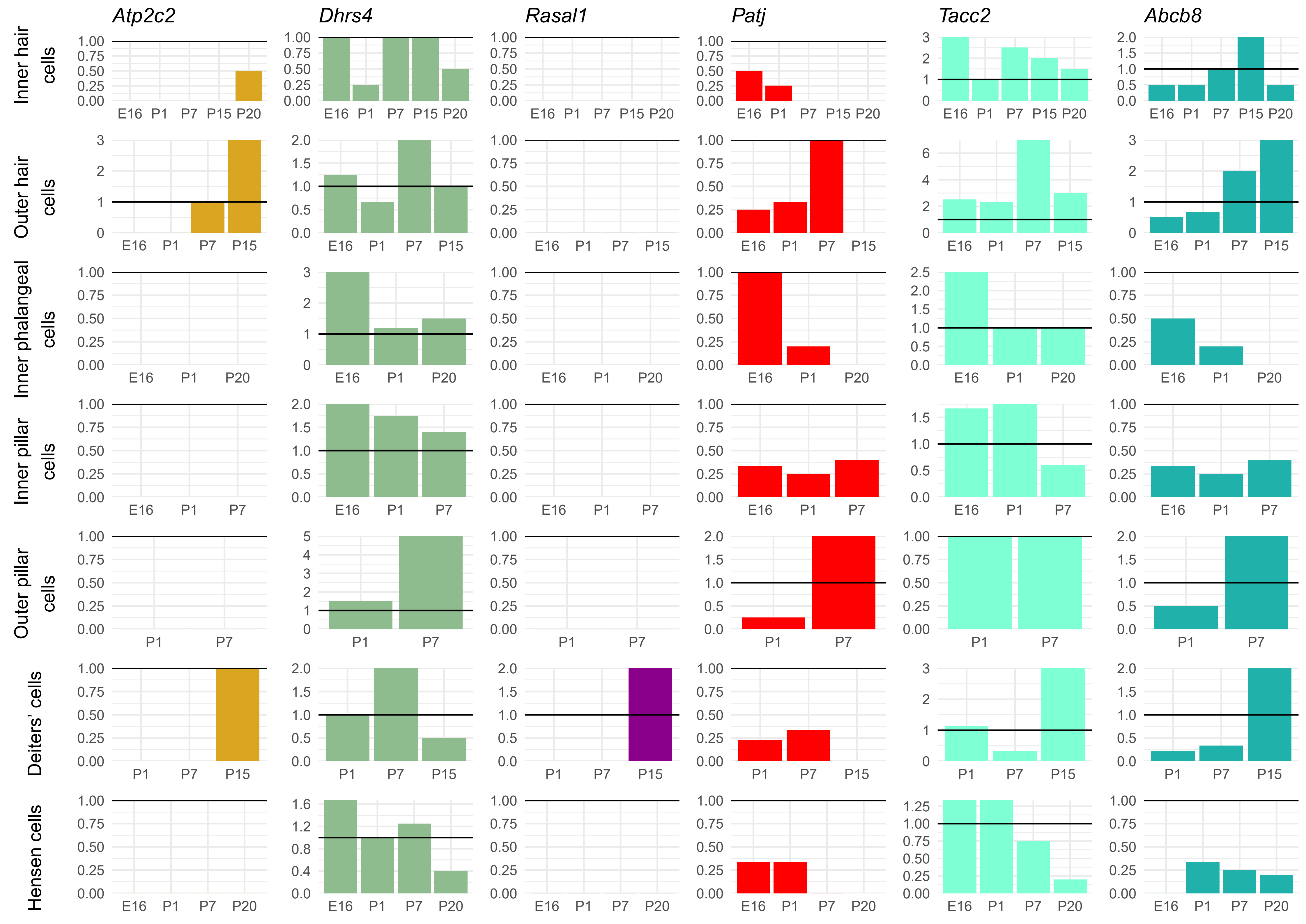

Atp2c2
Dhrs4
Rasal1
Patj
Tacc2
Abcb8
Inner hair cells
Outer hair cells
Inner phalangeal cells
Inner pillar cells
Outer pillar cells
Deiters' cells
Hensen cells
E16
P1
P7
P15
P20

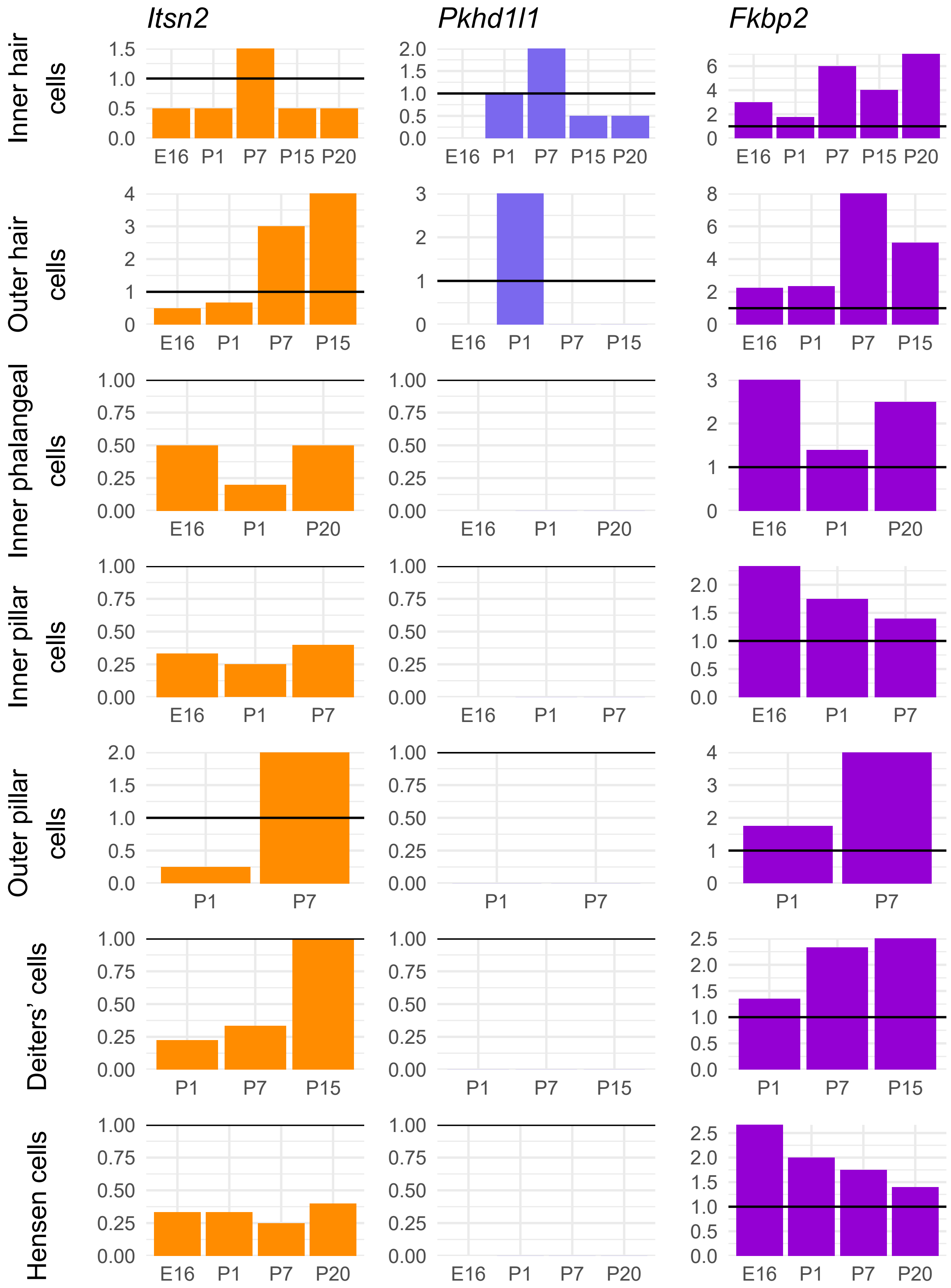

Itsn2
Pkhd1l1
Fkbp2
Inner hair cells
Outer hair cells
Inner phalangeal cells
Inner pillar cells
Outer pillar cells
Deiters' cells
Hensen cells
E16
P1
P7
P15
P20

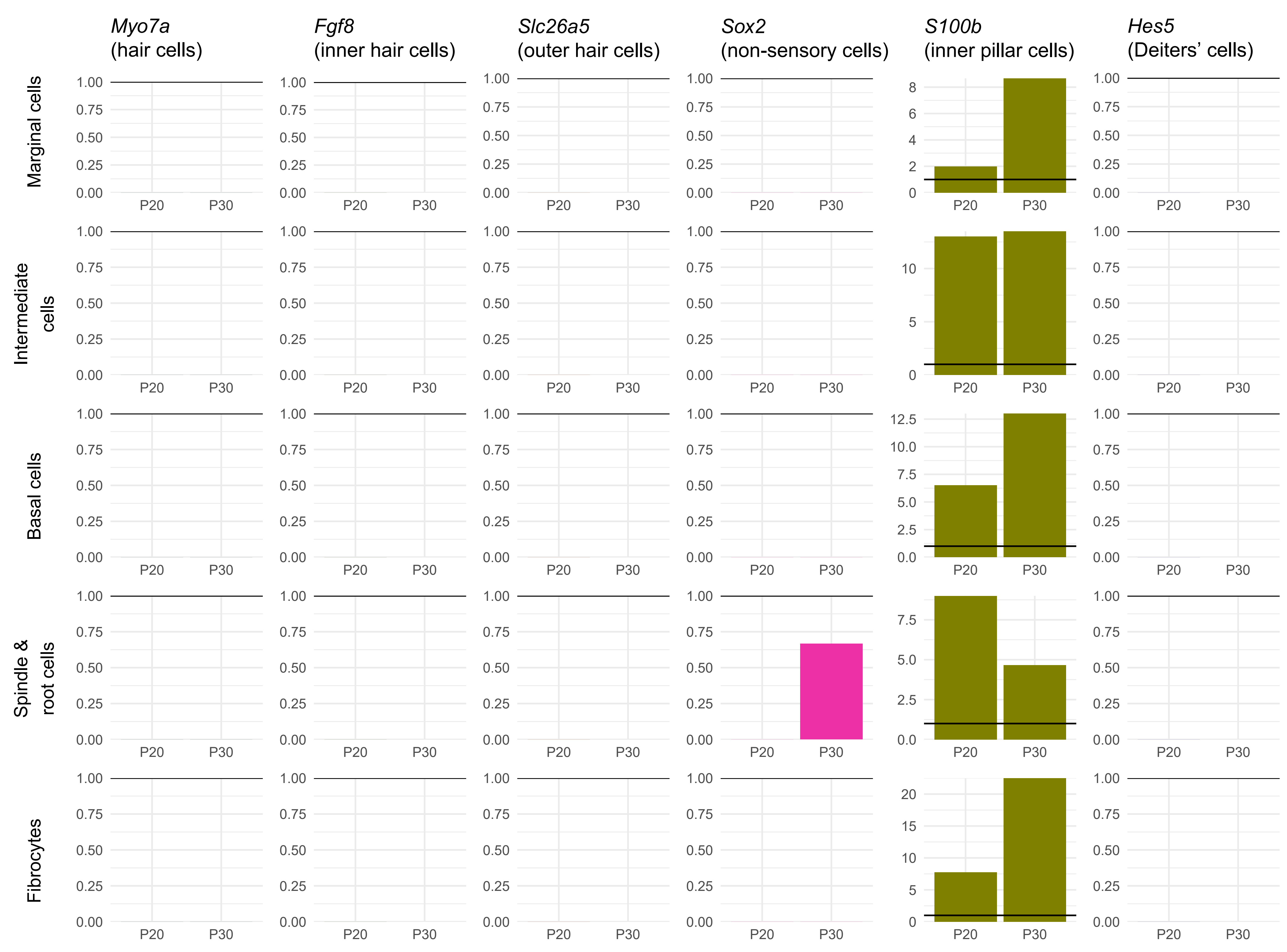

Myo7a (hair cells)
Fgf8 (inner hair cells)
Slc26a5 (outer hair cells)
Sox2 (non-sensory cells)
S100b (inner pillar cells)
Hes5 (Deiters' cells)
Marginal cells
Intermediate cells
Basal cells
Spindle & root cells
Fibrocytes
P20
P30

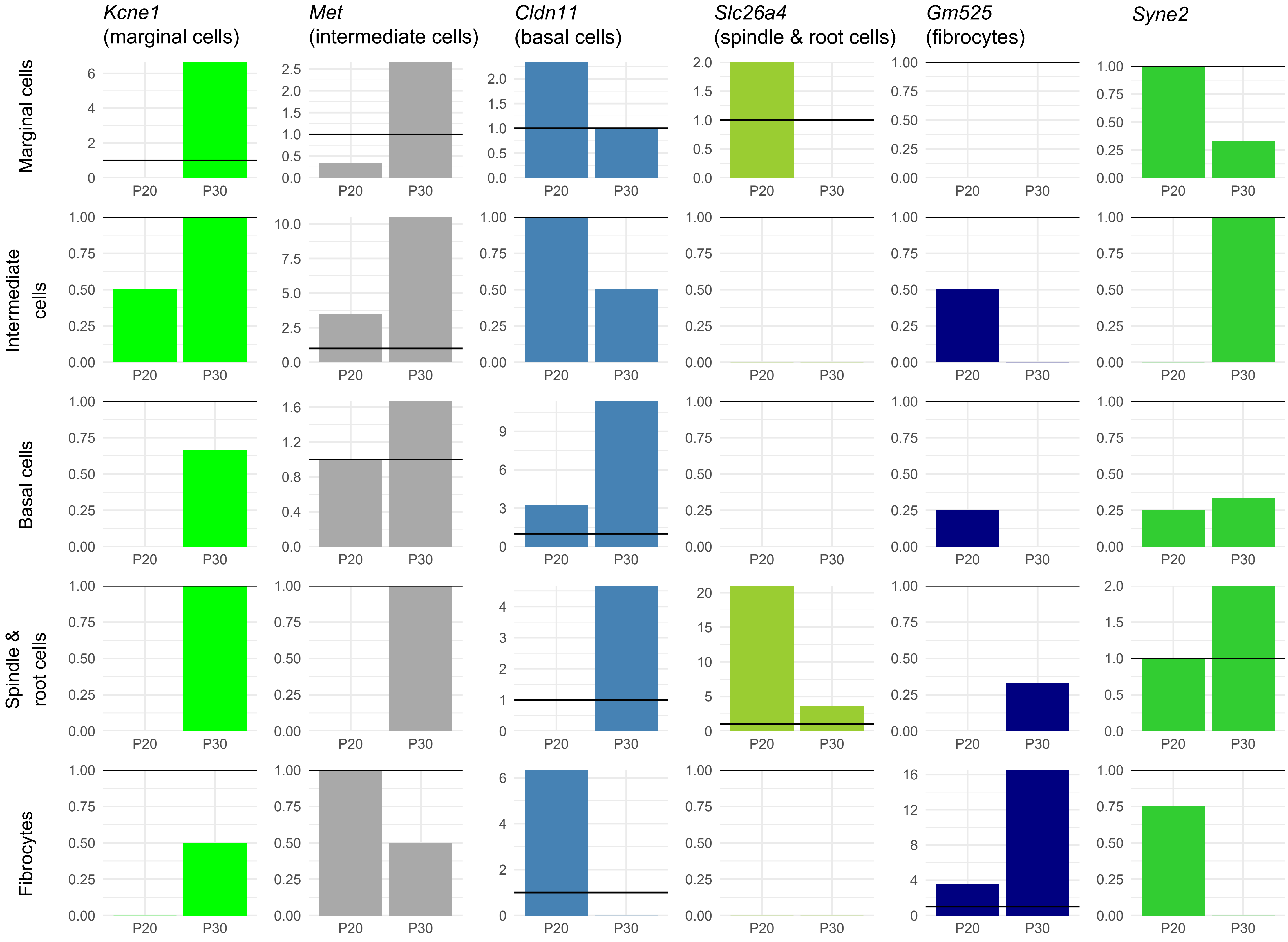

Kcne1
(marginal cells)
Met
(intermediate cells)
Cldn11
(basal cells)
Slc26a4
(spindle & root cells)
Gm525
(fibrocytes)
Syne2
Marginal cells
Intermediate cells
Basal cells
Spindle & root cells
Fibrocytes
P20
P30

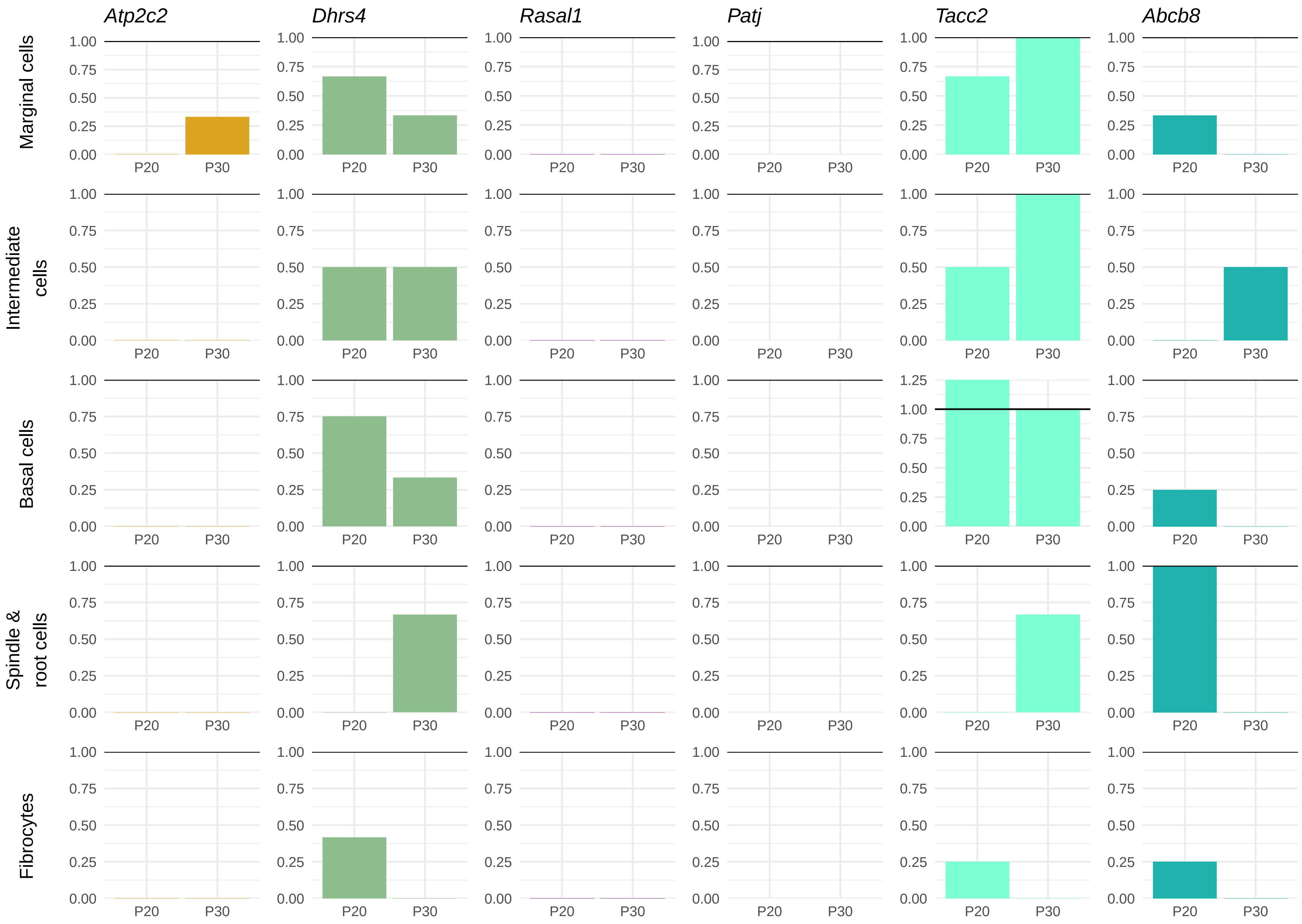
Atp2c2
Dhrs4
Rasal1
Patj
Tacc2
Abcb8
Marginal cells
Intermediate cells
Basal cells
Spindle & root cells
Fibrocytes
0.00
0.25
0.50
0.75
1.00
1.25
P20
P30

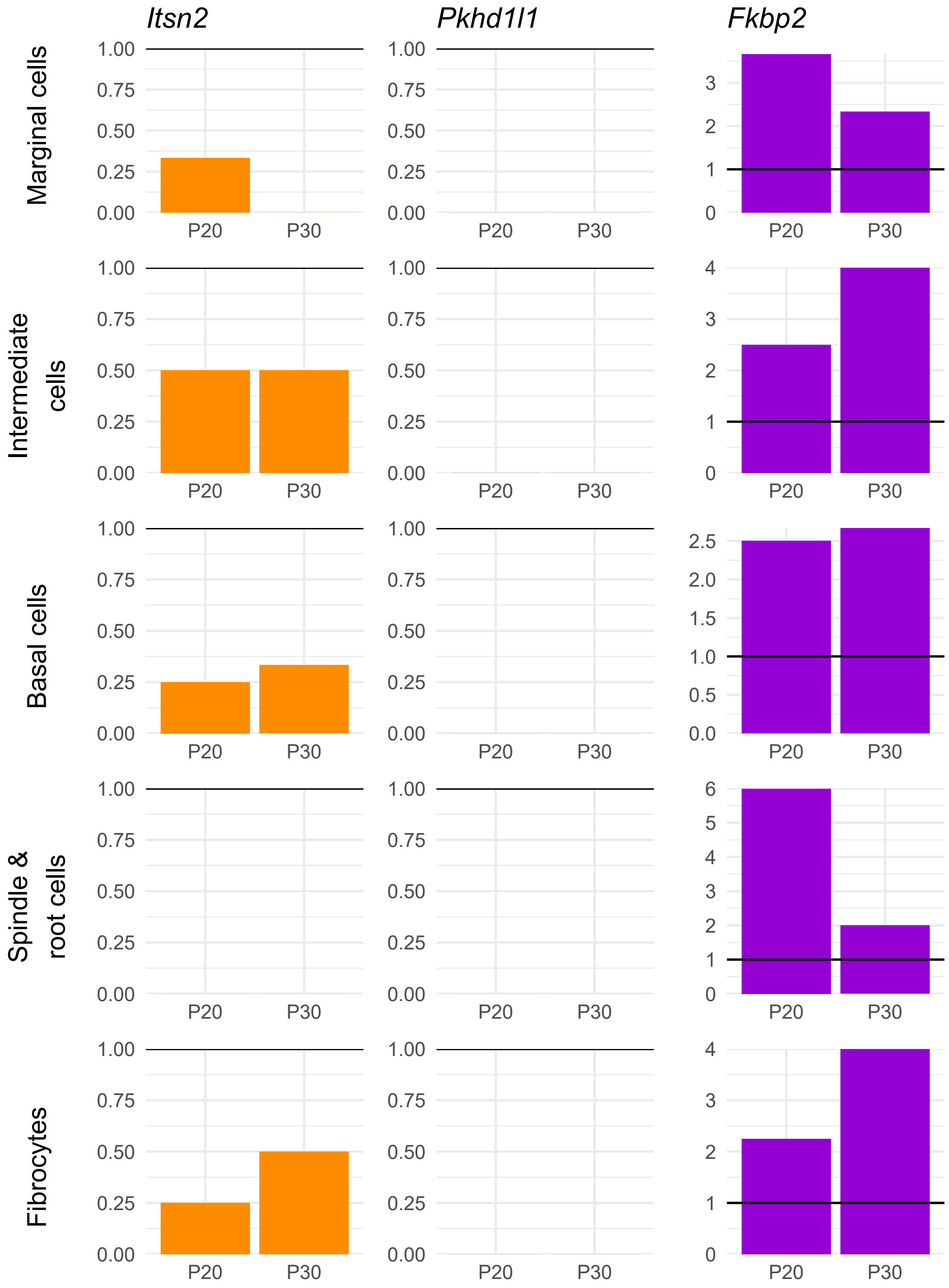

Itsn2
Pkhd1l1
Fkbp2
Marginal cells
Intermediate cells
Basal cells
Spindle & root cells
Fibrocytes
P20
P30
1.00
0.75
0.50
0.25
0.00

S3 Fig. Expression levels at different developmental stages of the mouse orthologues of ten genes of interest from the outlier analysis (*Syne2*, *Atp2c2*, *Dhrs4* (*DHRS4L2*), *Rasal1*, *Patj* (*INADL)*, *Tacc2*, *Abcb8*, *Itsn2*, *Pkhd1l1*, and *Fkbp2*). Single cell RNAseq data from the gEAR (http://umgear.org) was plotted for each of the ten genes. Expression was normalised to *Hprt* (represented by a horizontal line at y=1 on each plot). Marker genes included for comparison are *Myo7a* (hair cells), *Fgf8* (inner hair cells), *Slc26a5* (outer hair cells), *Sox2* (non-sensory cells), *S100b* (inner pillar cells), *Hes5* (Deiters' cells), comparison (*Kcne1* (marginal cells), *Met*(intermediate cells), *Cldn11* (basal cells), *Slc26a4* (spindle and root cells) and *Gm525* (fibrocytes). Two sets of plots are presented; the first set show expression in organ of Corti cell types and the second show expression in lateral wall cell types.
