# Supplementary material for: Accurate phenotypic classification and exome sequencing allow identification of novel genes and variants associated with adult-onset hearing loss
Source: PLoS Genet. 2023 Nov 27;19(11):e1011058. doi: 10.1371/journal.pgen.1011058 (PMC10718637; doi:10.1371/journal.pgen.1011058)
Supplement: S4 Fig — Single cell RNAseq data from the gEAR (http://umgear.org) was plotted for each of the 29 genes associated with Metabolic (M) or Sensory (S) hearing loss. Expression was normalised to Hprt (represented by a horizontal line at y = 1 on each plot). Marker genes included for comparison are Myo7a (hair cells), Fgf8 (inner hair cells), Slc26a5 (outer hair cells), Sox2 (non-sensory cells), S100b (inner pillar cells), Hes5 (Deiters’ cells), comparison (Kcne1 (marginal cells), Met(intermediate cells), Cldn11 (basal cells), Slc26a4 (spindle and root cells) and Gm525 (fibrocytes). (PDF) [file pgen.1011058.s011.pdf]

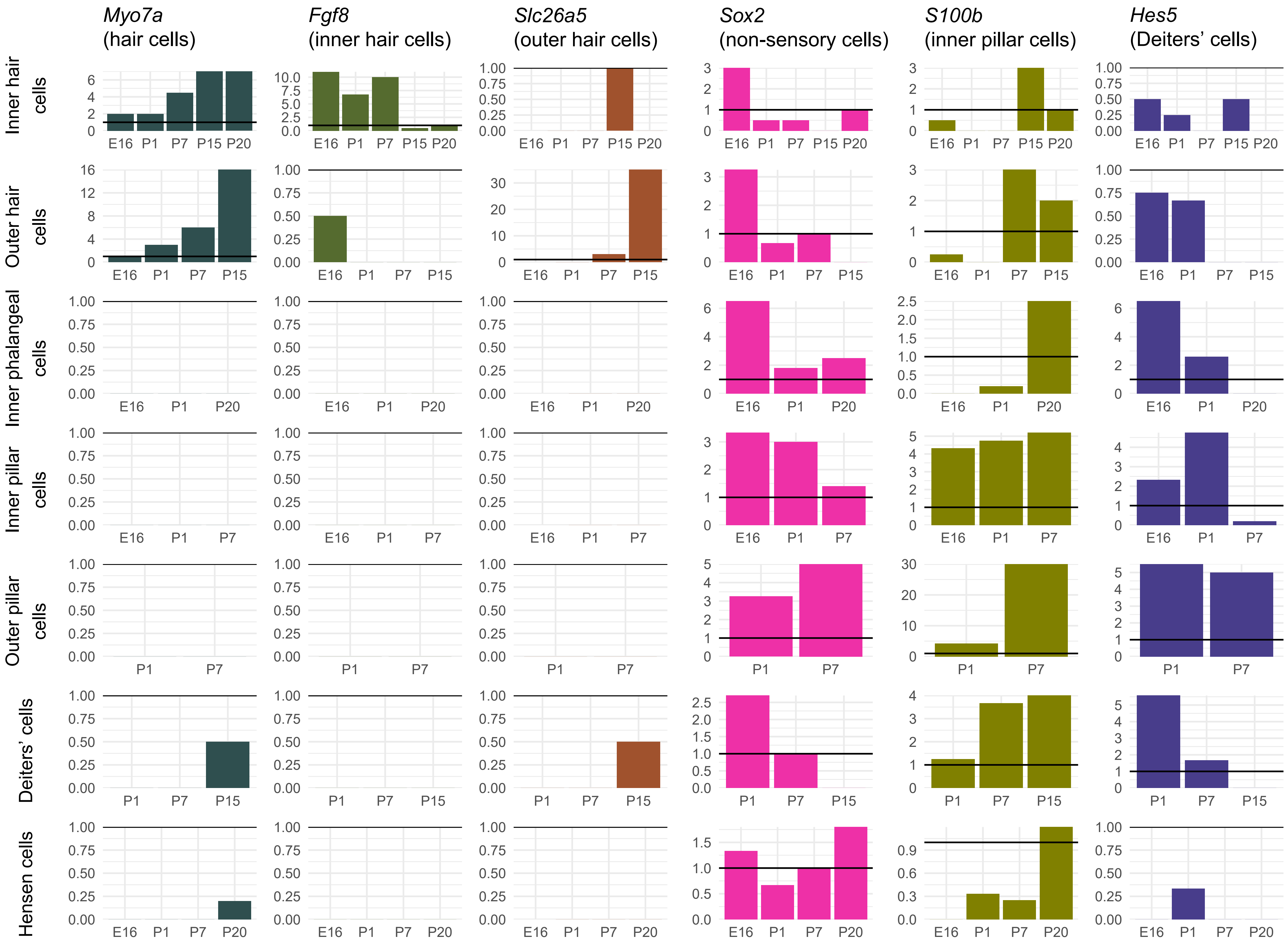
Myo7a (hair cells)
Fgf8 (inner hair cells)
Slc26a5 (outer hair cells)
Sox2 (non-sensory cells)
S100b (inner pillar cells)
Hes5 (Deiters' cells)
Inner hair cells
Outer hair cells
Inner phalangeal cells
Inner pillar cells
Outer pillar cells
Deiters' cells
Hensen cells
E16
P1
P7
P15
P20

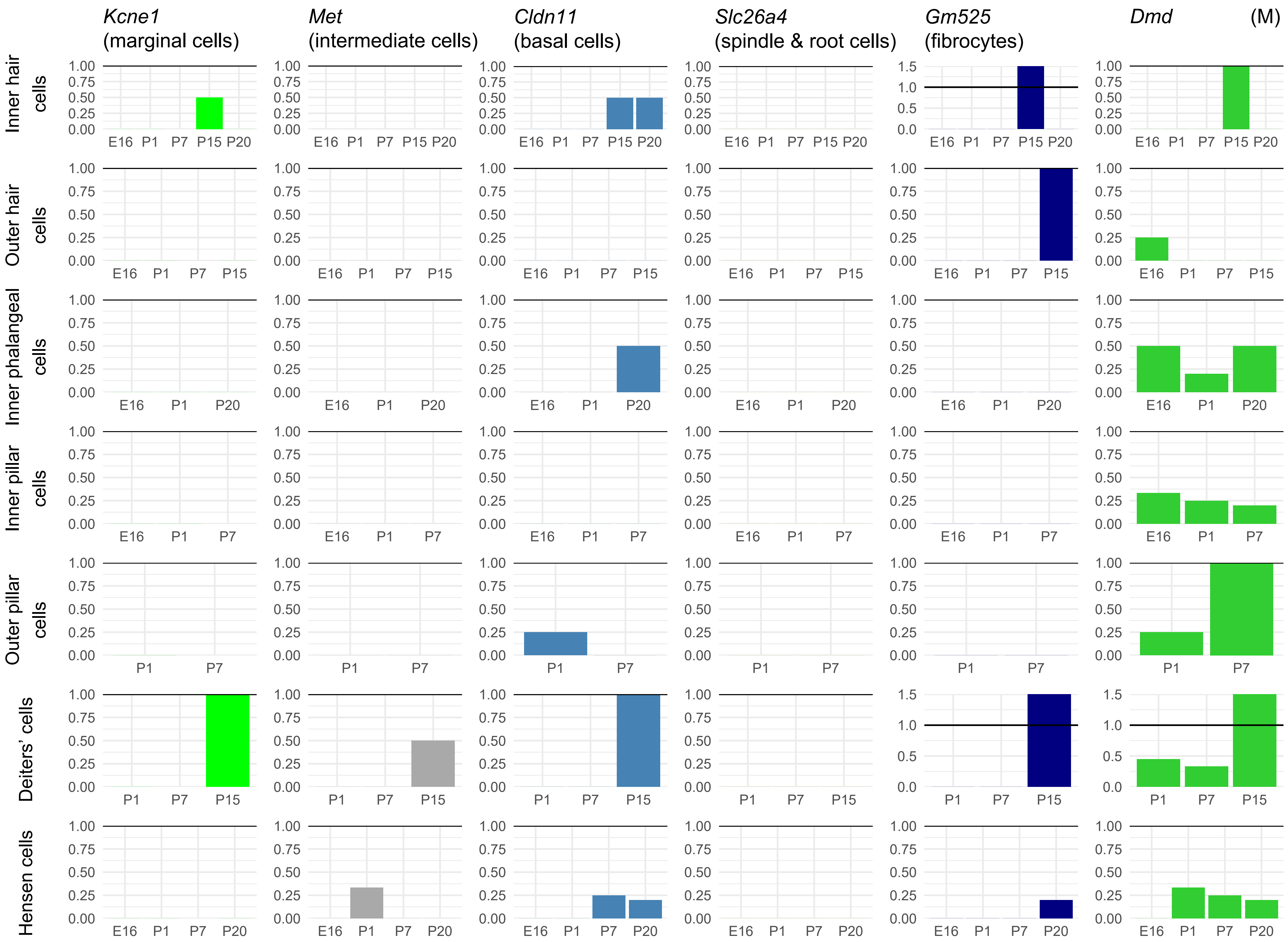

(M)
Kcne1
(marginal cells)
Met
(intermediate cells)
Cldn11
(basal cells)
Slc26a4
(spindle & root cells)
Gm525
(fibrocytes)
Dmd
Inner hair cells
Outer hair cells
Inner phalangeal cells
Inner pillar cells
Outer pillar cells
Deiters' cells
Hensen cells
E16
P1
P7
P15
P20

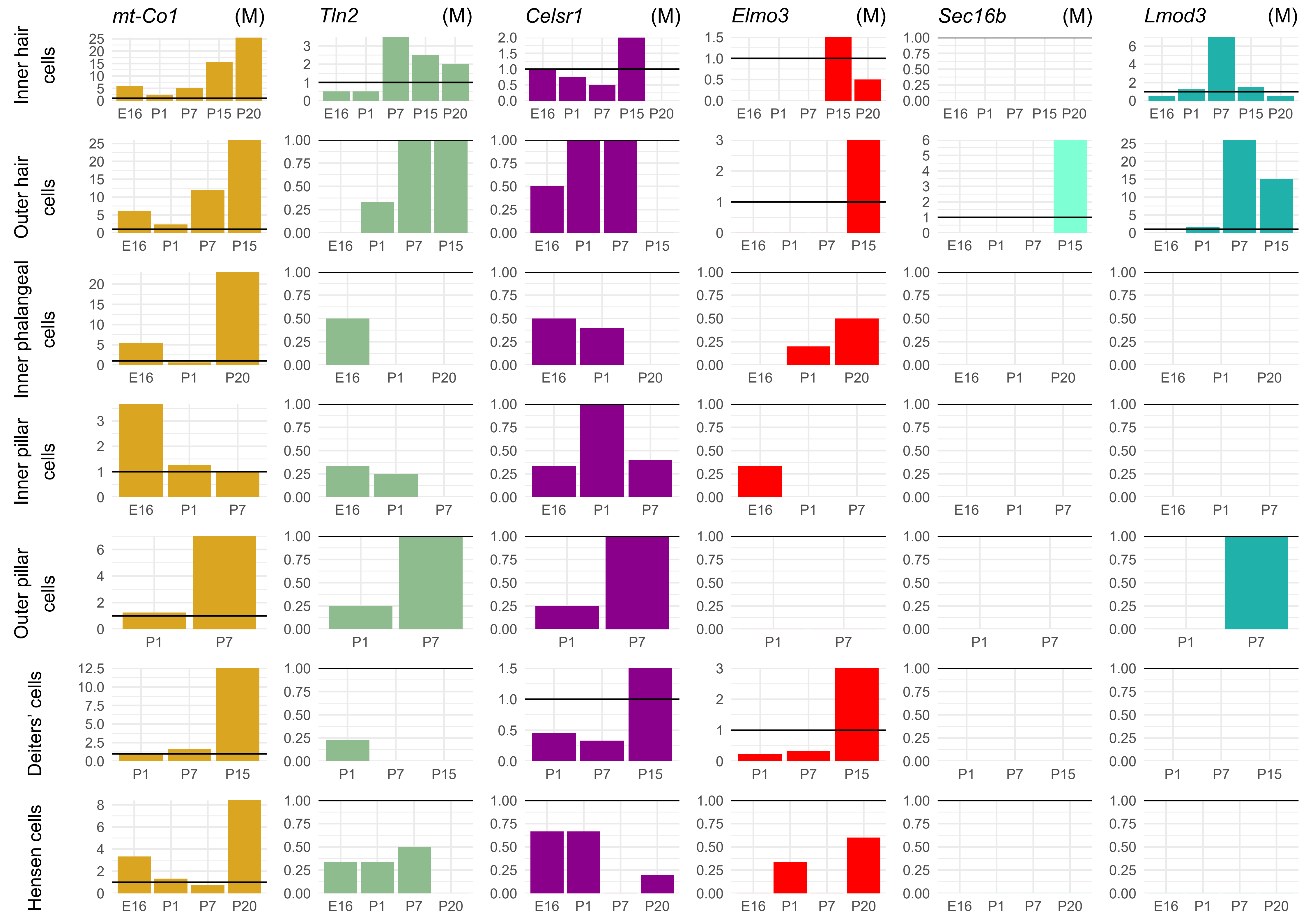

mt-Co1 (M)
Tln2 (M)
Celsr1 (M)
Elmo3 (M)
Sec16b (M)
Lmod3 (M)
Inner hair cells
Outer hair cells
Inner phalangeal cells
Inner pillar cells
Outer pillar cells
Deiters' cells
Hensen cells
E16
P1
P7
P15
P20

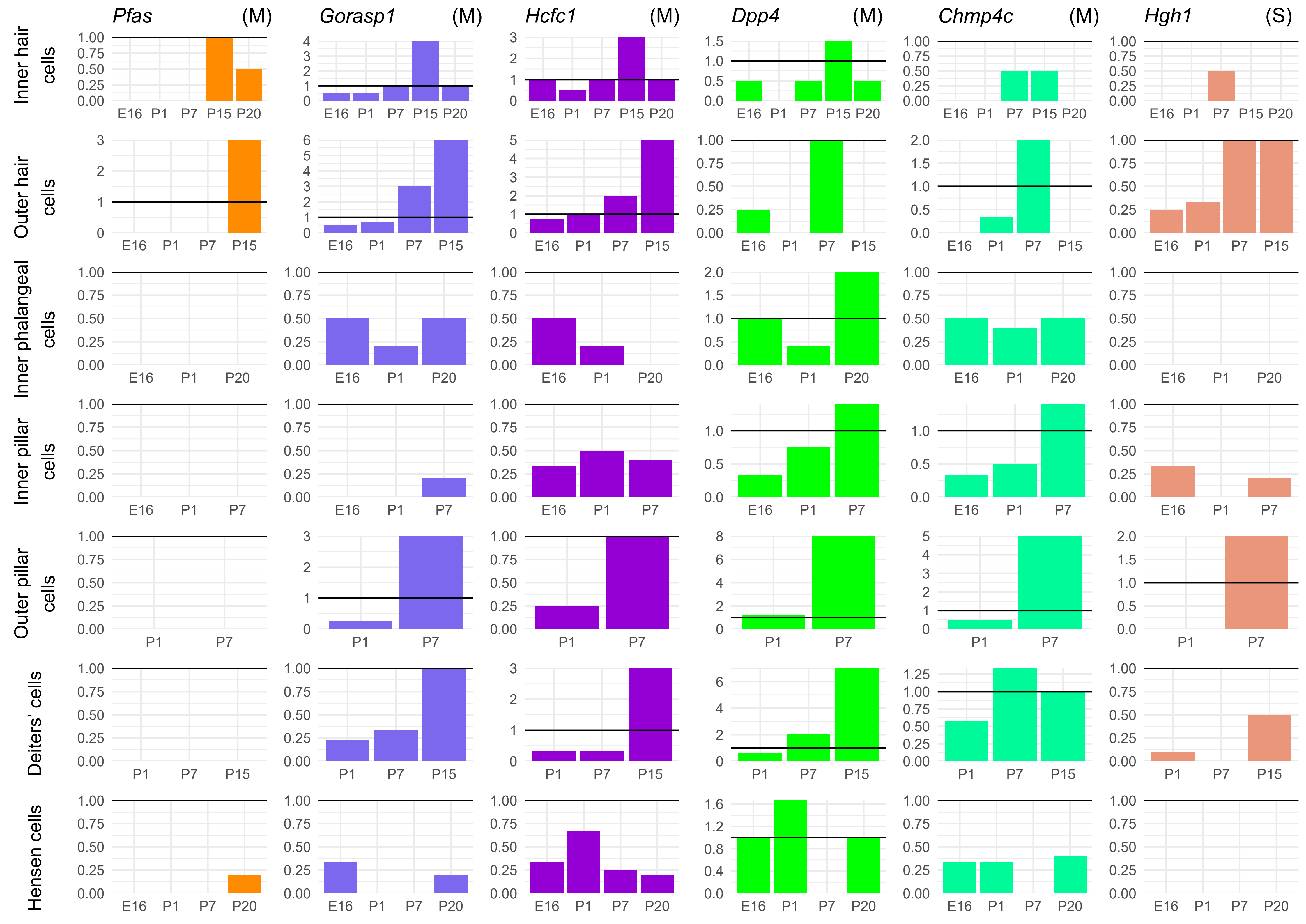

Pfas (M)
Gorasp1 (M)
Hcfc1 (M)
Dpp4 (M)
Chmp4c (M)
Hgh1 (S)
Inner hair cells
Outer hair cells
Inner phalangeal cells
Inner pillar cells
Outer pillar cells
Deiters' cells
Hensen cells
E16
P1
P7
P15
P20

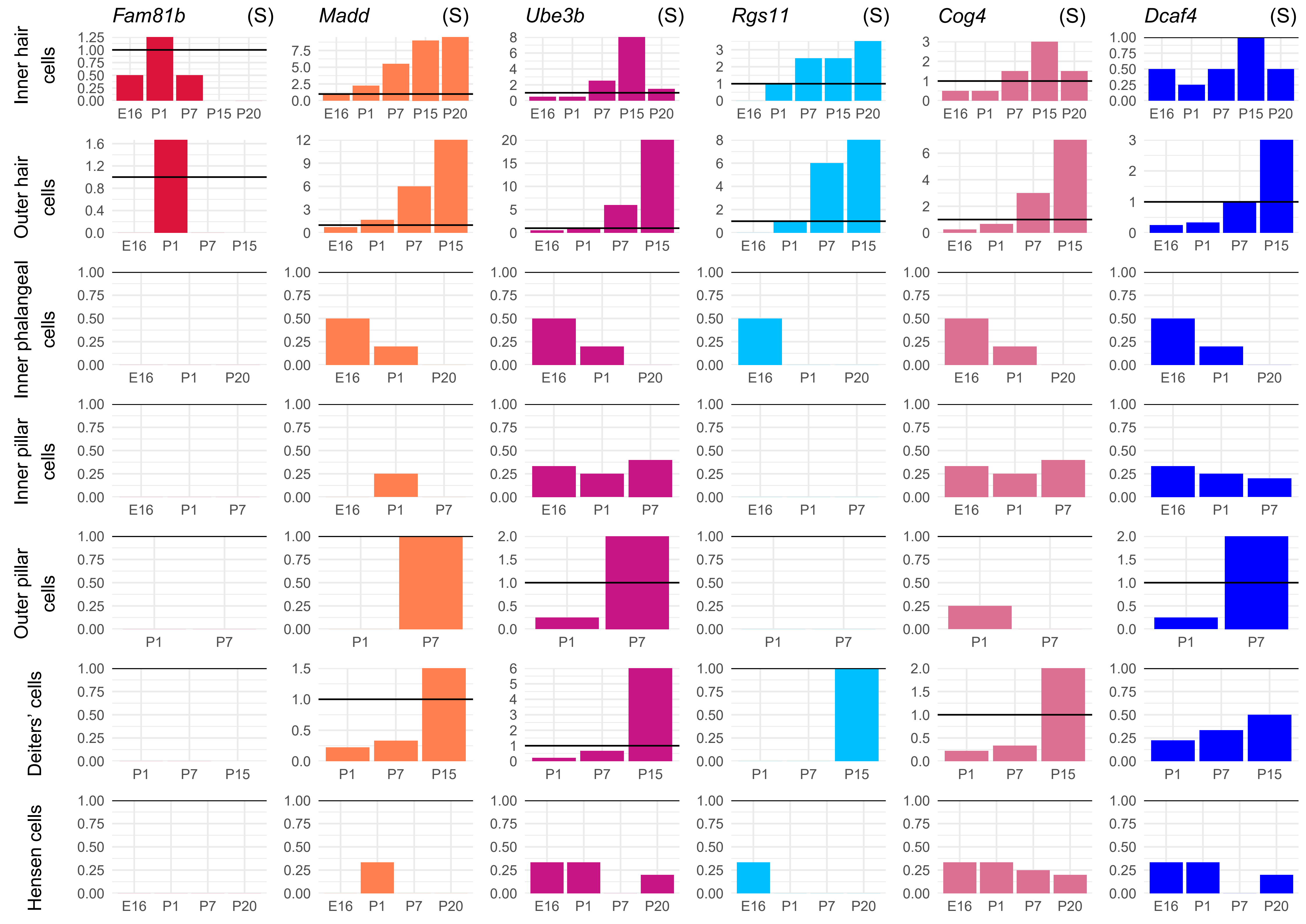

Fam81b (S)
Madd (S)
Ube3b (S)
Rgs11 (S)
Cog4 (S)
Dcaf4 (S)
Inner hair cells
Outer hair cells
Inner phalangeal cells
Inner pillar cells
Outer pillar cells
Deiters' cells
Hensen cells
E16 P1 P7 P15 P20

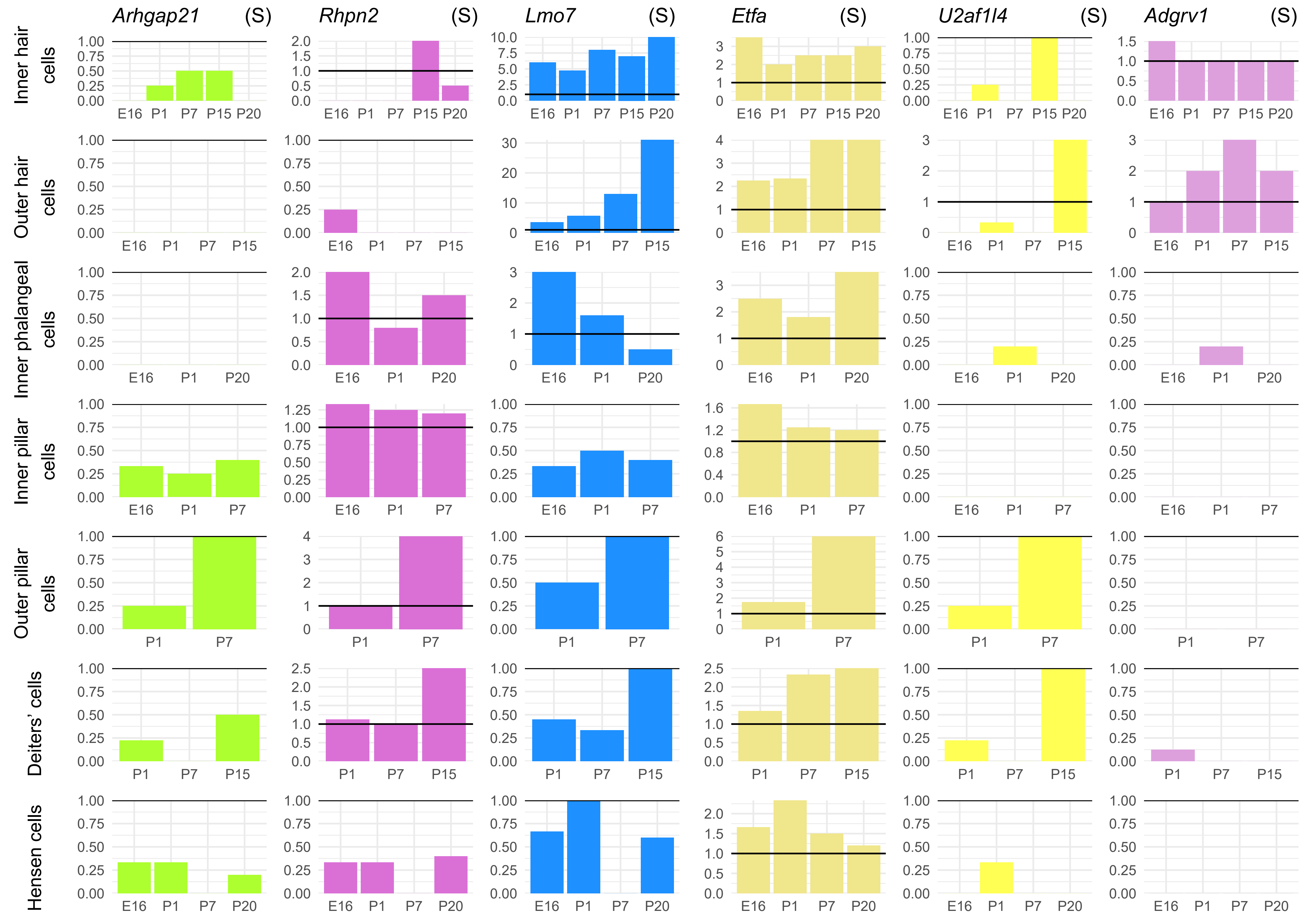
Arhgap21 (S)
Rhpn2 (S)
Lmo7 (S)
Etfa (S)
U2af1l4 (S)
Adgrv1 (S)
Inner hair cells
Outer hair cells
Inner phalangeal cells
Inner pillar cells
Outer pillar cells
Deiters' cells
Hensen cells
E16
P1
P7
P15
P20

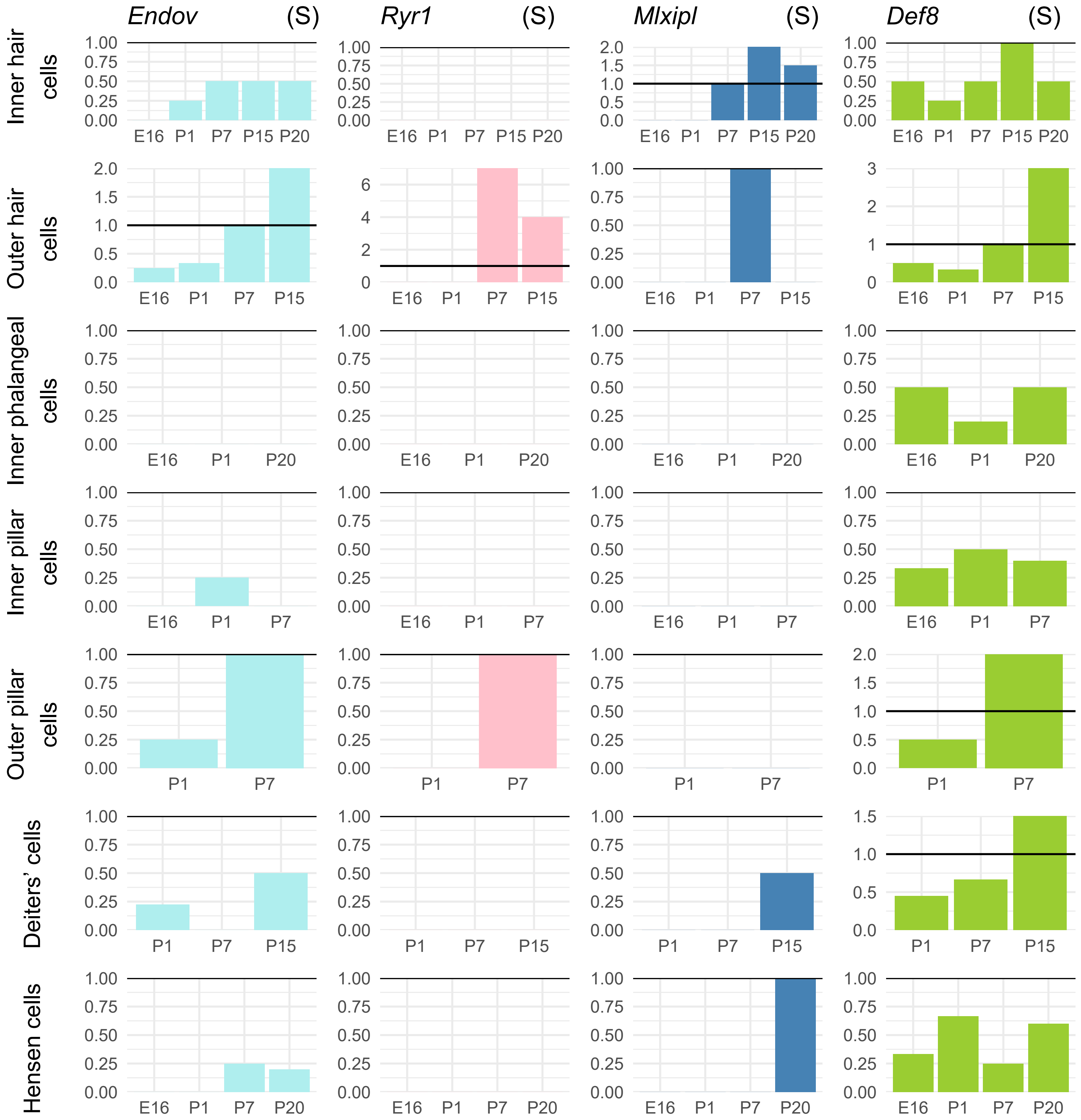

Endov (S)
Ryr1 (S)
Mlxipl (S)
Def8 (S)
Inner hair cells
Outer hair cells
Inner phalangeal cells
Inner pillar cells
Outer pillar cells
Deiters' cells
Hensen cells
E16
P1
P7
P15
P20

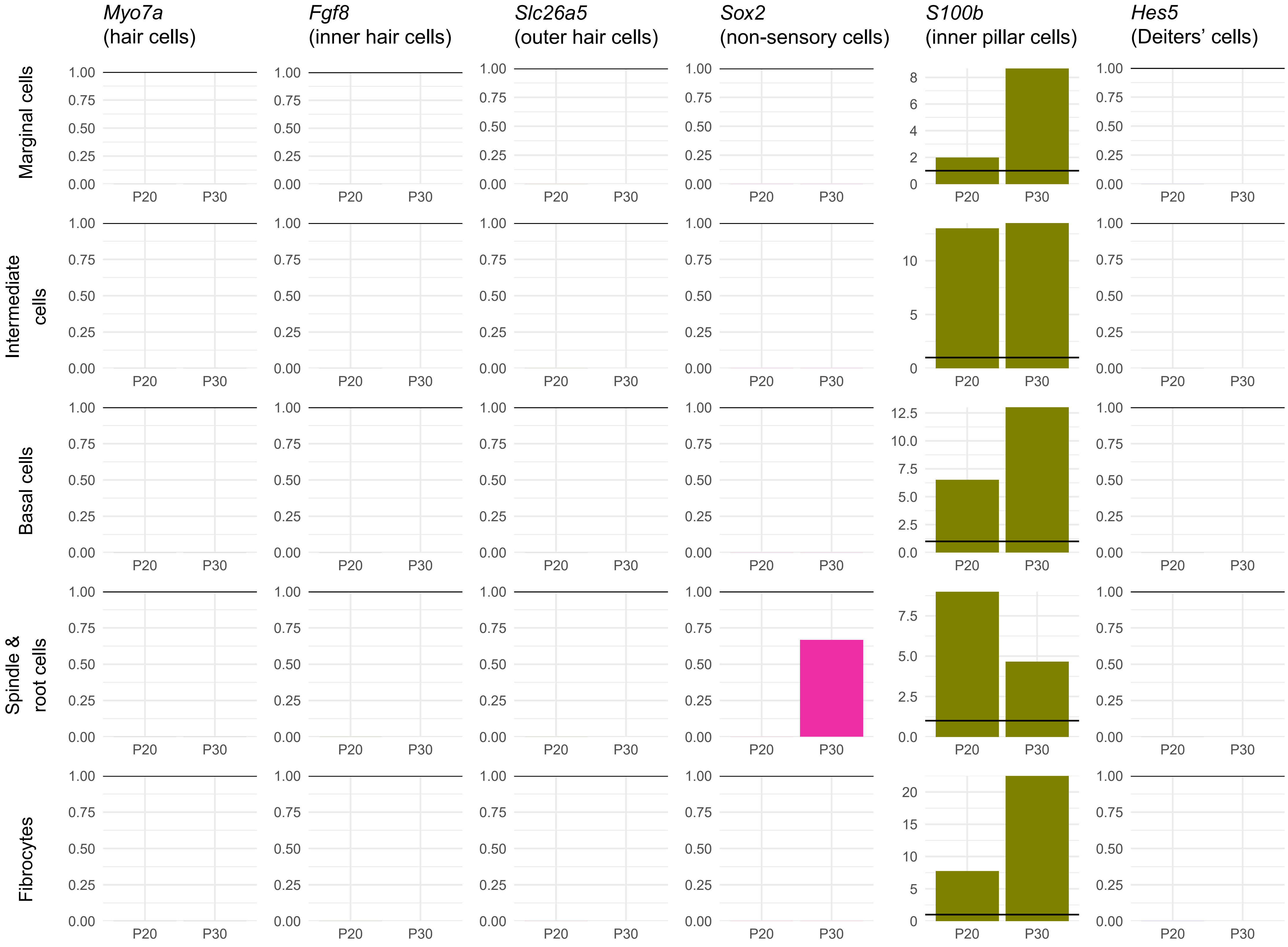

Myo7a
(hair cells)
Fgf8
(inner hair cells)
Slc26a5
(outer hair cells)
Sox2
(non-sensory cells)
S100b
(inner pillar cells)
Hes5
(Deiters' cells)
Marginal cells
Intermediate cells
Basal cells
Spindle & root cells
Fibrocytes
P20
P30

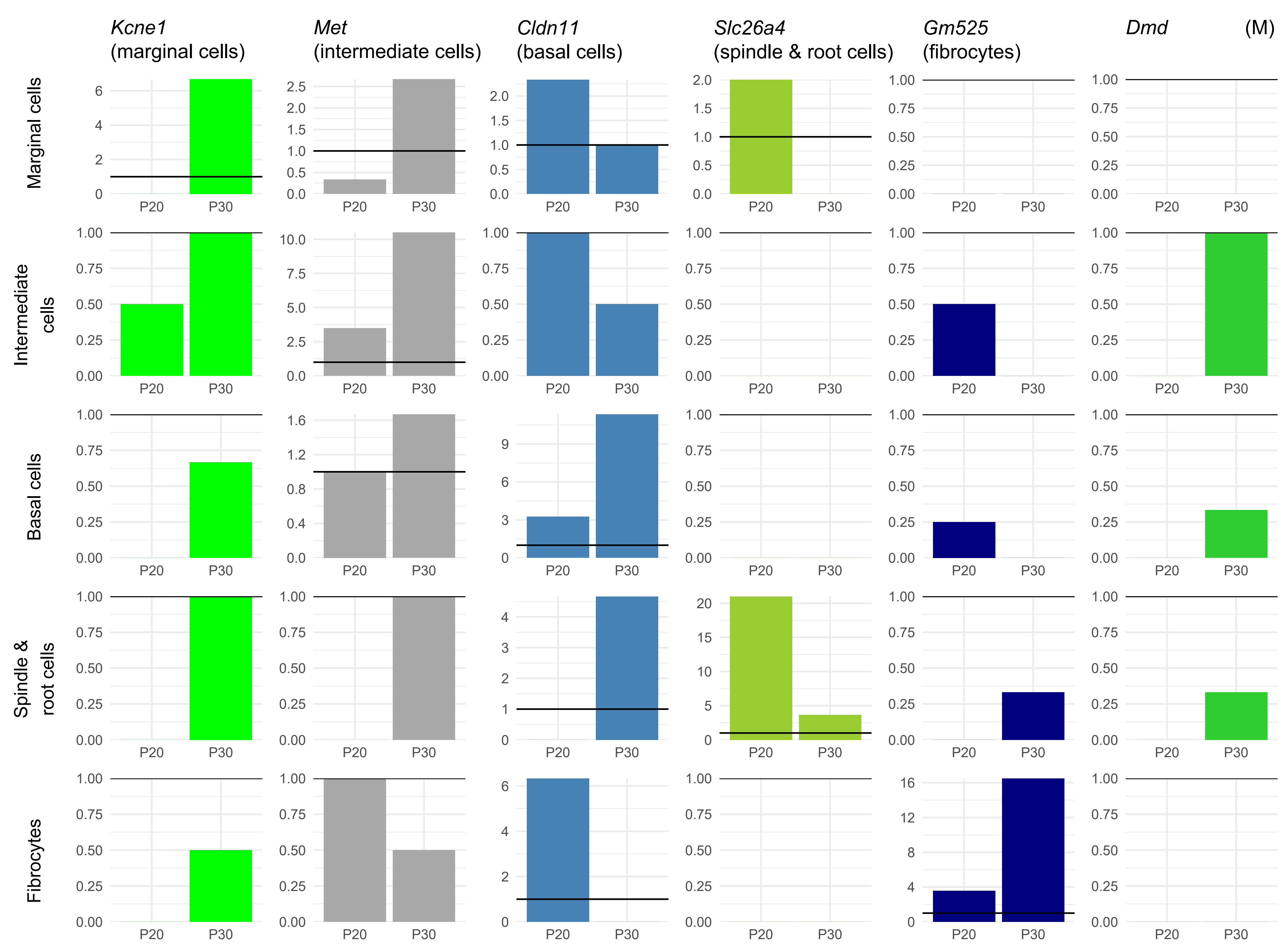

(M)
Kcne1 (marginal cells)
Met (intermediate cells)
Cldn11 (basal cells)
Slc26a4 (spindle & root cells)
Gm525 (fibrocytes)
Dmd
Marginal cells
Intermediate cells
Basal cells
Spindle & root cells
Fibrocytes
P20
P30

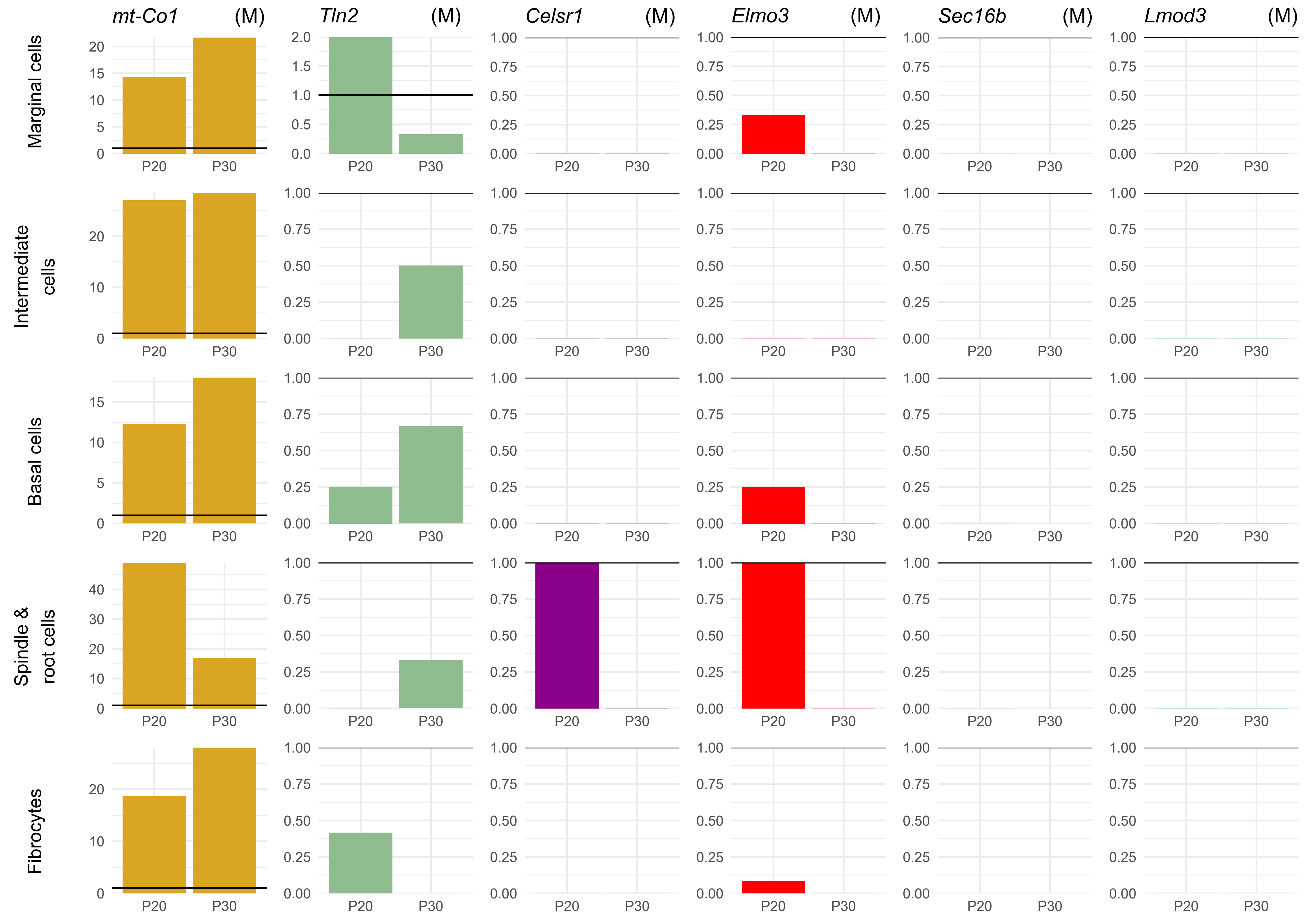

mt-Co1 (M)
Tln2 (M)
Celsr1 (M)
Elmo3 (M)
Sec16b (M)
Lmod3 (M)
Marginal cells
Intermediate cells
Basal cells
Spindle & root cells
Fibrocytes
P20
P30

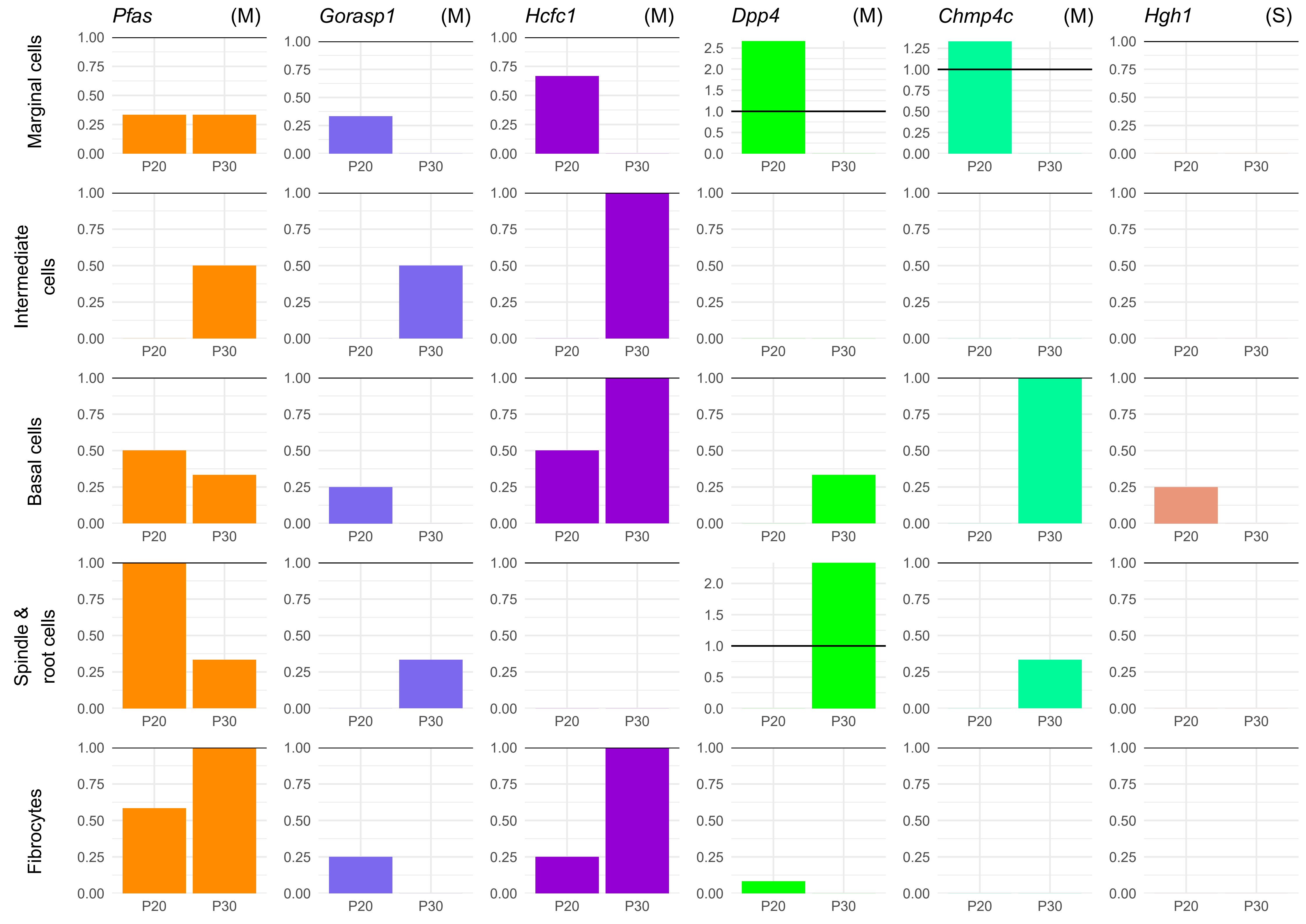

Pfas (M)
Gorasp1 (M)
Hcfc1 (M)
Dpp4 (M)
Chmp4c (M)
Hgh1 (S)
Marginal cells
Intermediate cells
Basal cells
Spindle & root cells
Fibrocytes
P20
P30

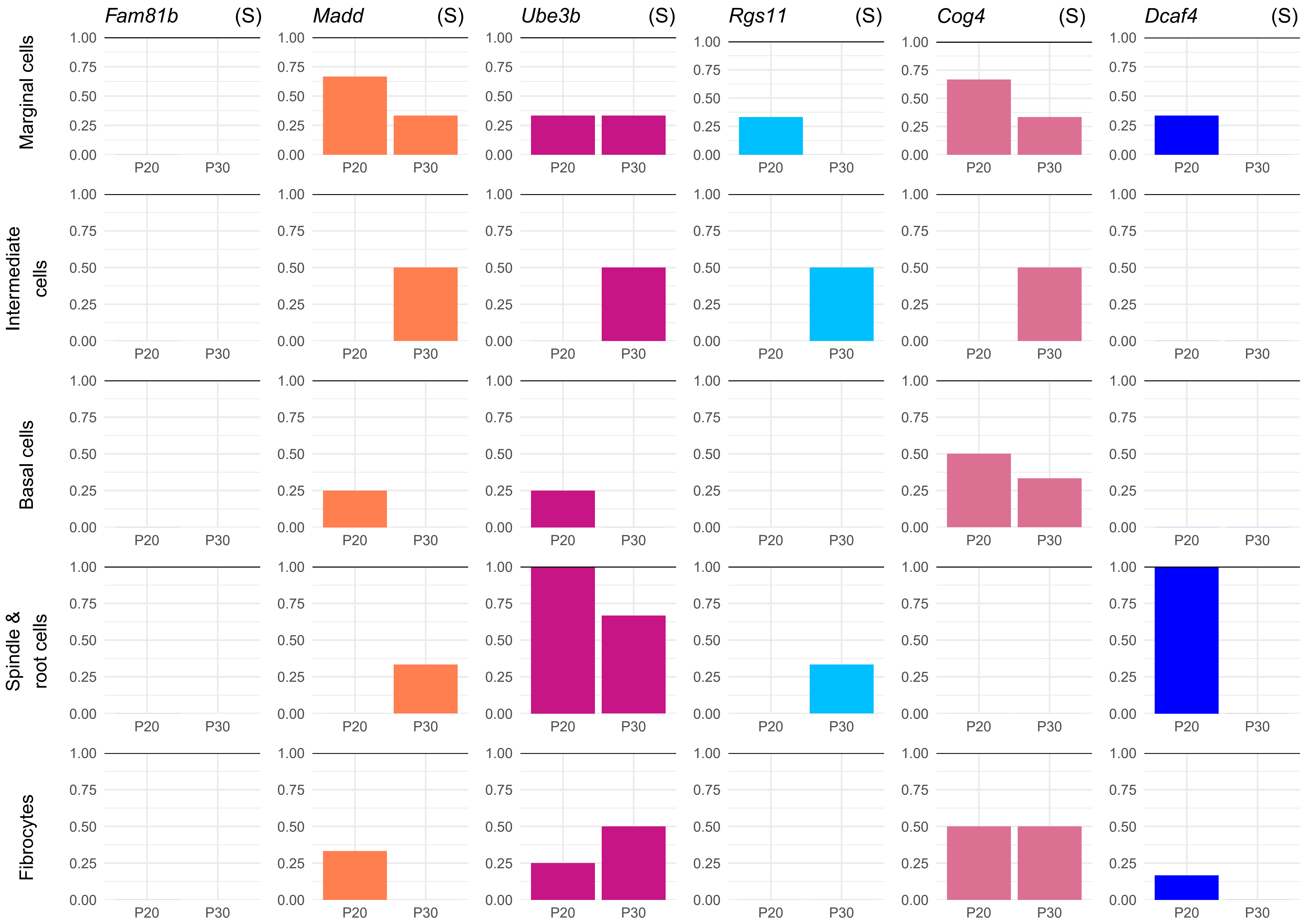

Fam81b (S)
Madd (S)
Ube3b (S)
Rgs11 (S)
Cog4 (S)
Dcaf4 (S)
Marginal cells
Intermediate cells
Basal cells
Spindle & root cells
Fibrocytes
1.00
0.75
0.50
0.25
0.00
P20
P30

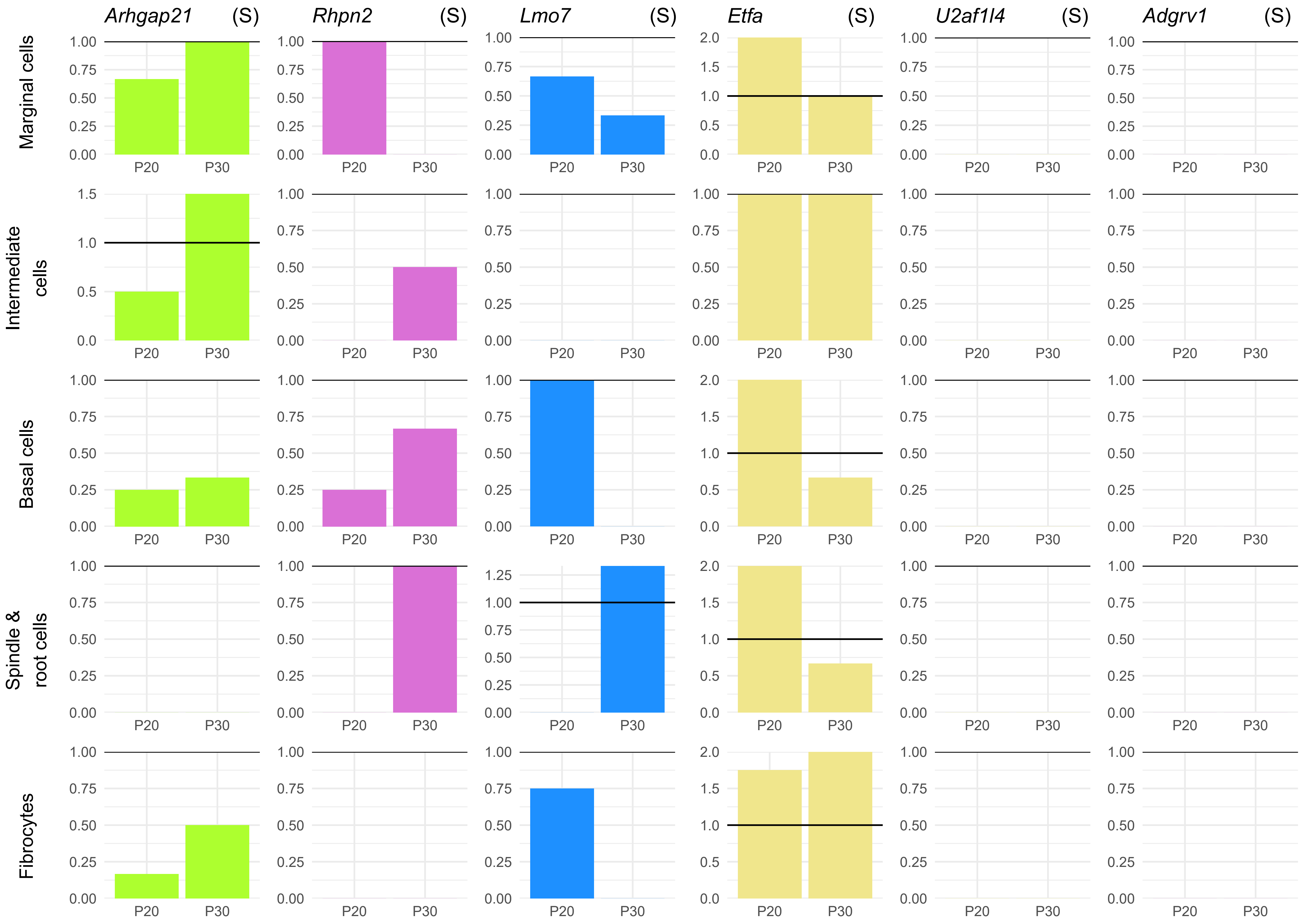

Arhgap21 (S)
Rhpn2 (S)
Lmo7 (S)
Etfa (S)
U2af1l4 (S)
Adgrv1 (S)
Marginal cells
Intermediate cells
Basal cells
Spindle & root cells
Fibrocytes
P20
P30

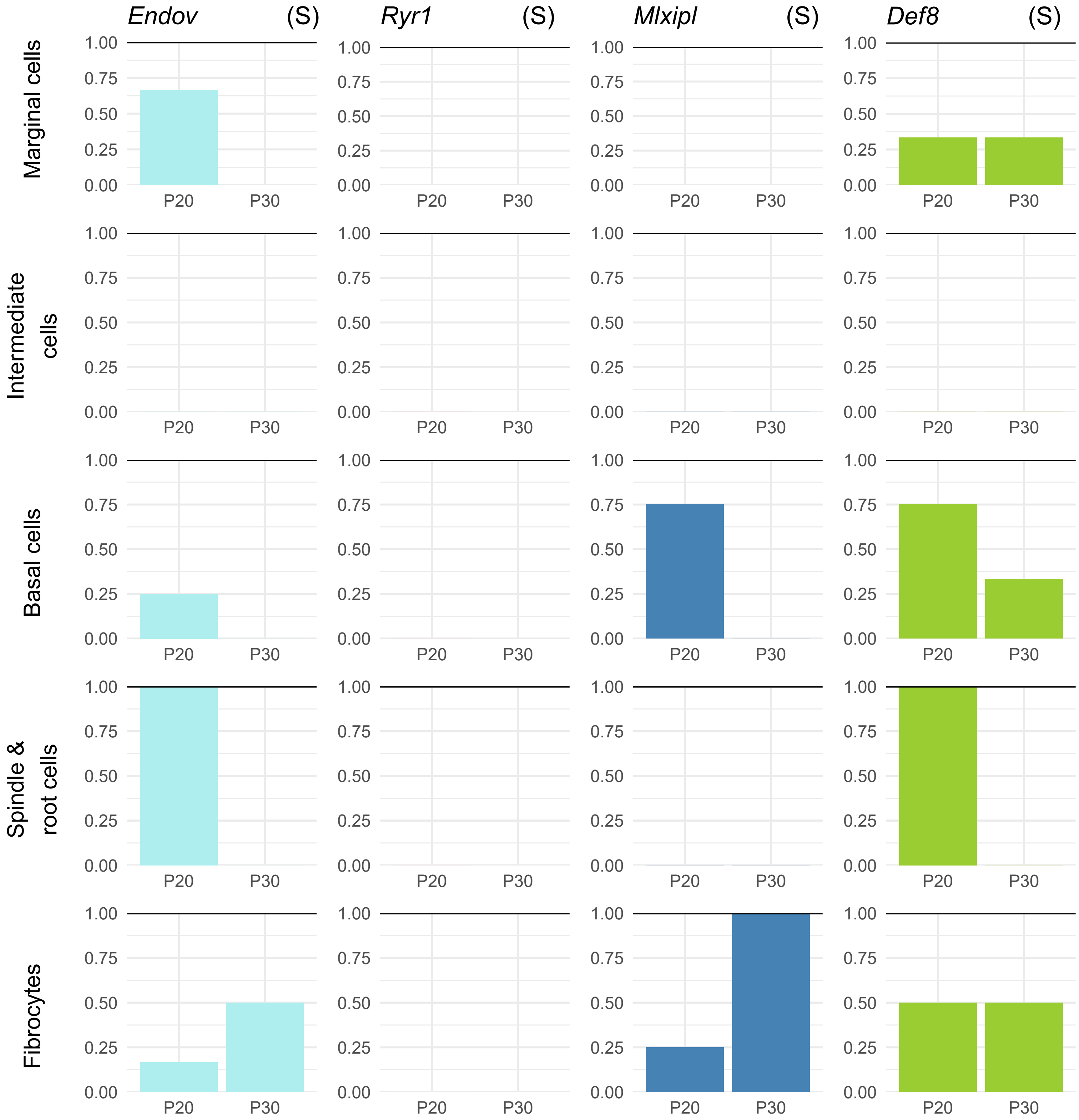
Endov (S)
Ryr1 (S)
Mlxipl (S)
Def8 (S)
Marginal cells
Intermediate cells
Basal cells
Spindle & root cells
Fibrocytes
1.00
0.75
0.50
0.25
0.00
P20
P30

S4 Fig. Expression levels at different developmental stages of genes linked to specific subtypes of hearing loss. Single cell RNAseq data from the gEAR (http://umgear.org) was plotted for each of the 29 genes associated with Metabolic (M) or Sensory (S) hearing loss. Expression was normalised to *Hprt* (represented by a horizontal line at y=1 on each plot). Marker genes included for comparison are *Myo7a* (hair cells), *Fgf8* (inner hair cells), *Slc26a5* (outer hair cells), *Sox2* (non-sensory cells), *S100b* (inner pillar cells), *Hes5* (Deiters' cells), comparison (*Kcne1* (marginal cells), *Met*(intermediate cells), *Cldn11* (basal cells), *Slc26a4* (spindle and root cells) and *Gm525* (fibrocytes).
