# Supplementary material for: Accurate phenotypic classification and exome sequencing allow identification of novel genes and variants associated with adult-onset hearing loss
Source: PLoS Genet. 2023 Nov 27;19(11):e1011058. doi: 10.1371/journal.pgen.1011058 (PMC10718637; doi:10.1371/journal.pgen.1011058)
Supplement: S5 Fig — Images of the cochlear duct (basal turn) at ages from E14.5 (where no staining is visible) to P4. At least three mice were examined at each age. Brown shows where Madd is present (visible from E16.5 and older); hair cells are marked with arrowheads. Scale bar = 20μm. (PDF) [file pgen.1011058.s012.pdf]

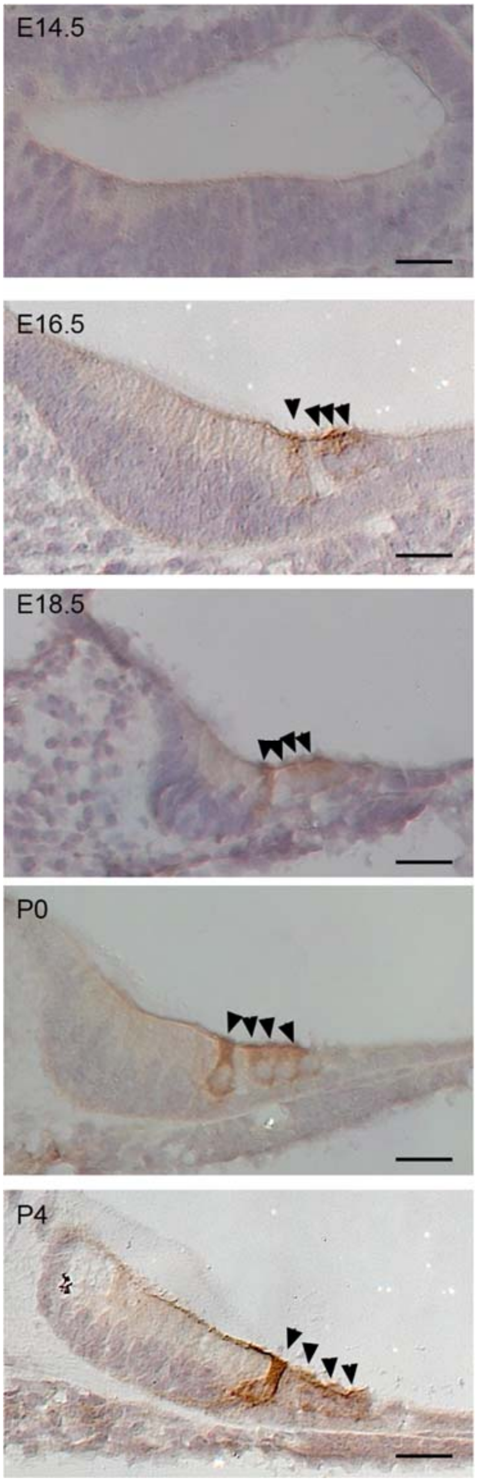


**S5 Fig.** Madd is expressed in the hair cells of the mouse inner ear. Images of the cochlear duct (basal turn) at ages from E14.5 (where no staining is visible) to P4. At least three mice were examined at each age. Brown shows where Madd is present (visible from E16.5 and older); hair cells are marked with arrowheads. Scale bar = 20µm.
