# Supplementary material for: Accurate phenotypic classification and exome sequencing allow identification of novel genes and variants associated with adult-onset hearing loss
Source: PLoS Genet. 2023 Nov 27;19(11):e1011058. doi: 10.1371/journal.pgen.1011058 (PMC10718637; doi:10.1371/journal.pgen.1011058)
Supplement: S6 Fig — For the 41 variants identified in the MUSC cohort, audiograms of TwinsUK carriers are shown on the right where available. The variant in HADH was identified in both cohorts. For the remaining 3 variants identified in the TwinsUK cohort, the audiograms of MUSC carriers are shown on the left. Two audiograms are shown for each variant in each cohort; the thresholds from the left ear are shown on the left, and those from the right ear on the right. Numbers and average ages of each group are listed on the graph. The symbols at the top of each graph mark which groups passed the criteria for each stimulus frequency compared to the relevant reference group (+ for male, = for female, and * for all participants). Error bars are standard deviation. (PDF) [file pgen.1011058.s013.pdf]

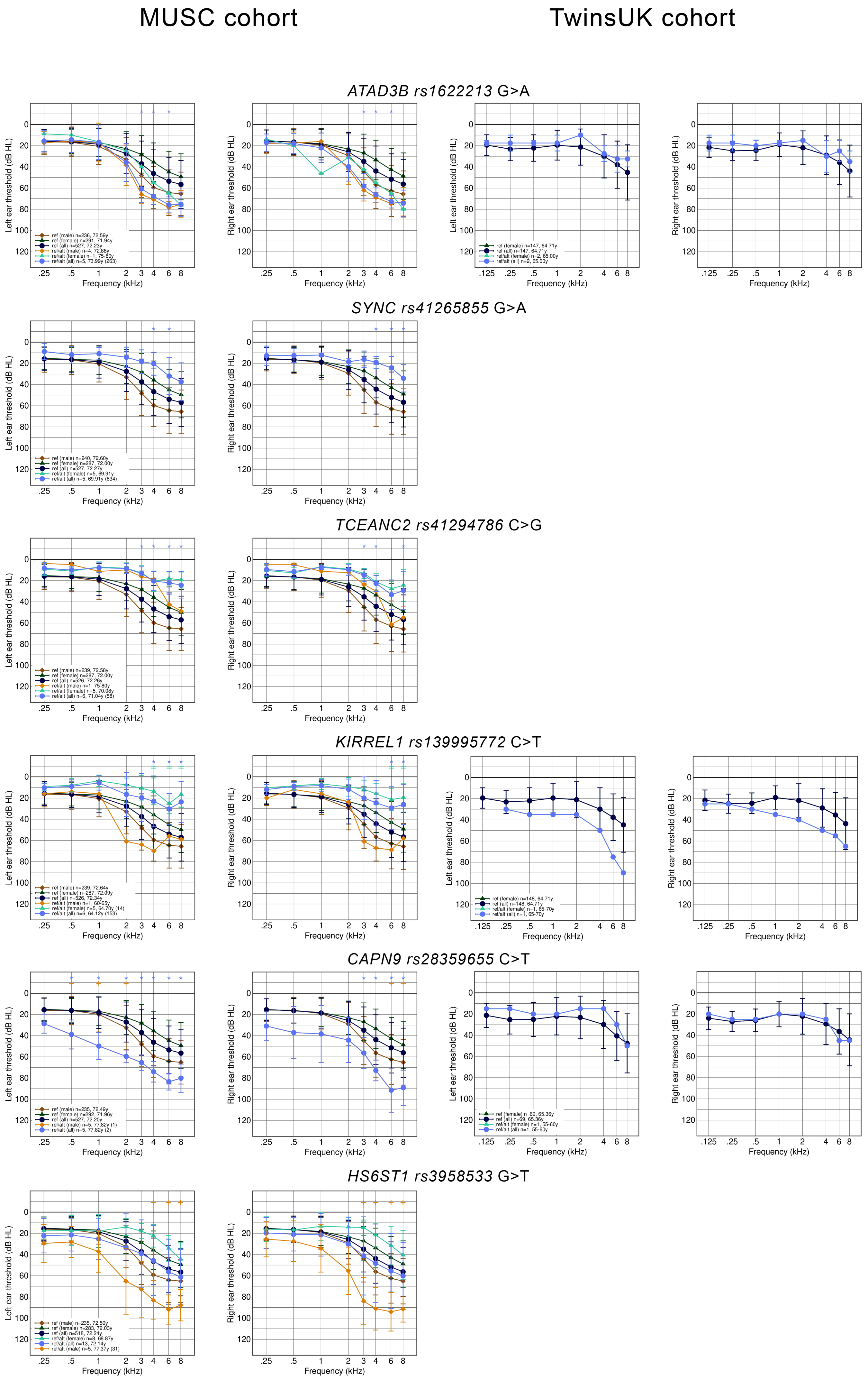
MUSC cohort
TwinsUK cohort
ATAD3B rs1622213 G>A
ref (male) n=236, 72.59y
ref (female) n=291, 71.94y
ref (all) n=527, 72.23y
ref/alt (male) n=4, 72.88y
ref/alt (female) n=1, 75-80y
ref/alt (all) n=5, 73.99y (263)
ref (female) n=147, 64.71y
ref (all) n=147, 64.71y
ref/alt (female) n=2, 65.00y
ref/alt (all) n=2, 65.00y
SYNC rs41265855 G>A
ref (male) n=240, 72.60y
ref (female) n=287, 72.00y
ref (all) n=527, 72.27y
ref/alt (female) n=5, 69.91y
ref/alt (all) n=5, 69.91y (634)
TCEANC2 rs41294786 C>G
ref (male) n=239, 72.58y
ref (female) n=287, 72.00y
ref (all) n=526, 72.26y
ref/alt (male) n=1, 75-80y
ref/alt (female) n=5, 70.08y
ref/alt (all) n=6, 71.04y (58)
KIRREL1 rs139995772 C>T
ref (male) n=239, 72.64y
ref (female) n=287, 72.09y
ref (all) n=526, 72.34y
ref/alt (male) n=1, 60-65y
ref/alt (female) n=5, 64.70y (14)
ref/alt (all) n=6, 64.12y (153)
ref (female) n=148, 64.71y
ref (all) n=148, 64.71y
ref/alt (female) n=1, 65-70y
ref/alt (all) n=1, 65-70y
CAPN9 rs28359655 C>T
ref (male) n=235, 72.49y
ref (female) n=292, 71.96y
ref (all) n=527, 72.20y
ref/alt (male) n=5, 77.82y (1)
ref/alt (all) n=5, 77.82y (2)
ref (female) n=69, 65.36y
ref (all) n=69, 65.36y
ref/alt (female) n=1, 55-60y
ref/alt (all) n=1, 55-60y
HS6ST1 rs3958533 G>T
ref (male) n=235, 72.50y
ref (female) n=283, 72.03y
ref (all) n=518, 72.24y
ref/alt (female) n=8, 68.87y
ref/alt (all) n=13, 72.14y
ref/alt (male) n=5, 77.37y (31)
Left ear threshold (dB HL)
Right ear threshold (dB HL)
Frequency (kHz)
0
20
40
60
80
100
120
.125
.25
.5
1
2
3
4
6
8

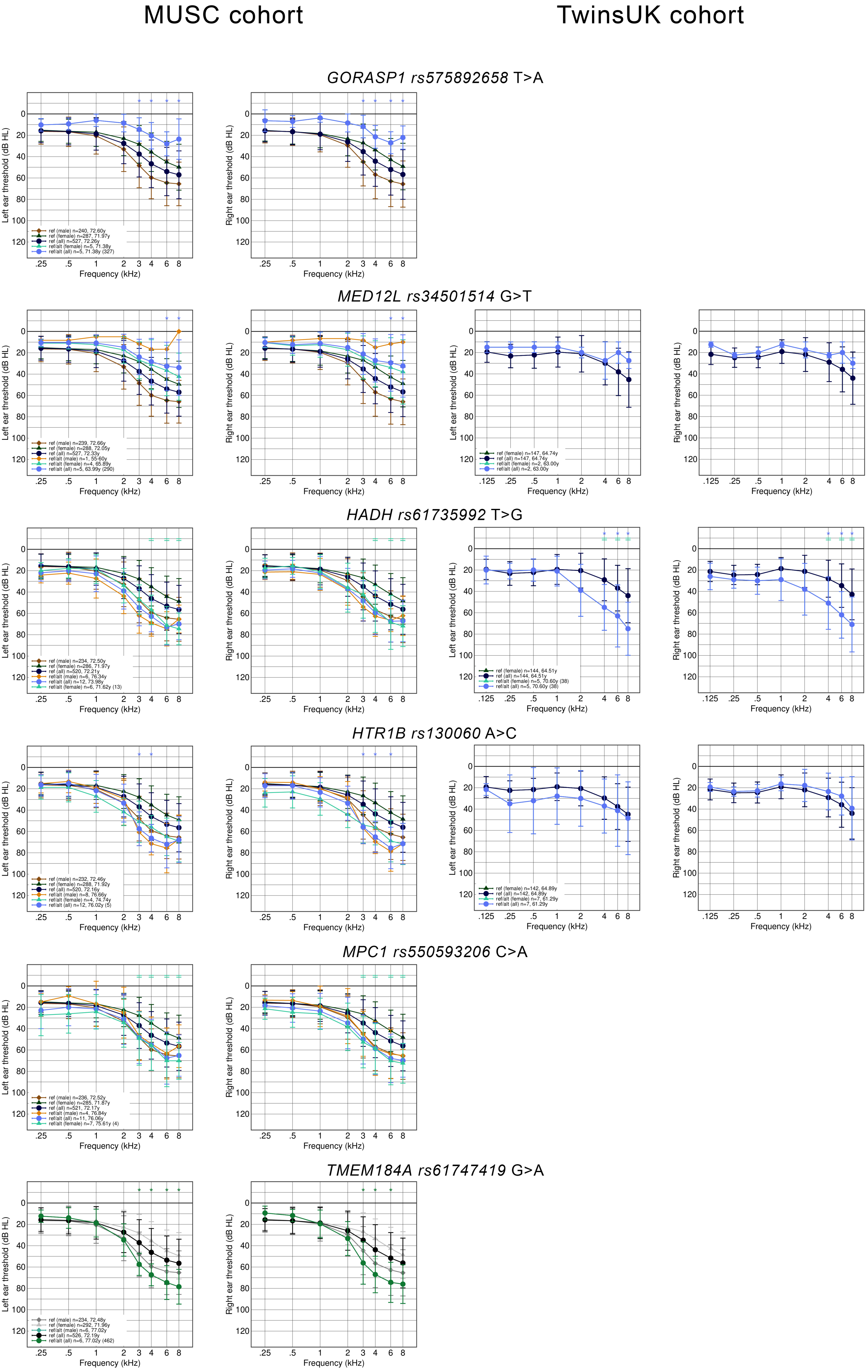

MUSC cohort
TwinsUK cohort
GORASP1 rs575892658 T>A
ref (male) n=240, 72.60y
ref (female) n=287, 71.97y
ref (all) n=527, 72.26y
ref/alt (female) n=5, 71.38y
ref/alt (all) n=5, 71.38y (327)
MED12L rs34501514 G>T
ref (male) n=239, 72.66y
ref (female) n=288, 72.05y
ref (all) n=527, 72.33y
ref/alt (male) n=1, 55.60y
ref/alt (female) n=4, 65.89y
ref/alt (all) n=5, 63.99y (290)
ref (female) n=147, 64.74y
ref (all) n=147, 64.74y
ref/alt (female) n=2, 63.00y
ref/alt (all) n=2, 63.00y
HADH rs61735992 T>G
ref (male) n=234, 72.50y
ref (female) n=286, 71.97y
ref (all) n=520, 72.21y
ref/alt (male) n=6, 76.34y
ref/alt (all) n=12, 73.98y
ref/alt (female) n=6, 71.62y (13)
ref (female) n=144, 64.51y
ref (all) n=144, 64.51y
ref/alt (female) n=5, 70.60y (38)
ref/alt (all) n=5, 70.60y (38)
HTR1B rs130060 A>C
ref (male) n=232, 72.46y
ref (female) n=288, 71.92y
ref (all) n=520, 72.16y
ref/alt (male) n=8, 76.66y
ref/alt (female) n=4, 74.74y
ref/alt (all) n=12, 76.02y (5)
ref (female) n=142, 64.89y
ref (all) n=142, 64.89y
ref/alt (female) n=7, 61.29y
ref/alt (all) n=7, 61.29y
MPC1 rs550593206 C>A
ref (male) n=236, 72.52y
ref (female) n=285, 71.87y
ref (all) n=521, 72.17y
ref/alt (male) n=4, 76.84y
ref/alt (all) n=11, 76.06y
ref/alt (female) n=7, 75.61y (4)
TMEM184A rs61747419 G>A
ref (male) n=234, 72.48y
ref (female) n=292, 71.96y
ref/alt (male) n=6, 77.02y
ref (all) n=526, 72.19y
ref/alt (all) n=6, 77.02y (462)
Left ear threshold (dB HL)
Right ear threshold (dB HL)
Frequency (kHz)

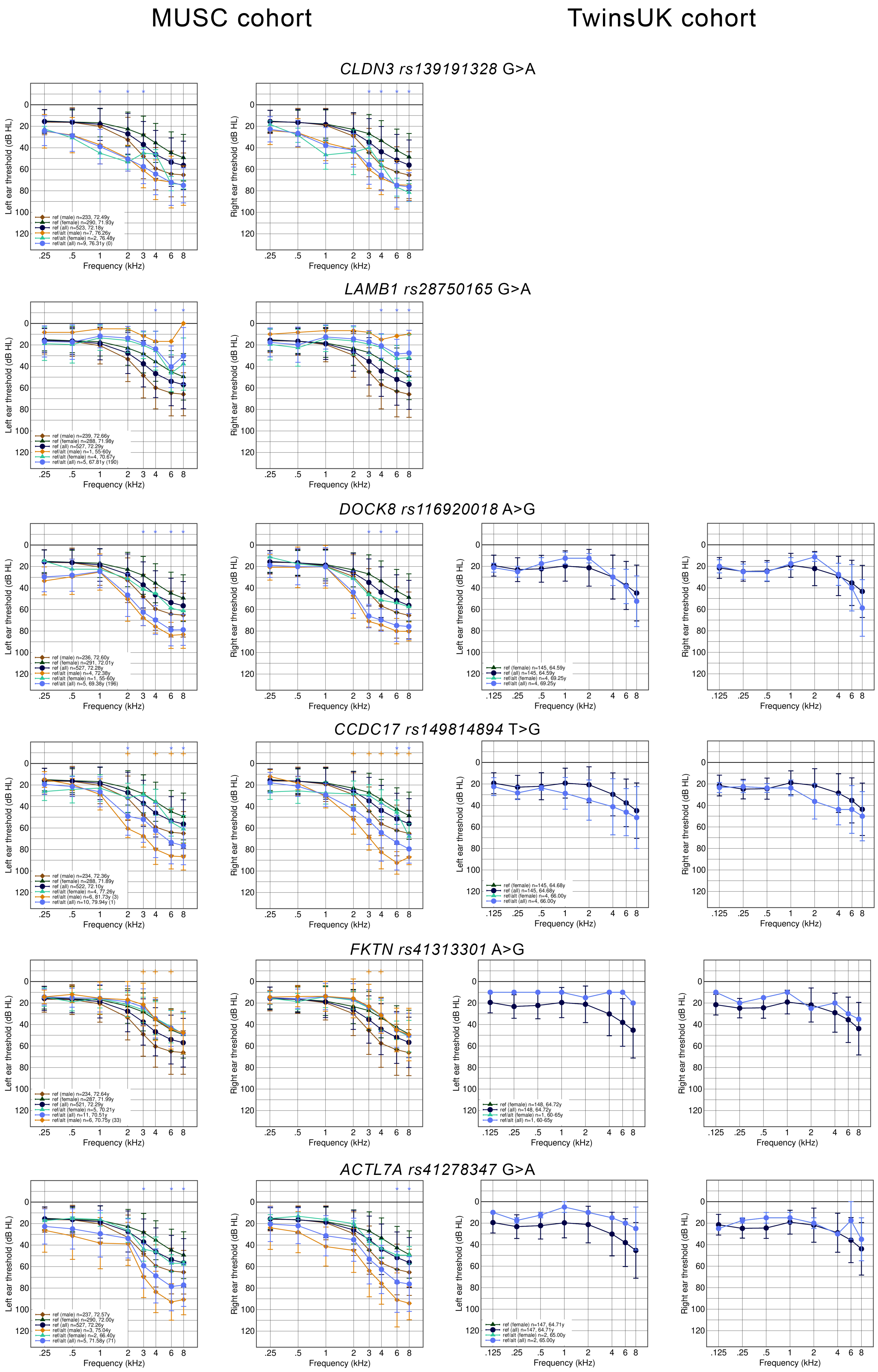

MUSC cohort
TwinsUK cohort
CLDN3 rs139191328 G>A
LAMB1 rs28750165 G>A
DOCK8 rs116920018 A>G
CCDC17 rs149814894 T>G
FKTN rs41313301 A>G
ACTL7A rs41278347 G>A
Left ear threshold (dB HL)
Right ear threshold (dB HL)
Frequency (kHz)

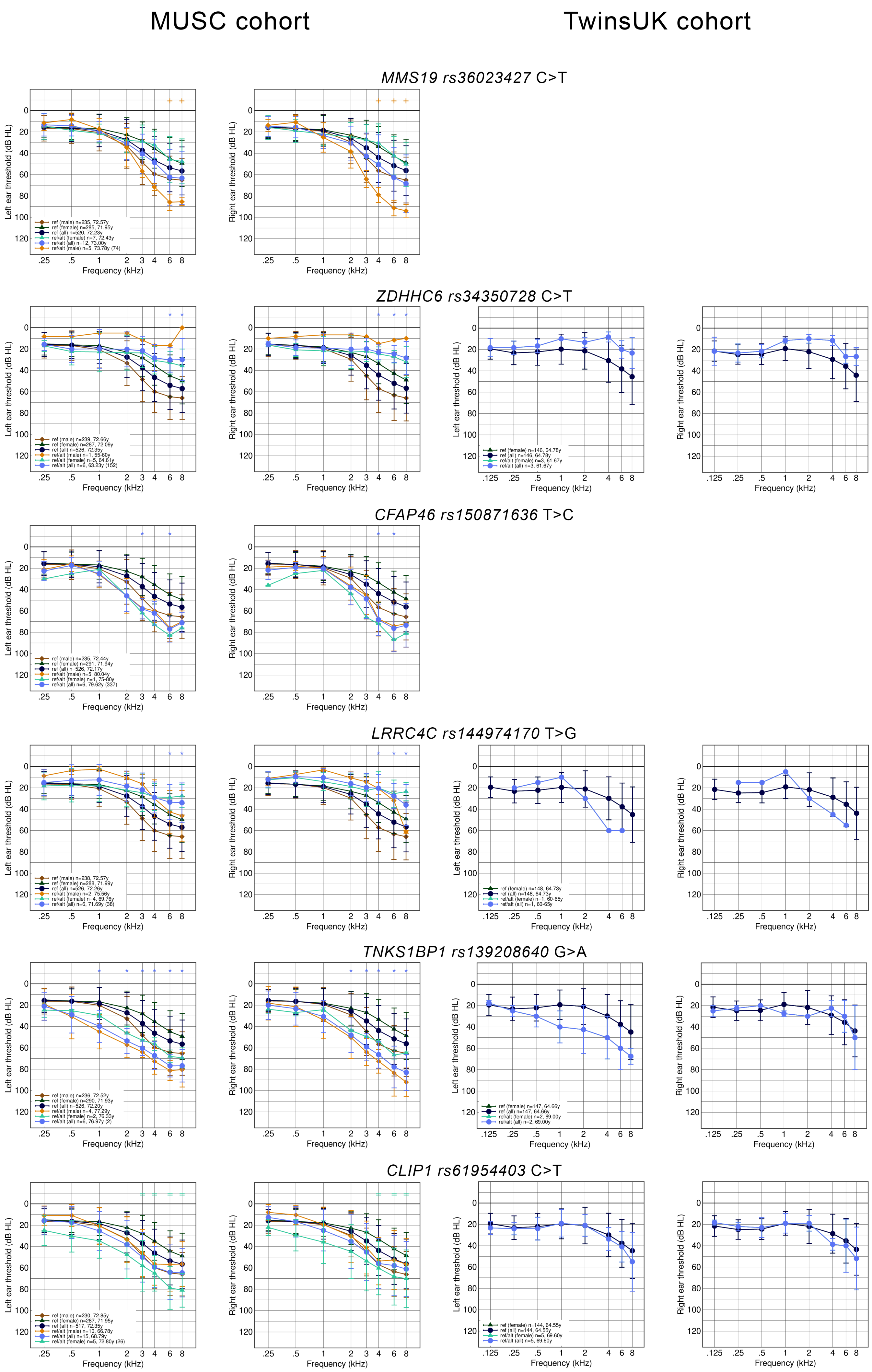
MUSC cohort
TwinsUK cohort
MMS19 rs36023427 C>T
ZDHHC6 rs34350728 C>T
CFAP46 rs150871636 T>C
LRRC4C rs144974170 T>G
TNKS1BP1 rs139208640 G>A
CLIP1 rs61954403 C>T
Left ear threshold (dB HL)
Right ear threshold (dB HL)
Frequency (kHz)
ref (male) n=235, 72.57y
ref (female) n=285, 71.95y
ref (all) n=520, 72.23y
ref/alt (female) n=7, 72.43y
ref/alt (all) n=12, 73.00y
ref/alt (male) n=5, 73.78y (74)
ref (male) n=239, 72.66y
ref (female) n=287, 72.09y
ref (all) n=526, 72.35y
ref/alt (male) n=1, 55-60y
ref/alt (female) n=5, 64.61y
ref/alt (all) n=6, 63.23y (152)
ref (female) n=146, 64.78y
ref (all) n=146, 64.78y
ref/alt (female) n=3, 61.67y
ref/alt (all) n=3, 61.67y
ref (male) n=235, 72.44y
ref (female) n=291, 71.94y
ref (all) n=526, 72.17y
ref/alt (male) n=5, 80.04y
ref/alt (female) n=1, 75-80y
ref/alt (all) n=6, 79.62y (337)
ref (male) n=238, 72.57y
ref (female) n=288, 71.99y
ref (all) n=526, 72.26y
ref/alt (male) n=2, 75.56y
ref/alt (female) n=4, 69.76y
ref/alt (all) n=6, 71.69y (38)
ref (female) n=148, 64.73y
ref (all) n=148, 64.73y
ref/alt (female) n=1, 60-65y
ref/alt (all) n=1, 60-65y
ref (male) n=236, 72.52y
ref (female) n=290, 71.93y
ref (all) n=526, 72.20y
ref/alt (male) n=4, 77.29y
ref/alt (female) n=2, 76.33y
ref/alt (all) n=6, 76.97y (2)
ref (female) n=147, 64.66y
ref (all) n=147, 64.66y
ref/alt (female) n=2, 69.00y
ref/alt (all) n=2, 69.00y
ref (male) n=230, 72.85y
ref (female) n=287, 71.95y
ref (all) n=517, 72.35y
ref/alt (male) n=10, 66.78y
ref/alt (all) n=15, 68.79y
ref/alt (female) n=5, 72.80y (26)
ref (female) n=144, 64.55y
ref (all) n=144, 64.55y
ref/alt (female) n=5, 69.60y
ref/alt (all) n=5, 69.60y

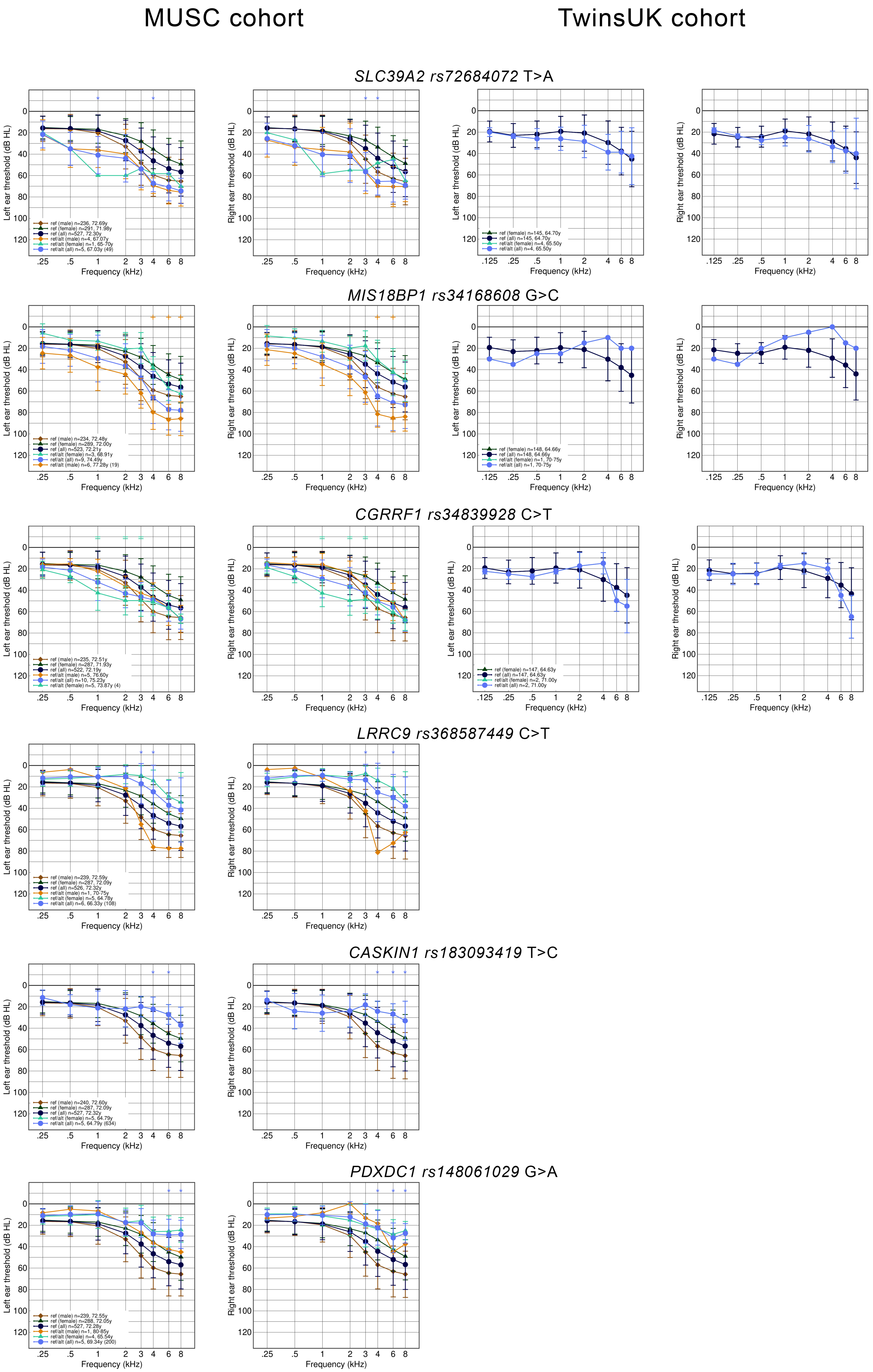
MUSC cohort
TwinsUK cohort
SLC39A2 rs72684072 T>A
ref (male) n=236, 72.69y
ref (female) n=291, 71.98y
ref (all) n=527, 72.30y
ref/alt (male) n=4, 67.07y
ref/alt (female) n=1, 65-70y
ref/alt (all) n=5, 67.03y (49)
ref (female) n=145, 64.70y
ref (all) n=145, 64.70y
ref/alt (female) n=4, 65.50y
ref/alt (all) n=4, 65.50y
MIS18BP1 rs34168608 G>C
ref (male) n=234, 72.48y
ref (female) n=289, 72.00y
ref (all) n=523, 72.21y
ref/alt (female) n=3, 68.91y
ref/alt (all) n=9, 74.49y
ref/alt (male) n=6, 77.28y (19)
ref (female) n=148, 64.66y
ref (all) n=148, 64.66y
ref/alt (female) n=1, 70-75y
ref/alt (all) n=1, 70-75y
CGRRF1 rs34839928 C>T
ref (male) n=235, 72.51y
ref (female) n=287, 71.93y
ref (all) n=522, 72.19y
ref/alt (male) n=5, 76.60y
ref/alt (all) n=10, 75.23y
ref/alt (female) n=5, 73.87y (4)
ref (female) n=147, 64.63y
ref (all) n=147, 64.63y
ref/alt (female) n=2, 71.00y
ref/alt (all) n=2, 71.00y
LRRC9 rs368587449 C>T
ref (male) n=239, 72.59y
ref (female) n=287, 72.09y
ref (all) n=526, 72.32y
ref/alt (male) n=1, 70-75y
ref/alt (female) n=5, 64.78y
ref/alt (all) n=6, 66.33y (108)
CASKIN1 rs183093419 T>C
ref (male) n=240, 72.60y
ref (female) n=287, 72.09y
ref (all) n=527, 72.32y
ref/alt (female) n=5, 64.79y
ref/alt (all) n=5, 64.79y (634)
PDXDC1 rs148061029 G>A
ref (male) n=239, 72.55y
ref (female) n=288, 72.05y
ref (all) n=527, 72.28y
ref/alt (male) n=1, 80-85y
ref/alt (female) n=4, 65.54y
ref/alt (all) n=5, 69.34y (200)
Left ear threshold (dB HL)
Right ear threshold (dB HL)
Frequency (kHz)

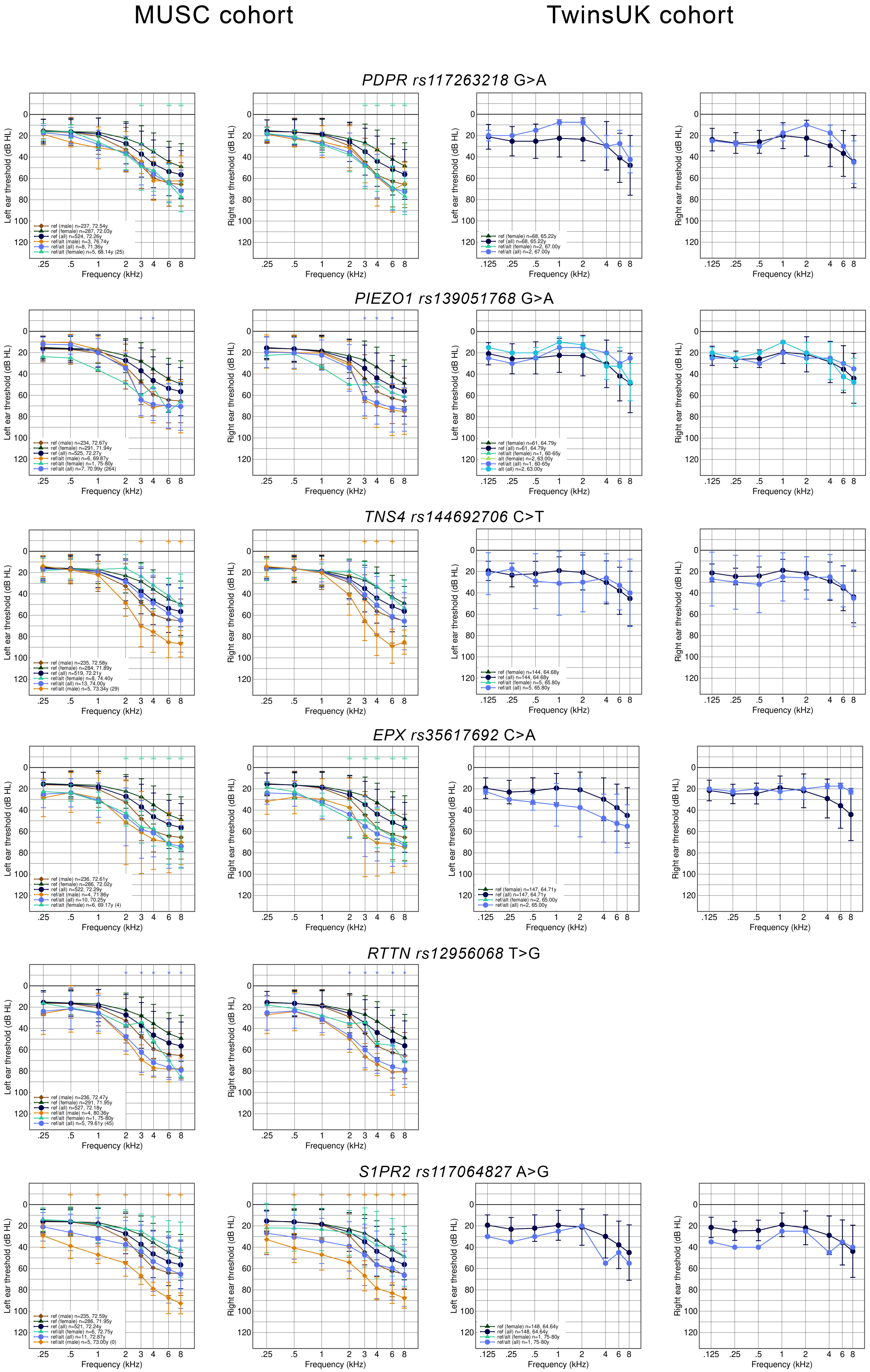

MUSC cohort
TwinsUK cohort
PDPR rs117263218 G>A
Left ear threshold (dB HL)
Right ear threshold (dB HL)
Frequency (kHz)
ref (male) n=237, 72.54y
ref (female) n=287, 72.03y
ref (all) n=524, 72.26y
ref/alt (male) n=3, 76.74y
ref/alt (all) n=8, 71.36y
ref/alt (female) n=5, 68.14y (25)
ref (female) n=68, 65.22y
ref (all) n=68, 65.22y
ref/alt (female) n=2, 67.00y
ref/alt (all) n=2, 67.00y
PIEZO1 rs139051768 G>A
ref (male) n=234, 72.67y
ref (female) n=291, 71.94y
ref (all) n=525, 72.27y
ref/alt (male) n=6, 69.87y
ref/alt (female) n=1, 75-80y
ref/alt (all) n=7, 70.99y (264)
ref (female) n=61, 64.79y
ref (all) n=61, 64.79y
ref/alt (female) n=1, 60-65y
alt (female) n=2, 63.00y
ref/alt (all) n=1, 60-65y
alt (all) n=2, 63.00y
TNS4 rs144692706 C>T
ref (male) n=235, 72.58y
ref (female) n=284, 71.89y
ref (all) n=519, 72.21y
ref/alt (female) n=8, 74.40y
ref/alt (all) n=13, 74.00y
ref/alt (male) n=5, 73.34y (29)
ref (female) n=144, 64.68y
ref (all) n=144, 64.68y
ref/alt (female) n=5, 65.80y
ref/alt (all) n=5, 65.80y
EPX rs35617692 C>A
ref (male) n=236, 72.61y
ref (female) n=286, 72.02y
ref (all) n=522, 72.29y
ref/alt (male) n=4, 71.86y
ref/alt (all) n=10, 70.25y
ref/alt (female) n=6, 69.17y (4)
ref (female) n=147, 64.71y
ref (all) n=147, 64.71y
ref/alt (female) n=2, 65.00y
ref/alt (all) n=2, 65.00y
RTTN rs12956068 T>G
ref (male) n=236, 72.47y
ref (female) n=291, 71.95y
ref (all) n=527, 72.18y
ref/alt (male) n=4, 80.36y
ref/alt (female) n=1, 75-80y
ref/alt (all) n=5, 79.61y (45)
S1PR2 rs117064827 A>G
ref (male) n=235, 72.59y
ref (female) n=286, 71.95y
ref (all) n=521, 72.24y
ref/alt (female) n=6, 72.75y
ref/alt (all) n=11, 72.87y
ref/alt (male) n=5, 73.00y (0)
ref (female) n=148, 64.64y
ref (all) n=148, 64.64y
ref/alt (female) n=1, 75-80y
ref/alt (all) n=1, 75-80y

MUSC cohort

TwinsUK cohort

BRME1 rs77270337 G>A

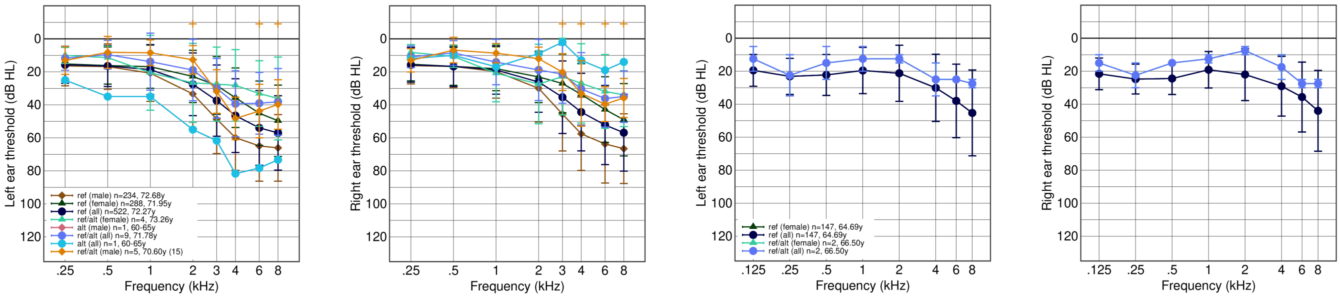

SIGLEC1 rs143489222 C>T

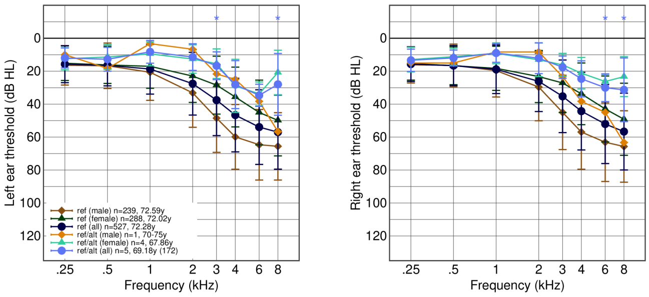

CYP24A1 rs35873579 G>A

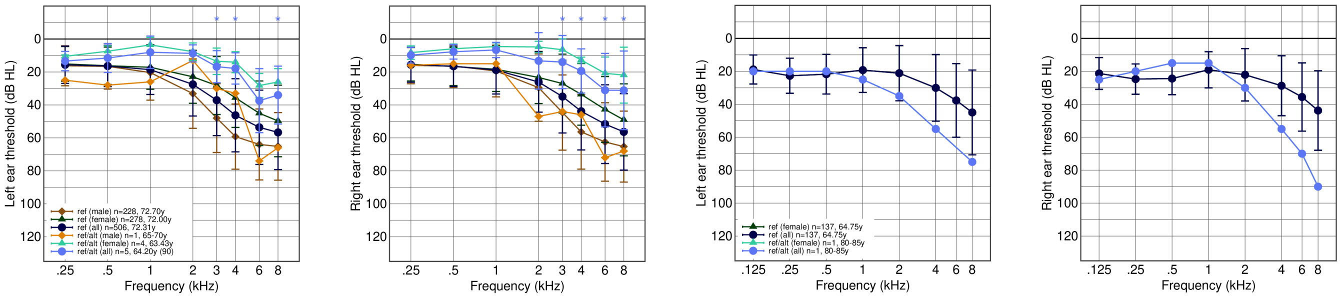

LAMA5 rs78026347 G>A

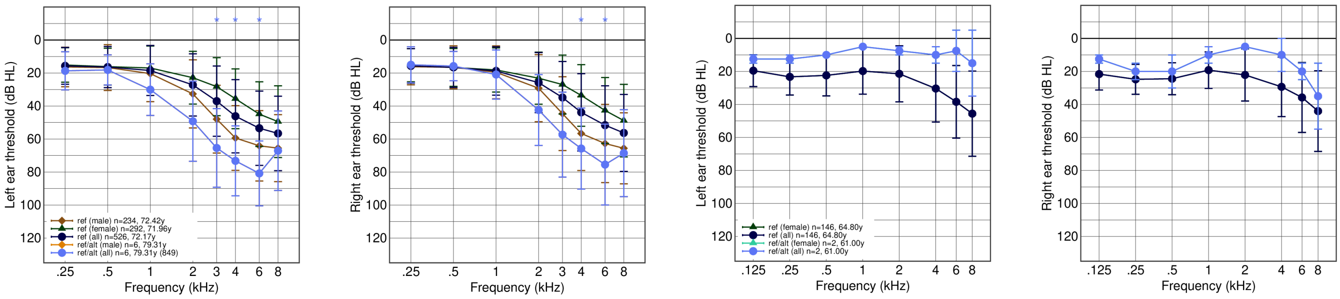

MT-CYB rs41518645 G>A

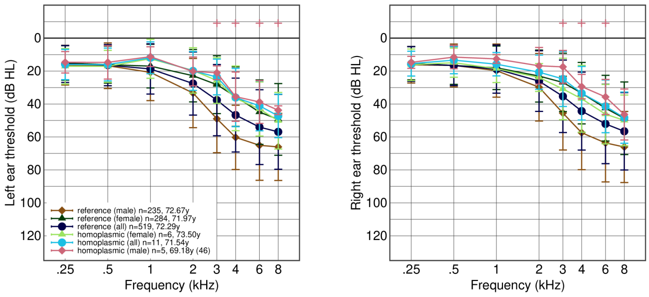

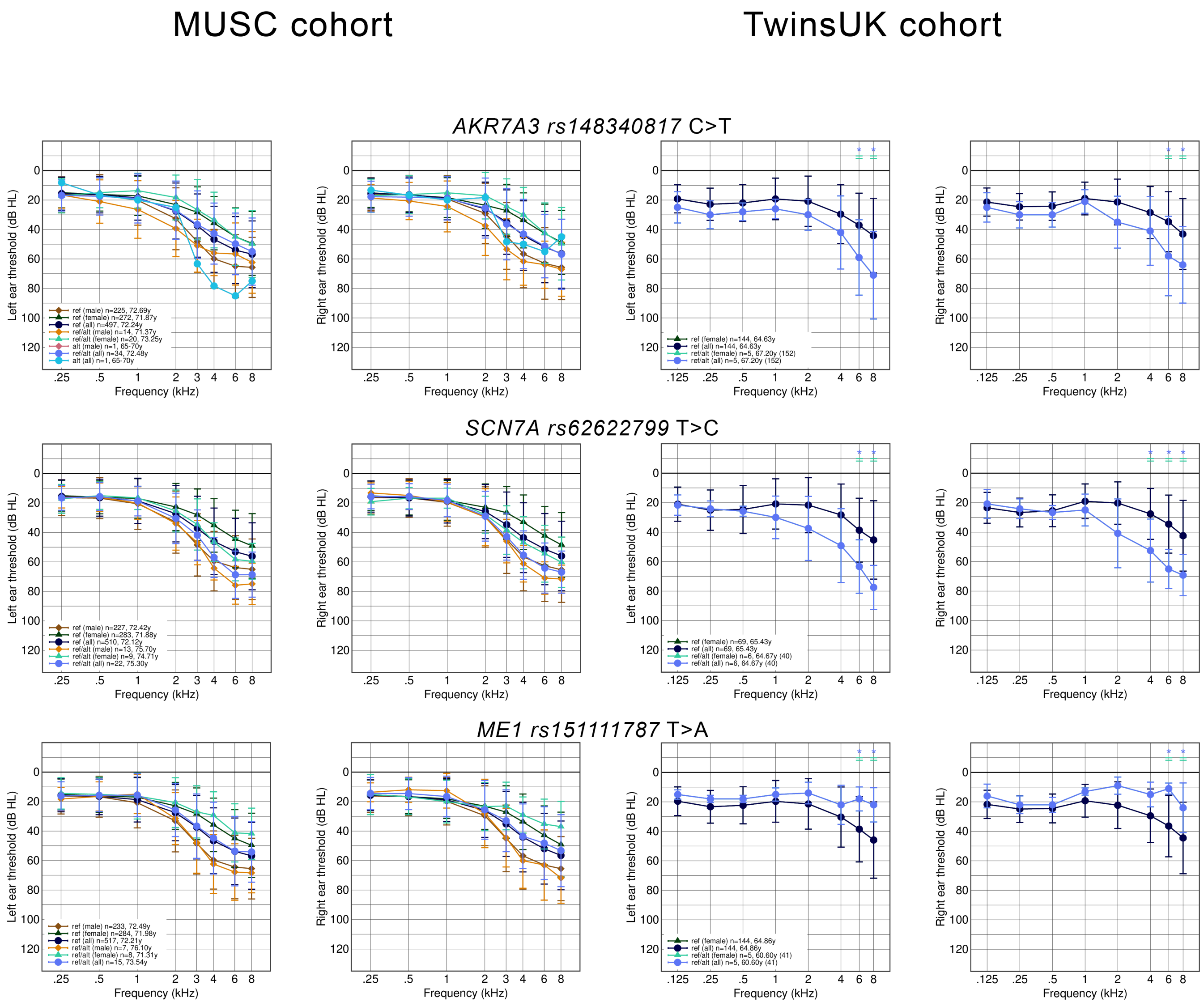

MUSC cohort
TwinsUK cohort
AKR7A3 rs148340817 C>T
ref (male) n=225, 72.69y
ref (female) n=272, 71.87y
ref (all) n=497, 72.24y
ref/alt (male) n=14, 71.37y
ref/alt (female) n=20, 73.25y
alt (male) n=1, 65-70y
ref/alt (all) n=34, 72.48y
alt (all) n=1, 65-70y
ref (female) n=144, 64.63y
ref (all) n=144, 64.63y
ref/alt (female) n=5, 67.20y (152)
ref/alt (all) n=5, 67.20y (152)
SCN7A rs62622799 T>C
ref (male) n=227, 72.42y
ref (female) n=283, 71.88y
ref (all) n=510, 72.12y
ref/alt (male) n=13, 75.70y
ref/alt (female) n=9, 74.71y
ref/alt (all) n=22, 75.30y
ref (female) n=69, 65.43y
ref (all) n=69, 65.43y
ref/alt (female) n=6, 64.67y (40)
ref/alt (all) n=6, 64.67y (40)
ME1 rs151111787 T>A
ref (male) n=233, 72.49y
ref (female) n=284, 71.98y
ref (all) n=517, 72.21y
ref/alt (male) n=7, 76.10y
ref/alt (female) n=8, 71.31y
ref/alt (all) n=15, 73.54y
ref (female) n=144, 64.86y
ref (all) n=144, 64.86y
ref/alt (female) n=5, 60.60y (41)
ref/alt (all) n=5, 60.60y (41)
Left ear threshold (dB HL)
Right ear threshold (dB HL)
Frequency (kHz)

S6 Fig. All audiograms from the MUSC and TwinsUK cohorts plotted in groups by sex and genotype. For the 41 variants identified in the MUSC cohort, audiograms of TwinsUK carriers are shown on the right where available. The variant in *HADH* was identified in both cohorts. For the remaining 3 variants identified in the TwinsUK cohort, the audiograms of MUSC carriers are shown on the left. Two audiograms are shown for each variant in each cohort; the thresholds from the left ear are shown on the left, and those from the right ear on the right. Numbers and average ages of each group are listed on the graph. The symbols at the top of each graph mark which groups passed the criteria for each stimulus frequency compared to the relevant reference group (+ for male, = for female, and * for all participants). Error bars are standard deviation.
