# Supplementary material for: Accurate phenotypic classification and exome sequencing allow identification of novel genes and variants associated with adult-onset hearing loss
Source: PLoS Genet. 2023 Nov 27;19(11):e1011058. doi: 10.1371/journal.pgen.1011058 (PMC10718637; doi:10.1371/journal.pgen.1011058)
Supplement: S7 Fig — A) Schematic showing the classification process and numbers at each stage. B) Plot of the sensory estimate against the metabolic estimate for each well-fit case, with the blue/red shading indicating the magnitude of each estimate. The small dots are the Unclassified cases. C) shows the mean audiograms for the cases assigned to each category (error bars are standard error of the mean). (PDF) [file pgen.1011058.s014.pdf]

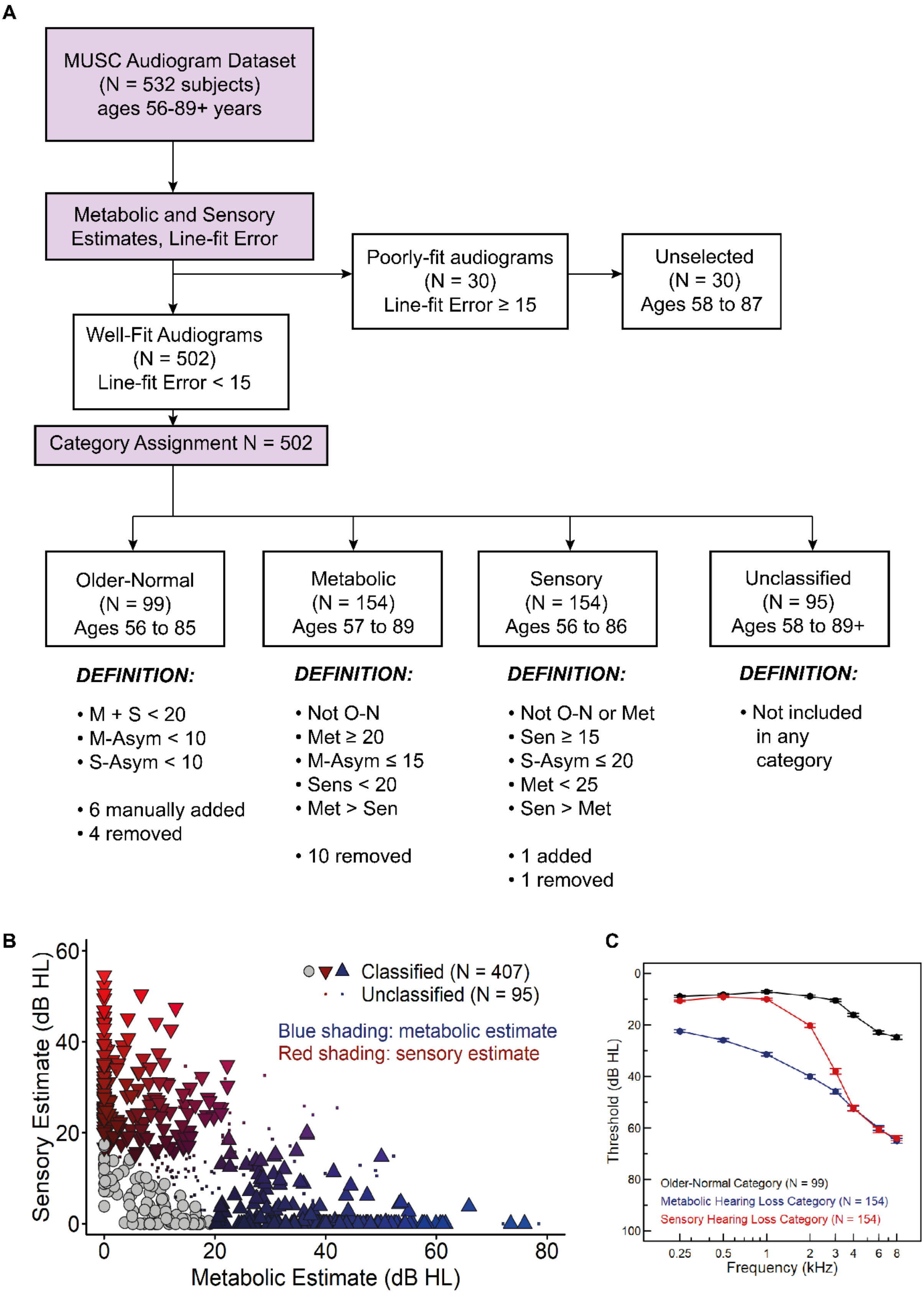
A
MUSC Audiogram Dataset
(N = 532 subjects)
ages 56-89+ years
Metabolic and Sensory
Estimates, Line-fit Error
Poorly-fit audiograms
(N = 30)
Line-fit Error ≥ 15
Unselected
(N = 30)
Ages 58 to 87
Well-Fit Audiograms
(N = 502)
Line-fit Error < 15
Category Assignment N = 502
Older-Normal
(N = 99)
Ages 56 to 85
Metabolic
(N = 154)
Ages 57 to 89
Sensory
(N = 154)
Ages 56 to 86
Unclassified
(N = 95)
Ages 58 to 89+
DEFINITION:
• M + S < 20
• M-Asym < 10
• S-Asym < 10
• 6 manually added
• 4 removed
DEFINITION:
• Not O-N
• Met ≥ 20
• M-Asym ≤ 15
• Sens < 20
• Met > Sen
• 10 removed
DEFINITION:
• Not O-N or Met
• Sen ≥ 15
• S-Asym ≤ 20
• Met < 25
• Sen > Met
• 1 added
• 1 removed
DEFINITION:
• Not included in any category
B
Classified (N = 407)
Unclassified (N = 95)
Blue shading: metabolic estimate
Red shading: sensory estimate
Sensory Estimate (dB HL)
Metabolic Estimate (dB HL)
0
20
40
60
80
C
Threshold (dB HL)
Frequency (kHz)
0.25
0.5
1
2
3
4
6
8
100
Older-Normal Category (N = 99)
Metabolic Hearing Loss Category (N = 154)
Sensory Hearing Loss Category (N = 154)

S7 Fig. MUSC cohort classification. A) Schematic showing the classification process and numbers at each stage. B) Plot of the sensory estimate against the metabolic estimate for each well-fit case, with the blue/red shading indicating the magnitude of each estimate. The small dots are the Unclassified cases. C) shows the mean audiograms for the cases assigned to each category (error bars are standard error of the mean).
