# Supplementary material for: Accurate phenotypic classification and exome sequencing allow identification of novel genes and variants associated with adult-onset hearing loss
Source: PLoS Genet. 2023 Nov 27;19(11):e1011058. doi: 10.1371/journal.pgen.1011058 (PMC10718637; doi:10.1371/journal.pgen.1011058)
Supplement: S8 Fig — A) Schematic showing the classification process and numbers at each stage. B) Plot of the sensory estimate against the metabolic estimate for each well-fit case, with the blue/red shading indicating the magnitude of each estimate. The small dots are the Unclassified cases. C) shows the mean audiograms for the cases assigned to each category (error bars are standard error of the mean). (PDF) [file pgen.1011058.s015.pdf]

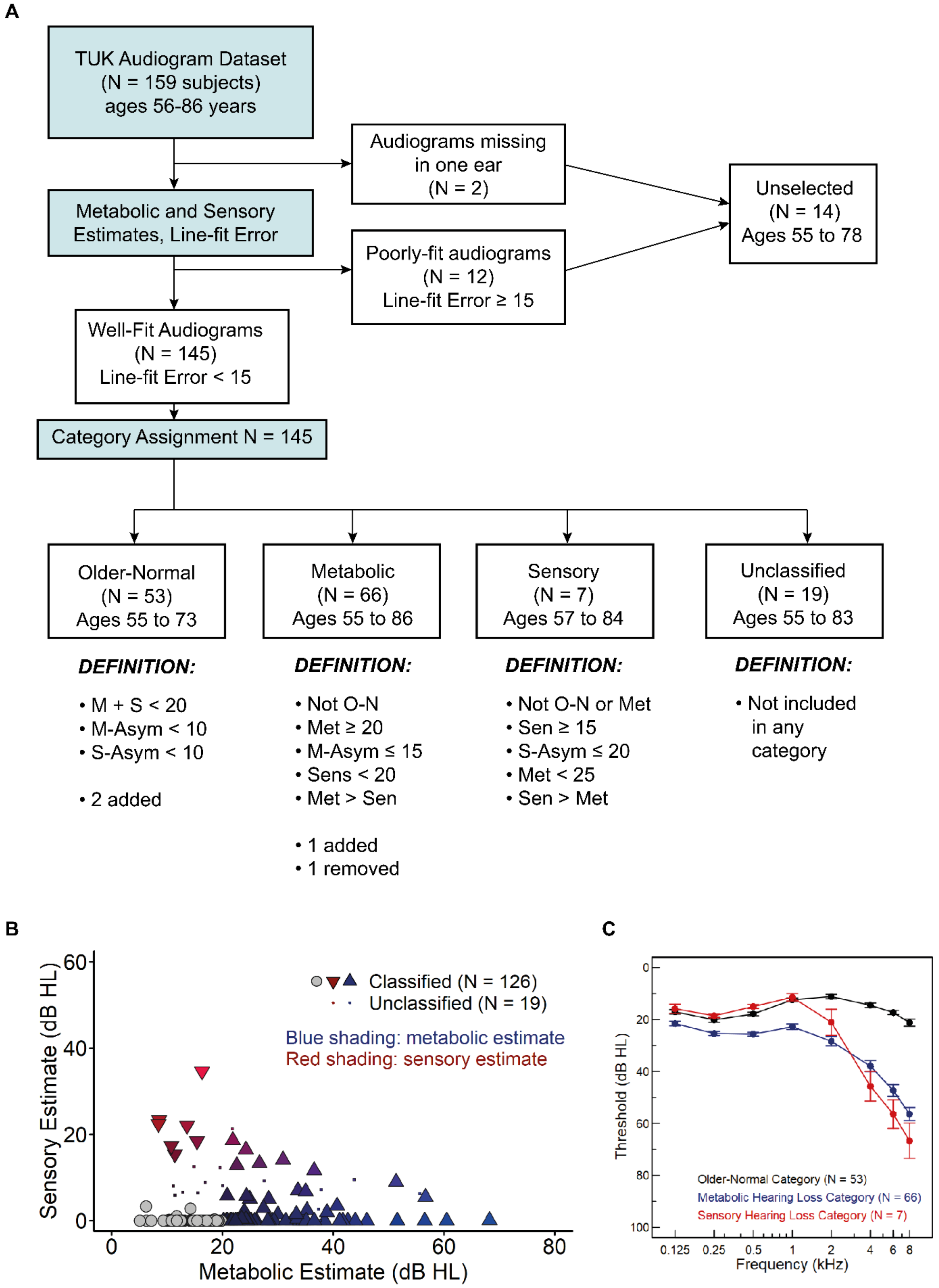
A
TUK Audiogram Dataset
(N = 159 subjects)
ages 56-86 years
Audiograms missing
in one ear
(N = 2)
Metabolic and Sensory
Estimates, Line-fit Error
Poorly-fit audiograms
(N = 12)
Line-fit Error ≥ 15
Unselected
(N = 14)
Ages 55 to 78
Well-Fit Audiograms
(N = 145)
Line-fit Error < 15
Category Assignment N = 145
Older-Normal
(N = 53)
Ages 55 to 73
Metabolic
(N = 66)
Ages 55 to 86
Sensory
(N = 7)
Ages 57 to 84
Unclassified
(N = 19)
Ages 55 to 83
DEFINITION:
• M + S < 20
• M-Asym < 10
• S-Asym < 10
• 2 added
DEFINITION:
• Not O-N
• Met ≥ 20
• M-Asym ≤ 15
• Sens < 20
• Met > Sen
• 1 added
• 1 removed
DEFINITION:
• Not O-N or Met
• Sen ≥ 15
• S-Asym ≤ 20
• Met < 25
• Sen > Met
DEFINITION:
• Not included in any category
B
Classified (N = 126)
Unclassified (N = 19)
Blue shading: metabolic estimate
Red shading: sensory estimate
Sensory Estimate (dB HL)
Metabolic Estimate (dB HL)
0
20
40
60
80
C
Threshold (dB HL)
0
20
40
60
80
100
Frequency (kHz)
0.125
0.25
0.5
1
2
4
6
8
Older-Normal Category (N = 53)
Metabolic Hearing Loss Category (N = 66)
Sensory Hearing Loss Category (N = 7)

S8 Fig. TwinsUK cohort classification. A) Schematic showing the classification process and numbers at each stage. B) Plot of the sensory estimate against the metabolic estimate for each well-fit case, with the blue/red shading indicating the magnitude of each estimate. The small dots are the Unclassified cases. C) shows the mean audiograms for the cases assigned to each category (error bars are standard error of the mean).
